# Discovery of new Cdc2-like kinase 4 (CLK4) inhibitors *via* pharmacophore exploration combined with flexible docking-based ligand/receptor contact fingerprints and machine learning†

**DOI:** 10.1039/d2ra00136e

**Published:** 2022-04-05

**Authors:** Mai Fayiz Al-Tawil, Safa Daoud, Ma'mon M. Hatmal, Mutasem Omar Taha

**Affiliations:** Department of Pharmaceutical Sciences, Faculty of Pharmacy, University of Jordan Amman 11942 Jordan mutasem@ju.edu.jo; Department of Pharmaceutical Chemistry and Pharmacognosy, Faculty of Pharmacy, Applied Sciences Private University Amman Jordan; Department of Medical Laboratory Sciences, Faculty of Applied Medical Sciences, The Hashemite University PO Box 330127 Zarqa 13133 Jordan

## Abstract

Cdc2-like kinase 4 (CLK4) inhibitors are of potential therapeutic value in many diseases particularly cancer. In this study, we combined extensive ligand-based pharmacophore exploration, ligand–receptor contact fingerprints generated by flexible docking, physicochemical descriptors and machine learning-quantitative structure–activity relationship (ML-QSAR) analysis to investigate the pharmacophoric/binding requirements for potent CLK4 antagonists. Several ML methods were attempted to tie these properties with anti-CLK4 bioactivities including multiple linear regression (MLR), random forests (RF), extreme gradient boosting (XGBoost), probabilistic neural network (PNN), and support vector regression (SVR). A genetic function algorithm (GFA) was combined with each method for feature selection. Eventually, GFA-SVR was found to produce the best self-consistent and predictive model. The model selected three pharmacophores, three ligand–receptor contacts and two physicochemical descriptors. The GFA-SVR model and associated pharmacophore models were used to screen the National Cancer Institute (NCI) structural database for novel CLK4 antagonists. Three potent hits were identified with the best one showing an anti-CLK4 IC_50_ value of 57 nM.

## Abstract 2

Ligand-based pharmacophores, ligand–receptor contact fingerprints, physicochemical descriptors and machine learning were combined to probe binding of potent CLK4 antagonists. GFA-SVR gave the best model. Virtual screening identified 3 nanomolar hits.

## Graphical abstract

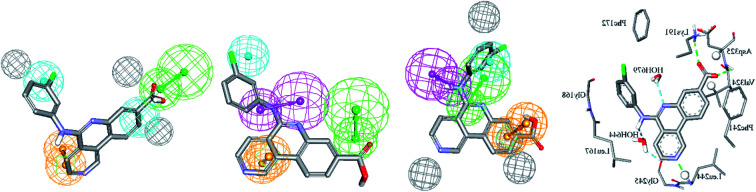

## 1. Introduction

**Fig. 1:**
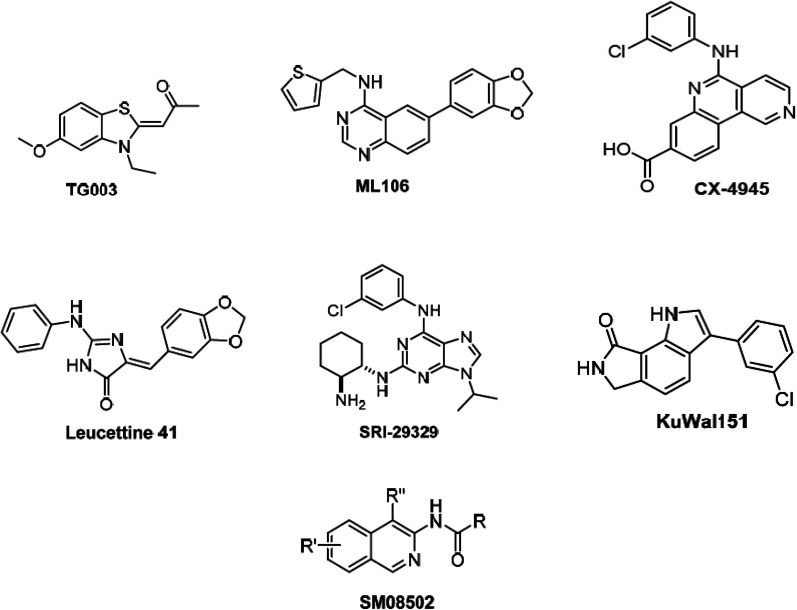
Potent CLK4 inhibitors currently investigated as potential clinical candidates.

Alternative splicing is a deviation from the normal preferred sequence where certain exons are skipped resulting in multiple forms of mature mRNA. This improper splicing contributes to the pathogenesis of many human diseases.^1^ Cyclin dependent-like family kinases (Cdc2-like protein kinases) are dual specific kinases that have been shown to regulate mRNA splicing by phosphorylation of serine and arginine rich proteins.^2^ The Cdc2-like kinases family (CLK) consists of four isoforms CLK1–4 exhibiting the typical protein kinase folds and possessing different lengths of amino acids and they are involved in alternative splicing and RNA processing in Duchenne muscular dystrophy, Alzheimer's disease, HIV-1, influenza virus and cancer.^3^ CLK4 inhibition has recently raised interest as a potential treatment for different CLK4-over expressing cancer types, *i.e.*, renal cancer, breast cancer, melanoma and other cancers. Accordingly, several potent CLK4 inhibitors are currently investigated as potential clinical candidates (Fig. 1),^4^ these include (i) TG003 which is one of the first CLK4 inhibitors published in 2004 with a *K*_d_ value of 30 nM. (ii) ML106 which is a quanzoline-based inhibitor of CLK4 and was published in 2009 with a *K*_d_ value of 50 nM. (iii) CX-4954 which was reported, marketed and used as highly selective and potent inhibitor of casein kinase 2 (CK2). However, CX-4954 also inhibits CLK4 with an IC_50_ of 23 nM. (iv) Leucettine 4 which was developed by modification of natural product leucettamine B found in marine sponge *Leucetta microraphis*. Leucettine 4 inhibits CLK4 with IC_50_ = 64 nM. (v) SRI-29329 which is a CLK4 inhibitor published in 2016 with IC_50_ of 86 nM. (vi) KuWal151 which is a potent CLK inhibitor published in 2018 with IC_50_ of 28 nM against CLK4. (vii) SM08502 which is an isoquinoline based compound, but the exact structure has not been disclosed yet, it is the first CLK inhibitor that entered clinical trials and has an activity against solid tumors with an IC_50_ of 1 nM against CLK4.

Despite the significance of CLK-4 in neurodegenerative disease and cancer-related drug discovery research, it appears that only limited number of earlier computer-aided molecular design and discovery efforts dealing with CLK4 inhibitors were reported. The most prominent was published in 2013 and it involved three-dimensional quantitative structure–activity relationship (3D-QSAR) and pharmacophore modeling to identify ligand structural features required for CLK4 binding.^5^ Nevertheless, the authors failed to validate their models experimentally by *in vitro* bioassays of captured hits. Additionally, their structure-based 3D QSARs were based on homology model of CLK4 rather than the actual crystallographic structural. In another study, bisphenol A was found to act as a ligand for CLK4 among other targets through molecular docking studies.^6^ Moreover, docking studies were performed to optimize several potent CLK4 inhibitors.^7–9^

A pharmacophore is defined as abstract three-dimensional description of binding interactions envisaged to anchor certain ligand within corresponding binding pocket.^10–20,42,43^ The concept of Ligand–Receptor Contacts Fingerprints (LRCFs) is well established.^25^ It proceeds by either mapping out all binding site atoms that contact a list of docked potent ligands and evade inactive ones,^24–26,57^ or identify binding site atoms that frequently contact certain bound ligand during molecular dynamics or related simulations.^47–49^ Machine learning (ML) is the implementation of statistical approaches for learning and predicting properties.^27,28^ Supervised ML attempts to build predictive model(s) based on data collected from input and output sources.^29^ Numerous ML methods have been developed and implemented in the field of drug design and discovery.^39–41^

**Fig. 2:**
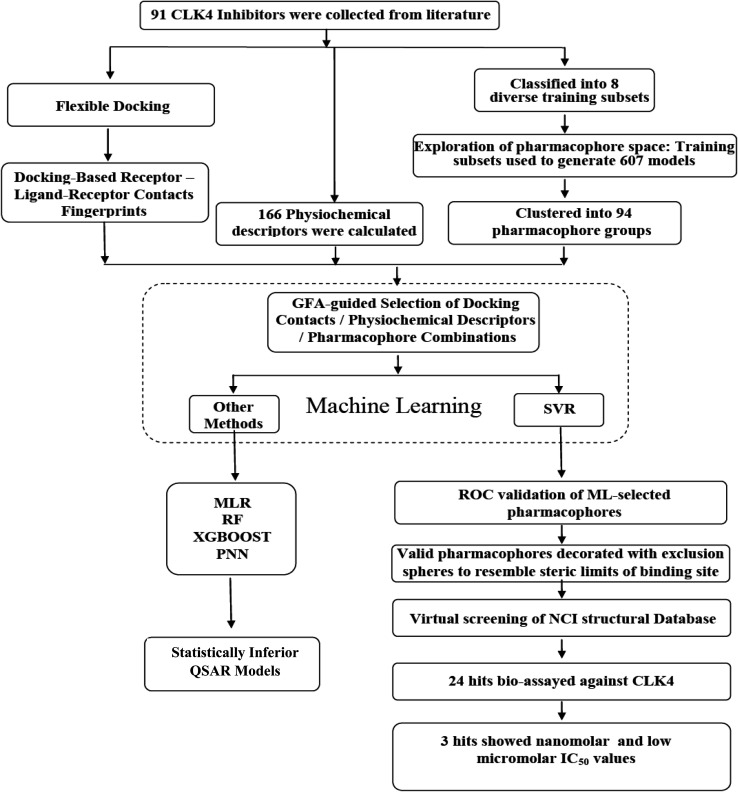
The workflow implemented in the current project.

In the current project, ligand-based pharmacophore modeling was combined with molecular docking to identify novel CLK4 inhibitors. Ligand-based modeling^10–20,42,43^ efforts commenced by extensively exploring the pharmacophoric space of a list of published CLK4 inhibitors (91 molecules) using Discovery Studio (version 4.5, BIOVIA, USA). On other hand, flexible docking^21–23^ was used to dock different inhibitors into the crystallographic structure of CLK4. Then, the docked poses and their corresponding protein conformations were used to generate Ligand–Receptor Contacts Figureprints (LRCFs).^24–26,47–49,57^ Subsequently, LRCFs, ligand-based pharmacophores and numerous other calculatable physicochemical properties were allowed to compete within the context of genetic algorithm coupled to variety of machine learning^27–30^ methods to search for optimal QSAR model(s) that can explain bioactivity variations within training compounds. Thereafter, QSAR-selected pharmacophores were validated by receiver-operating characteristic (ROC) curve analysis and were used as 3D search queries to screen the National Cancer Institute's (NCI) list of compounds for new CLK-4 inhibitors. Fig. 2 summarizes the overall workflow implemented in this study.

## 2. Materials and methods

### 2.1 Molecular modeling of CLK4

#### 2.1.1 Data set collection and conformational analysis

The training compounds were collected from The European Bioinformatics Institute database (ChEMBL) (https://www.ebi.ac.uk/chembl/). However, only compounds of known stereochemistries, published in peer reviewed journal articles with inhibitory bioactivities measured by consistent bioassay procedure in IC_50_ format were selected. Hence, 91 CLK4 inhibitors from published literature were included in the present modelling (1–91 Table S1 under ESI†).^7–9^ The conformations of collected compounds were explored using the “CAESAR conformation generation” option in Discovery Studio (version 4.5, BIOVIA, USA) to represent their conformational flexibilities. CAESAR (Conformer Algorithm based on Energy Screening and Recursive Buildup) has been found to be significantly faster than alternative methods for all data sets investigated.^58^ Conformational ensembles were generated for each molecule with a maximum energy threshold of 20 kcal mol^−1^ from the local minimized structure and a maximum limit of 250 conformers per molecule based on the generalized CHARMm force field implemented within Discovery Studio 4.5.

#### 2.1.2 Ligand-based pharmacophore exploration of CLK4 inhibitors

This step was performed as described earlier.^10–20,42,43^ Briefly, the collected list of compounds (1–91, Table S1 under ESI†) was broken down into training subsets of bioactivity ranges extending over 2 to 2.5 logarithmic cycles. Eight structurally diverse training subsets were carefully selected as in Table S2 under ESI.† The training subsets were selected such that bioactivity differences among their member compounds are related to the presence or absence of pharmacophoric features (*e.g.*, hydrogen bond acceptor (HBA), hydrogen bond donor (HBD), hydrophobic (Hbic), or ring aromatic (RingArom)). Each training subset was selected in such a way to evaluate certain binding hypothesis.^10–20,42,43^ The training subsets were used to explore the pharmacophoric space of CLK4 inhibitors over 64 automatic Discovery Studio CATALYST-HYPOGEN modeling runs.^10–20,42,43^ Different pharmacophores were produced by changing the counts and types of permissible pharmacophoric features and their inter-feature distances in the resulting models (Table S3 under ESI†). Pharmacophore exploration yielded 607 models of variable qualities.

Successful models were clustered into 60 groups (*i.e.*, at ratio of 10 to 1) using the hierarchical average linkage method implemented in CATALYST-HYPOGEN. Subsequently, the highest-ranking representatives, based on their *F*-values (calculated from the correlation of fit values against the bioactivities for the total list of collected CLK4 inhibitors), were selected as representatives in subsequent QSAR modeling.^10–20,42,43^ This was done to minimize collinearity among pharmacophoric descriptors so as to improve noise-to-signal ratio in subsequent ML modeling.^31,32^ However, in case if a particular cluster included more than 10 pharmacophores, then it was represented by additional pharmacophores to maintain the same group-to-representative ratio (*i.e.*, 10-to-1). Table S4 under ESI† shows information about representative pharmacophores including their features, success criteria, differences from corresponding null hypothesis and Cat.Scramble confidence levels (*Y*-scrambling method that challenges CATALYST-HYPOGEN to generate pharmacophore models of superior qualities from scrambled bioactivity data compared to the original unscrambled training sets).^10–20,42,43^

#### 2.1.3 Flexible docking and ligand–receptor contacts fingerprints

##### Preparation of CLK4 crystal structure

Searching the protein databank yielded a single crystallographic structure for CLK4 (25-November-2020, PDB code: 6FYV, resolution = 2.46 Å). Hydrogen atoms were added to the protein utilizing Discovery Studio 4.5 templates for protein residues. The protein structure was utilized in subsequent docking experiments without energy minimization. Explicit water molecules were kept.

##### Ligand docking and scoring

Docking experiments were conducted employing flexible docking as implemented within Discovery Studio 4.5. A binding site sphere of 7.9 Å radius surrounding the center of the co-crystallized ligand (silmitasertib, PDB code: 3NG)^33^ was used to define the binding site. Docking was performed with ligands in their unionized states (only two training compounds are ionizable, namely, 88 and 91 in Table S1 under ESI,† however they were docked unionized). The implemented docking procedure combines features from LibDock^21,22^ and CDOCKER^34^ docking engines. Flexible docking simulates protein flexibility and docks ligands with an induced fit receptor optimization procedure. The following steps are performed: (i) calculate receptor side-chain conformations; initially, the protocol creates protein side-chain conformations using the ChiFlex algorithm. This algorithm generates ensemble of low energy protein conformations with varied side-chain rotamers^35^ (ii) create ligand conformations (similar to the method in Section 2.1.1) (iii) perform initial placement of the ligand conformations into the active site of each receptor side-chain conformation using LibDock docking engine (iv) clustering to remove similar ligand poses (ligand poses are clustered regardless of the protein conformation since the protein conformations are rebuilt during the next step) (v) refine selected protein side-chains in the presence of the rigid ligand using ChiRotor algorithm. This algorithm proceeds by removing side-chain atoms, then perform side-chain conformation sampling, followed by assembling each side chain based on lowest energy conformation followed by final minimization^35^ (vi) perform a final ligand refinement using CDOCKER docking engine. In the current project, amino acids within 7 Å from the bound crystallographic ligand (silmitasertib, PDB code: 3NG) were allowed to be flexible during docking, namely, Leu167, Gly168, Phe172, Val175, Ala189, Lys191, Val225, Phe241, Leu244, Gly245, Ser247, Glu292, Asn293, Leu295, Val324, and Asp325. These should be directly involved in binding interactions with binding ligands, and therefore, susceptible to conformational flexibility (*i.e.*, induced fit).

##### LibDock docking engine

The site-feature docking algorithm (LibDock) docks ligands, after removing their hydrogen atoms, into a putative active site guided by binding hotspots. The ligands' conformations are aligned to polar and apolar receptor interactions sites (*i.e.*, hotspots).^21,22^ The following LibDock parameters were implemented in the current study: the number of binding site hotspots (polar and apolar) was set to 100. The ligand-to-hotspots matching RMSD tolerance value was set to 0.25 Å. The maximum number of poses saved for each ligand during hotspots matching before final pose minimization = 100. Maximum number of poses to be saved for each ligand in the binding pocket = 100. Minimum LibDock score (poses below this score are not reported) = 100. Maximum number of rigid body minimization steps during the final pose optimization (using BFGS method) = 50. Maximum number of steric clashes allowed before the pose-hotspot alignment is terminated (specified as a fraction of the heavy atom count) = 0.1. Maximum value for nonpolar solvent accessible surface area for a particular pose to be reported as successful = 15.0 Å^2^. Maximum value for polar solvent accessible solvent area for a particular pose to be reported as successful = 5.0 Å^2^. No final ligand minimization was implemented.

##### CDOCKER docking engine

CDOCKER is a CHARMm-based simulated annealing/molecular dynamics method for docking ligands into potential receptors.^34^ The following CDOCKER parameters were implemented in the presented project: starting ligands' conformers were energy-minimized then heated to 1000 K over 1000 molecular dynamics steps to generate 10 starting random conformations for each ligand. Each random conformer was rotated 10 times within the binding pocket for subsequent energy refinement. The van der Waals energies of the resulting conformers/poses were evaluated and those of ≥300 kcal mol^−1^ were discarded. Surviving conformers/poses were exposed to a cycle of simulated annealing over 2000 heating steps to targeted temperature of 700 K followed by 5000 cooling steps to targeted temperature of 300 K. The docked poses were energy minimized to a final minimization gradient tolerance zero kcal mol^−1^ Å^−1^. Top 10 poses were arbitrary selected and saved for subsequent scoring.

##### Docking-based ligand–receptor contacts fingerprints (LRCFs)

High ranking docked conformers/poses generated by the flexible docking procedure were scored using two scoring functions: LibDock score^21,22^ and CDocker interactions energy.^34^ Taking into account each scoring function in turn, the highest scoring docked ligand–protein complex was chosen for each inhibitor (1–91, Table S1 under ESI†) for subsequent elucidation of LRCFs. This resulted in two sets of docked ligand–CLK4 virtual complexes corresponding to each scoring function (LibDock score and CDocker interactions energy) for each docked ligand (unless the two scoring functions converged on the same docked pose in which case a single pose is used for generating LRCFs). Each virtual complex in each set was evaluated to identify close binding site atoms to docked ligand, *i.e.*, of distance ≤ 2.5 Å. Atomic neighbors that lie closer than this predefined distance threshold are allocated an intermolecular contact value of “one”, otherwise they are given a contact value of “zero”. Distance evaluations were automatically performed employing a tailored-made Fortran-based software.^24–26,47–49^ Accordingly, each docking–scoring configuration is used to build a 2D matrix, such that each matrix is composed of row labels corresponding to docked ligands and column labels corresponding to different binding site atoms. The matrix is filled with binary code, whereby “zeros” correspond to inter-atomic distances that exceed the predetermined threshold (of 2.5 Å) and “ones” for distances below (or equal) the predefined 2.5 Å threshold cutoff. Each row represents the docking-based Ligand–Receptor Contacts Fingerprint (LRCF) of the corresponding docked compound.

#### 2.1.4 ML-QSAR model building

##### Data preparation

The list of collected CLK4 inhibitors was fitted against representative cluster centers pharmacophores (94 models) and their fit values were used as descriptors in QSAR-ML modeling. Fit values are determined by the following equation:

1 Fit = fitted pharmacophore features × *W* [1 − Σ(disp/tol)^2^]

where, fitted pharmacophore features represent the number of pharmacophore features that superimpose (*i.e.*, overlap or map with) respective chemical functions within the fitted compound. *W* is the weight of the corresponding pharmacophore feature spheres. This value is fixed to 1.0 in HYPOGEN-generated models. “disp” is the distance between the center of certain pharmacophoric sphere (feature centroid), and the center of the respective superimposed chemical moiety of the fitted compound. Tol, known as tolerance, is the radius of the pharmacophoric feature sphere. Σ(disp/tol)^2^ is the summation of (disp/tol)^2^ values for all pharmacophoric features that successfully map corresponding chemical functionalities in the fitted compound.^10–20,42,43^

Additional 166 physicochemical descriptors that included numerous physicochemical, topological and fingerprint descriptors^55,56^ were also calculated employing the “Calculate Molecular Properties” protocol implemented within Discovery Studio (version 4.5, BIOVIA, USA). Moreover, the LRCFs of the molecules were added as additional binary descriptors to the ML-QSAR list of compounds. Two sets of LRCFs were used for each compound, namely, those based on LibDock-score scoring function and CDocker interaction energy scoring function. Accordingly, the ML-QSAR matrix included 91 rows corresponding to 91 collected compounds and 361 columns corresponding to the fit values against 94 pharmacophore models, 101 LRCFs and 166 physicochemical descriptors.

A subset of 72 compounds from the total list of modeled inhibitors (1–91, Table S1 under ESI†) was utilized as training list for ML-QSAR modeling. The remaining 19 compounds (*ca.* 20% of the dataset) were employed as external testing subset for validating ML-QSAR models. The test molecules were selected as follows: the collected compounds were ranked according to their IC_50_ values, and then every fifth compound was selected for the test set starting from the high potency end. The selected test molecules are marked with asterisks in Table S1 under ESI.†

##### ML-QSAR modelling

Genetic function algorithm (GFA) was used to select combinations of pharmacophore fit values, LRCFs and physicochemical descriptors and feed them into the particular machine learner (ML) under evaluation to assess how successful the combination, *i.e.*, descriptors and ML, is in explaining the observed variation in bioactivity (log(1/IC_50_)).^10–20,42,43^ In the current project we implemented the feature selection node within KNIME Analytics Platform (Version 4.1.0) for GFA with the following GFA settings: (i) population size was set to 200 (ii) max number of generations was set to 1000 (iii) fraction of survivors was set to 40% (iv) selection strategy method was “tournament”, where the winner of each tournament is selected to perform crossover (v) elitism rate, which indicates the fraction of the best individuals within a generation that are transferred to the next generation without alternation, was set to 10% (vi) uniform crossover strategy in which each bit (gene) is chosen from either parent with equal probability (vii) crossover rate was set to 60%, accordingly, top 60% of survivors are allowed to mate (viii) mutation rate was set to 1%, accordingly, 1% of the surviving chromosomes in each generation is exposed to mutation, and this is done to escape being captured in a local minimum.

Several ML methods were evaluated to tie these properties with anti-CLK4 bioactivities, namely, multiple linear regression (MLR),^36^ random forests (RF),^37^ extreme gradient boosting (XGBoost),^37,38^ probabilistic neural network (PNN),^39^ and support vector regression (SVR).^40,41^

The best ML method was found to be SVR. This method is a supervised machine learning method that uses the principle ‘kernel trick’ to find number of boundary instances, also called “support vectors”, to create discriminatory function that separates training observations into distinct classes with widest possible boundaries. This function can be used to classify new observations or to predict continuous functions (regression).^40,41^ The following SVR settings were implemented in this project: an upper bound on the fraction of training errors and a lower bound of the fraction of support vectors (*i.e.*, the nu parameter) = 0.664. This value was optimized in this project to minimize the error on training data and reduce the computational complexity of models to avoid over fitting. Other parameters were set to their default values in the Weka-Knime (version 4.1.3) LibSVM node, these include: kernel cache (cache size = 40.0), kernel coefficients epsilon = 0.001 and gamma = 0.00, kernel type is radial basis function: exp(−gamma × |*u* − *v*|^2), and loss function is 0.1.

#### 2.1.5 ML model evaluation

##### Statistical validation of ML-QSAR models

Each ML-QSAR generated model was validated internally, *i.e.*, using the training compounds, and externally, *i.e.*, using the testing list (testing inhibitors are marked with asterisks in Table S1 under ESI†). Internal validation was performed by employing leave one-out cross-validation (*r*_LOO_^2^) and leave-20%-out cross-validation (*r*_L20_^2^%).^42^ Additionally, the models were also validated against the external testing set. Predicted bioactivities of testing compounds (log(1/IC_50_)) values (determined by each ML-QSAR model under evaluation) and their experimental counterparts were used to calculate the predictive *r*^2^ (*r*_PRESS_^2^) defined as:*r*_PRESS_^2^ = (SD − PRESS)/SD2where SD is the sum of the squared deviations between the biological activities of the test set and the mean activity of the training set molecules, PRESS is the squared deviations between predicted and actual activity values for every molecule in the test set.

##### Validation of generated pharmacophore models using receiver-operating characteristic (ROC) curve analysis and goodness of hit score

ML-QSAR selected pharmacophores were validated using ROC curve analysis and goodness of hit list (GH scoring).^59–63^ The testing set is composed of experimentally validated active and inactive CLK4 inhibitors extracted from ChEMBL database (https://www.ebi.ac.uk/chembl/) and not included in the modelling list (91 compounds in ESI Table S1†). The ROC set includes 383 active compounds (anti-CLK4 IC_50_ ≤ 400 nM) and 270 inactive compounds (anti-CLK4 IC_50_ > 3000 nM). Table S5 under ESI† shows the chemical structures of the testing set compounds in simplified molecular-input line-entry system (SMILES) format, together with their corresponding bioactivities.

ROC analysis evaluates the capability of a particular pharmacophore model(s) to correctly classify a group of compounds into actives and inactives. It affords several success criteria for evaluation:^10–20,24–26,42–44^ (i) area under the ROC curve (AUC) of the corresponding ROC curve, (ii) accuracy, (iii) true negative rate, and (iv) true positive rate. True positive rate (or sensitivity, SEN) is calculated by dividing truly active captured hits (true positives) by the sum of true positives and false negatives. True negative rate (or specificity, SPC) is truly inactive compounds being discarded after proper identification. True negative rate is calculated by dividing the true negatives by the sum of true negatives and false positives. Accuracy describes the percentage of correctly classified molecules (active and inactive) by the screening protocol.

GH scoring is calculated as in eqn (2).^59–63^

2 GH = (0.75 Ya + 0.25 SEN)SPC

where, Ya is the percentage yield of actives calculated as the ratio of actives found in the hit list to the total number of compounds in the hit list (Ya = TP/(TP + FP) × 100).

### 2.2 *In silico* screening of the NCI database for new CLK4 antagonists

Pharmacophore hypotheses selected by the best GFA-SVR model, were employed as 3D search queries to screen the National Cancer Institute (NCI) list of compounds. The fit values of captured hits against capturing pharmacophore models and relevant physicochemical molecular descriptors (*i.e.*, that emerged in the optimal GFA-SVR model) were calculated. Additionally, captured hits were docked into the CLK4 binding site (PDB code: 6FYV, resolution = 2.46 Å) using flexible docking with same docking settings employed for modeled compounds to determine their LRCFs. All descriptors were then fed into the optimal GFA-SVR model to determine the predicted bioactivities for the captured hits. The highest-ranking hits were acquired for subsequent *in vitro* testing.

### 2.3 Biological evaluation of captured hits

The bioassay was conducted using LanthaScreen Eu kinase binding assay (Invitrogen-Life Technologies, USA).^45^ This assay is based on the ability of the potential kinase inhibitor under evaluation to bind and displace a proprietary “tracer” molecule (known as Alexa Fluor 674 conjugate) from the catalytic site of the targeted kinase. In case the tested molecule is of low affinity to the targeted kinase, and thus fails to displace the “tracer” molecule, then the tracer remains within the kinase binding site and maintains effective florescent interaction with certain Eu-labeled anti-tag antibody attached at the kinase surface, thus emitting significant fluorescence (time-resolved fluorescence energy transfer, TR-FRET). On the other hand, if the potential inhibitor binds tightly to the kinase, then it will displace the “tracer” molecule from the binding site causing loss of the TR-FRET interaction, *i.e.*, resulting from tracer/Eu interaction, with concomitant loss of fluorescence. Stock solutions of hit molecules were prepared in DMSO, and then serially diluted in assay buffer 50 mM HEPES pH 7.5, 0.01% BRIJ-35, 10 mM MgCl_2_, 1 mM EGTA to yield final hit concentrations of 10 μM. The reaction of the test compound solution, kinase antibody mixture and tracer were mixed and incubated over 1 hour at room temperature, and then the fluorescence was read at *λ*s 665 and 615 nm.^46^ DMSO did not exceed 1% in the final kinase reaction. Hits that inhibited CLK4 more than 75% at 10 μM were further tested at 1.0, 0.10 and 0.01 μM to determine their IC_50_ values. Staurosporine was used as standard (positive control) with IC_50_ value = 7.45 nM. IC_50_ values were calculated using nonlinear regression of the log(concentration) *vs.* inhibition percentage values using GraphPad Prism 5.0.

## 3. Results and discussion

### 3.1 Molecular modeling of CLK4 inhibitors

A total of 91 CLK4 kinase inhibitors were used to generate 94 unique binding pharmacophores as described earlier.^10–20,42,43^ The fact that the pharmacophore exploration phase led to numerous possible CLK4 binding models meant it is hard to decide what is (are) the pharmacophore(s) that best explain the observed anti-CLK4 bioactivities among collected inhibitors. Additionally, exclusive reliance on pharmacophore models generally fails to account for the steric constrains of the binding pocket and bioactivity enhancing/detrimental effects associated with electron-donating and/or withdrawing chemical groups.^10–20,42,43^ These pitfalls cause pharmacophore model(s) to erroneously capture many inactive molecules, which despite their abilities to project their chemical binding groups appropriately into the pharmacophoric features, they either fail to electronically complement the binding site moieties or (and) sterically clash with some binding site features.^10–20,42,43^ Accordingly, additional physicochemical descriptors and LRCFs, determined based on flexible docking, were enrolled as additional descriptors in ML-QSAR modeling. Flexible docking should cover ligands/receptor electronic and steric match/mismatch factors not accounted for by pharmacophore models.^47–49^ GFA analysis was used as to select optimal combination of pharmacophore(s), physicochemical descriptors and LRCFs capable of explaining bioactivity variation among training compounds within the context of the evaluated ML methods.

### 3.2 Supervised ML-QSAR modeling of CLK4 inhibitors

The current project involves supervised ML, whereby elaborate search was performed to identify the best ML method that yields optimal self-consistent and predictive regression model connecting combination of pharmacophore models, LRCFs and physicochemical descriptors with anti-CLK4 bioactivities. However, the fundamental problem in ML regressors is their failure to infer information about the exact descriptors that control variation in the response (bioactivity) of the training observations (training compounds).^50^ Still, MLR and RF are noticeable exceptions as it is possible to identify significant contributors (*i.e.*, descriptors) by either numerical contribution of individual descriptors in the regression model of MLR or by repetitive emergence of certain descriptor(s) as decision criterion in decision trees within RF.^51^ Nevertheless, to solve this issue with other ML methods, it was decided to combine evaluated ML methods with GFA for feature-selection^51^ to identify the most successful combination of pharmacophore(s), physicochemical descriptors and LRCFs that control the bioactivity variation within the training compounds. Several ML modeling approaches were attempted, namely, MLR, SVR, XGBoost, RF, and PNN. All GFA-ML models were validated by leave-20%-out and leave-one-out cross-validation, and against external testing set.^52,53,57^Table 1 shows the results. Clearly, GFA-SVR^40,41^ and GFA-RF^51^ were most consistent in explaining training and testing bioactivity variations. However, the GFA-SVR model scored better leave-one-out crossvalidation *r*^2^ prompting us to select this model for hit identification and bioactivity prediction.

**Table 1:** Correlation coefficient values of best ML-QSAR regression models

ML method	Selected model descriptors^a^^,^^b^^,^^c^	*r* ^2^ d	*r*_L20%_^2^ e	*r*_LOO_^2^ f	*r*_PRESS_^2^ g
GFA-SVR	Hypo(5-R2-08), Hypo(6-R2-07), Hypo(8-R3-08), LEU_244_HN^LD^, VAL_324_HB^LD^, ASP_325_HA^LD^, CHI_2, Num_Rings6	0.91	0.65	0.66	0.76
GFA-RF	Hypo(5-R2-08), Hypo(6-R2-03), Hypo(8-R3-08), Hypo(2-R5-05), VAL_324_HB^LD^, ASP_325_HA^LD^, CHI_2	0.94	0.63	0.57	0.77
GFA-PNN	Hypo(3-R6-08), Hypo(1-R6-02), LEU_210_HD12^CD^, Num_Rings5, Kappa_3	0.96	0.07	0.01	0.71
GFA-XGBoost	Hypo(5-R2-07), Hypo(6-R2-08), Hypo(8-R2-04), Hypo(2-R5-05), Kappa_3, Dipole_Y	0.96	0.47	0.46	0.75
GFA-MLR	log(1/IC_50_) = + 0.12 Hypo(5-R6-08) + 0.129 Hypo(1-R2-08) − 0.276 LYS_191_HZ2^CD^ − 0.22 VAL_324_ HB^LD^ + 0.433 Num_Rings5 − 0.002 PMI_x − 2.65 Shadow_XYfrac − 1.667	0.65	0.46	0.50	0.53

a Hypo(5-R2-08) is the 8^th^ pharmacophore model generated using training subset 5 (Table S2 under ESI) with the 2^nd^ HYPOGEN run settings (Table S3 under ESI), Hypo(6-R2-07): is the7^th^ pharmacophore model generated using training subset 6 (Table S2) with the 2^nd^ HYPOGEN run settings (Table S3), Hypo(8-R3-08) is the 8^th^ pharmacophore model generated using training subset 8 (Table S2) with the 3^rd^ HYPOGEN run settings (Table S3), Hypo(6-R2-03) is the 3^rd^ pharmacophore model generated using training subset 6 (Table S2) with the 2^nd^ HYPOGEN run settings (Table S3), Hypo(2-R5-05) is the 5^th^ pharmacophore model generated using training subset 2 (Table S2) with the 5^th^ HYPOGEN run settings (Table S3), Hypo(3-R6-08) is the 8^th^ pharmacophore model generated using training subset 3 (Table S2) with the 6^th^ HYPOGEN run settings (Table S3), Hypo(1-R6-02) is the 2^nd^ pharmacophore model generated using training subset 1 (Table S2) with the 6^th^ HYPOGEN run settings (Table S3), Hypo(5-R2-07) is the 7^th^ pharmacophore model generated using training subset 5 (Table S2) with the 2^nd^ HYPOGEN run settings (Table S3), Hypo(6-R2-08) is the 8^th^ pharmacophore model generated using training subset 6 (Table S2) with the 2^nd^ HYPOGEN run settings (Table S3), Hypo(8-R2-04) is the 4^th^ pharmacophore model generated using training subset 8 (Table S2) with the 2^nd^ HYPOGEN run settings (Table S3), Hypo(5-R6-08) is the 8^th^ pharmacophore model generated using training subset 5 (Table S2) with the 6^th^ HYPOGEN run settings (Table S3), Hypo(1-R2-08) is the 8^th^ pharmacophore model generated using training subset 1 (Table S2) with the 2^nd^ HYPOGEN run settings (Table S3). Table 2 shows the *X*, *Y*, *Z* coordinates of pharmacophores Hypo(5-R2-08), Hypo(6-R2-07), and Hypo(8-R3-08).

b LEU244HNLD is the hydrogen atom attached to peptidic N of Leu244 selected by LibDock score scoring function, VAL_324_HB^LD^ is the hydrogen atom attached to beta carbon of Val324 selected by LibDock score scoring function, ASP_325_HA^LD^ is the hydrogen atom attached to alpha carbon of Asp325 selected by LibDock score scoring function. Fig. 3 shows the position of these three atoms within the binding pocket, LEU_210_HD12^CD^ is the hydrogen atom attached to delta carbon of Leu210 selected by CDocker interaction energy scoring function, LYS_191_HZ2^CD^ is one of the hydrogen atoms at the terminal amine on the side chain of Lys191 selected by CDocker interaction energy scoring function.

c Num_Rings6: number of 6-membered rings. CHI_2: second order connectivity index, positively correlated with molecular size, Num_Rings5: number of 5-membered rings. Kappa_3: third order kappa shape index, related to molecular flexibility, Dipole_Y: 3D the calculated magnitude and the *X*-vector component of the molecular dipole moment in debyes as estimated from the partial atomic charges (calculated by Gasteiger method) and atomic coordinates. PMI_x: principle moment of inertia in the *X*-dimension, Shadow_XYfrac area of the molecular shadow in the *XY* plane.^54,55^

d Resubstitution correlation coefficient: the model is trained on the training list and used to predict the bioactivities of the same training set.

e Leave-20%-out correlation coefficient.

f Leave-one-out correlation coefficient.

g Predictive correlation coefficient on the external testing set.

Interestingly, the top ML-QSAR models (GFA-SVR and GFA-RF) converged on several descriptors, namely, Hypo(5-R2-08), Hypo(8-R3-08), VAL_324_HB^LD^, ASP_325_HA^LD^ and CHI_2 indicating their significance in the prediction of anti-CLK4 bioactivity.

Table 2 shows the *X*, *Y* and *Z* coordinates of the three pharmacophores that emerged in the GFA-SVR model, while Fig. 3 shows the three pharmacophores and how they map a crystallographic bound ligand within CLK4.

**Table 2:** *X*, *Y*, *Z* coordinates, weights and tolerances of binding features of pharmacophore models selected by implemented ML methods

Pharmacophore	Definition	Chemical features							
			HBA		Hbic		Hbic	RingArom	
Hypo(5-R2-08)^a^	Weights		2.26		2.26		2.26	2.26	
	Tolerances		1.60	2.20	1.60		1.60	1.60	1.60
	Coordinates	*X*	5.60	7.80	2.93		−0.72	−3.62	−3.80
		*Y*	−0.30	1.78	−1.08		−0.82	−0.86	1.75
		*Z*	−0.002	−0.04	4.00		6.46	0.39	1.86
			HBA		HBD		Hbic	RingArom	
Hypo(6-R2-07)^b^	Weights		1.97		1.97		1.97	1.97	
	Tolerances		1.60	2.20	1.60	2.20	1.60	1.60	1.60
	Coordinates	*X*	−1.37	−0.27	−2.84	−4.35	−0.56	2.23	2.82
		*Y*	−1.58	−2.36	−1.35	−3.68	0.70	−1.44	1.38
		*Z*	−1.25	−3.93	−2.47	−3.62	−4.49	0.53	−0.31
			HBA		HBD		Hbic	RingArom	
Hypo(8-R3-08)	Weights		2.18		2.18		2.18	2.18	
	Tolerances		1.60	2.20	1.60	2.20	1.60	1.60	1.60
	Coordinates	*X*	4.51	3.65	−2.13	0.73	−3.08	−1.42	−1.42
		*Y*	−2.27	−5.16	−1.90	−2.75	−3.82	0.01	2.60
		*Z*	0.06	−0.07	2.84	3.13	6.66	−0.01	1.50

a This pharmacophore includes 3 exclusion spheres of 1.2 Å diameters and at the following *X*, *Y*, *Z* coordinates: (−1.73, −0.05, 9.32), (4.58, 0.33, 2.75), and (3.06, 3.54, −2.21). Exclusion spheres represent regions forbidden for occupancy by ligand groups.

b This pharmacophore includes 4 exclusion spheres of 1.2 Å diameters and at the following *X*, *Y*, *Z* coordinates: (−1.81, 2.02, −7.37), (2.94, 1.12, 2.95), (−3.64, −0.13, 4.64), and (3.49, −5.94, 0.08).

**Fig. 3:**
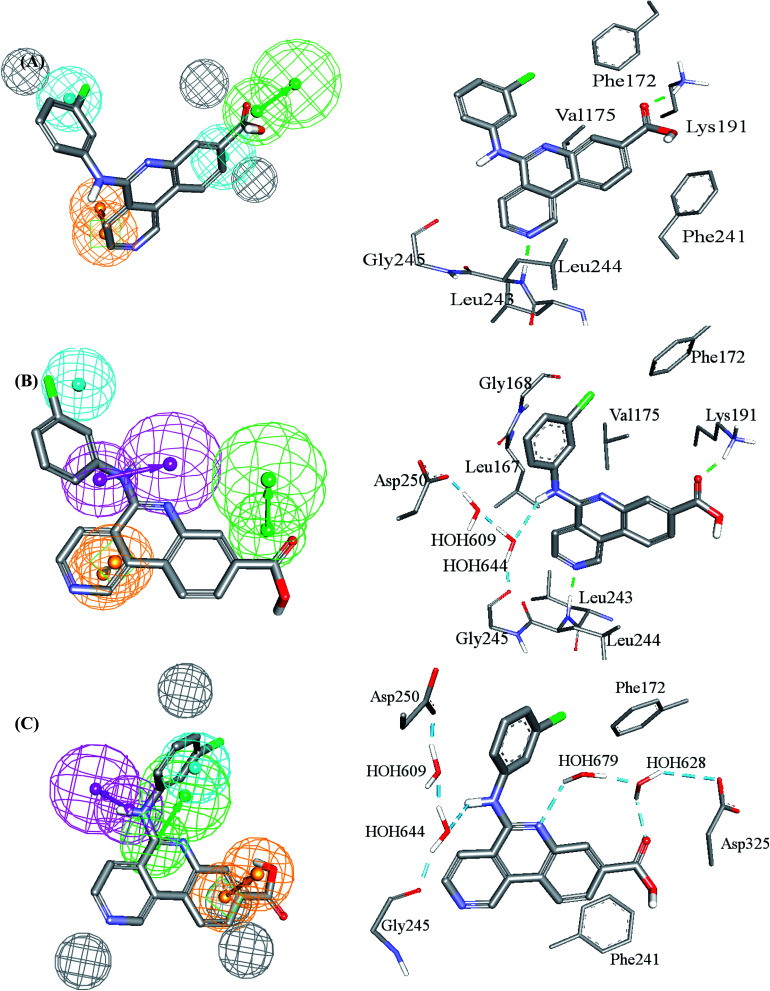
Comparison between pharmacophore models in the best GFA-SVR QSAR model and binding interactions observed within the CLK4 crystallographic complex 6FYV. (A)–(C) Pharmacophore models, Hypo(5-R2-08), Hypo(8-R3-08) and Hypo(6-R2-07), respectively, and how they map the crystallographic bound ligand. Hydrogen bond donor features (HBDs) are shown as pink vectored spheres, hydrogen bond acceptor features (HBA) are shown as green vectored spheres, Aromatic ring features (RingArom) are shown as orange vectored spheres, hydrophobic features (Hbic) are shown as blue spheres, exclusion areas (spheres) are shown as grey spheres.

Clearly from Fig. 3, the GFA-SVR pharmacophores, *i.e.*, Hypo(5-R2-08), Hypo(6-R2-07), and Hypo(8-R3-08), emphasize certain ligand–receptor binding interactions seen in the crystallographic complex: all three pharmacophores highlight a hydrophobic interaction tying the chlorobenzene of the bound ligand with the aromatic side chain of Phe172. However, Hypo(5-R2-08) and Hypo(6-R2-07) emphasize hydrophobic and π-stacking interactions, respectively, connecting the benzoic acid fragment of the bound ligand and the aromatic side chain of phe241 (Fig. 3A and C). Similarly, Hypo(5-R2-08) and Hypo(8-R3-08) highlight hydrogen bonding interaction connecting the carboxylic acid of the bound ligand with the side chain ammonium of Lys191 (Fig. 3A and B). The same pharmacophores, *i.e.*, Hypo(5-R2-08) and Hypo(8-R3-08), highlight π-stacking interactions involving the pyridine side-ring of the bound ligand against the peptidic amide connecting Leu244 and Gly245 (Fig. 3A and B). However, Hypo(8-R3-08) and Hypo(6-R2-07) underscore hydrogen bonding interaction involving the central aniline NH of the bound ligand with a network of water molecules connected to Asp250 and Gly245 (Fig. 3B and C). It is noteworthy to mention that the apparent less-than-optimal mapping of the hydrogen bond donor feature of Hypo(8-R3-08) against the central aniline NH of the crystallographic bound ligand (Fig. 3B) is not unexpected since Hypo(8-R3-08) (and other pharmacophores in the optimal GFA-SVR model) was totally generated through ligand-based process without considering the binding site. Moreover, despite the reliably of crystallographic structures in drug design, they suffer from some serious problems such as inadequate resolution and crystallization-related artifacts of the ligand–protein complex. Furthermore, crystallographic structures generally ignore structural heterogeneity related to protein anisotropic motion and discrete conformational substates.^64^ These factors warrant the use of ligand-based methods as adjuvants to complement information derived from bound ligand poses derived from crystallographic complexes.

Interestingly, Hypo(6-R2-07) uniquely highlights hydrogen bonding interaction involving the central pyridine nitrogen of the bound ligand with a network of hydrogen-bonded water molecules connected to the carboxylic acid side chain of Asp325 (Fig. 3C).

To evaluate the ability of pharmacophore models in the GFA-SVR QSAR to effectively and selectively capture active hits, the three pharmacophores were validated against an external ROC testing set extracted from ChEMBL database. The results are shown in Table 3.

**Table 3:** Receiver operating characteristic (ROC) information of ligand-based pharmacophores

Pharmacophore	AUC%^a^	ACC%^b^	TNR%^c^	TPR%^d^	GH score
Hypo(5-R2-08)	56%	54%	47%	66%	0.43
Hypo(6-R2-07)	57%	57%	49%	64%	0.39
Hypo(8-R3-08)	53%	50%	44%	62%	0.40

a Area under the curve.

b Overall accuracy.

c Overall true negative rate (also known as specificity).

d Overall true positive rate (also known as sensitivity).

**Fig. 4:**
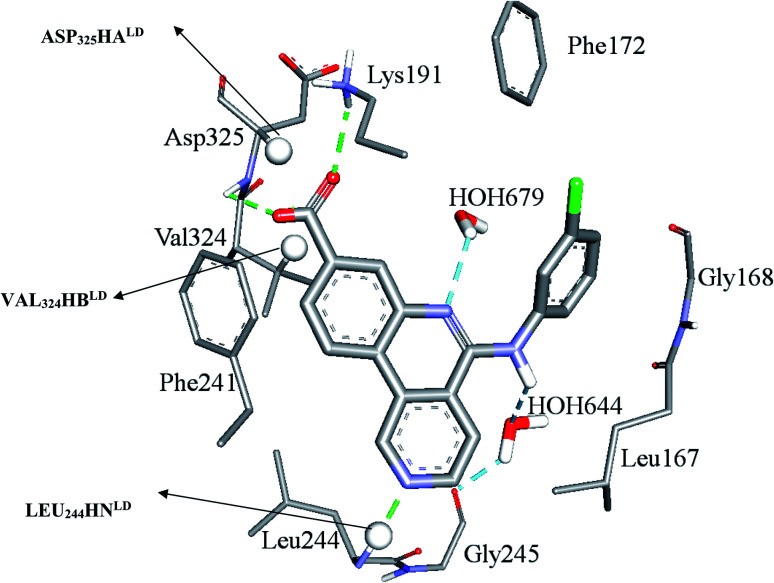
Significant ligand–receptor contacts selected by the GFA-SVR model, Table 1. Significant contacts are shown as spheres. The image represents the crystallographic structure of silmisertib bound to CLK4 (PDB code: 6FYV).

Clearly from Table 3, the three pharmacophores exhibit mediocre performances in discriminating actives from inactive CLK4 inhibitors, as their receiver operating characteristic areas under the curves (ROC-AUCs) ranged from 53% to 57%. Moreover, they exhibited moderate goodness of hit (GH) values.^59–63^ This explains the need for additional descriptors including LRCFs to achieve satisfactory machine learning models. Therefore, unsurprisingly, the GFA-SVR model in Table 1 highlights three significant ligand receptor contacts that emerged from the flexible docking *via* LibDock-score scoring function, namely, LEU_244_HN^LD^, VAL_324_HB^LD^, ASP_325_HA^LD^ shown in Fig. 4. Interestingly, LEU_244_HN^LD^ corresponds to hydrogen-bonding interaction connecting potent ligands to Leu244 (Fig. 4) that is not represented in any of the pharmacophores within the best GFA-SVR model (Fig. 3).

### 3.3 *In silico* screening of the NCI database for new CLK4 antagonists

The fundamental utility of pharmacophores and associated ML models is in the discovery of new chemical scaffolds of similar or even better biological profiles compared to starting training compounds, *i.e.*, scaffold hopping. Therefore, pharmacophores Hypo(5-R2-08), Hypo(8-R3-08) and Hypo(6-R2-07), Fig. 3A–C, respectively, were employed as 3D search queries to screen the National Cancer Institute list of compounds (NCI list, 268 667 compounds) for new CLK4 inhibitors. They captured 777, 626, and 798 hits, respectively. Duplicate hits were removed to yield 1153 unique compounds. These hits were fitted against the three pharmacophores, flexibly-docked into CLK4, and their pharmacophore fit values together with their LRCFs and other relevant physicochemical descriptors were substituted in GFA-SVR model in Table 1 to predict the corresponding bioactivities. Hits of predicted IC_50_ values ≤ 0.5 μM were retained (92 hits), out of which only 24 compounds were available from the NCI and were therefore acquired and tested *in vitro* against CLK4 at 10 μM.

Bioassay was performed using LanthaScreen Eu kinase assay kit (Invitrogen, USA). Staurosporine was used as standard CLK4 inhibitor (positive control, IC_50_ value = 7.45 nM).^46^ ESI Table S6† lists the tested compounds, their descriptors relevant to GFA-SVR model in Table 1 including fit values against Hypo(5-R2-08), Hypo(8-R3-08) and Hypo(6-R2-07) together with their predicted bioactivities and experimental anti-CLK4 inhibition at 10 μM. Hits that illustrated inhibitory percentages exceeding 75% at 10 μM were further evaluated at 1.0, 0.1, 0.01 and 0.001 μM to determine their IC_50_ values. Hits 96, 107 and 109 (Table S6 under ESI†) exceeded 75% CLK4 inhibition at 10 μM warranting further evaluation to determine their anti-CLK4 IC_50_ values.

**Fig. 5:**
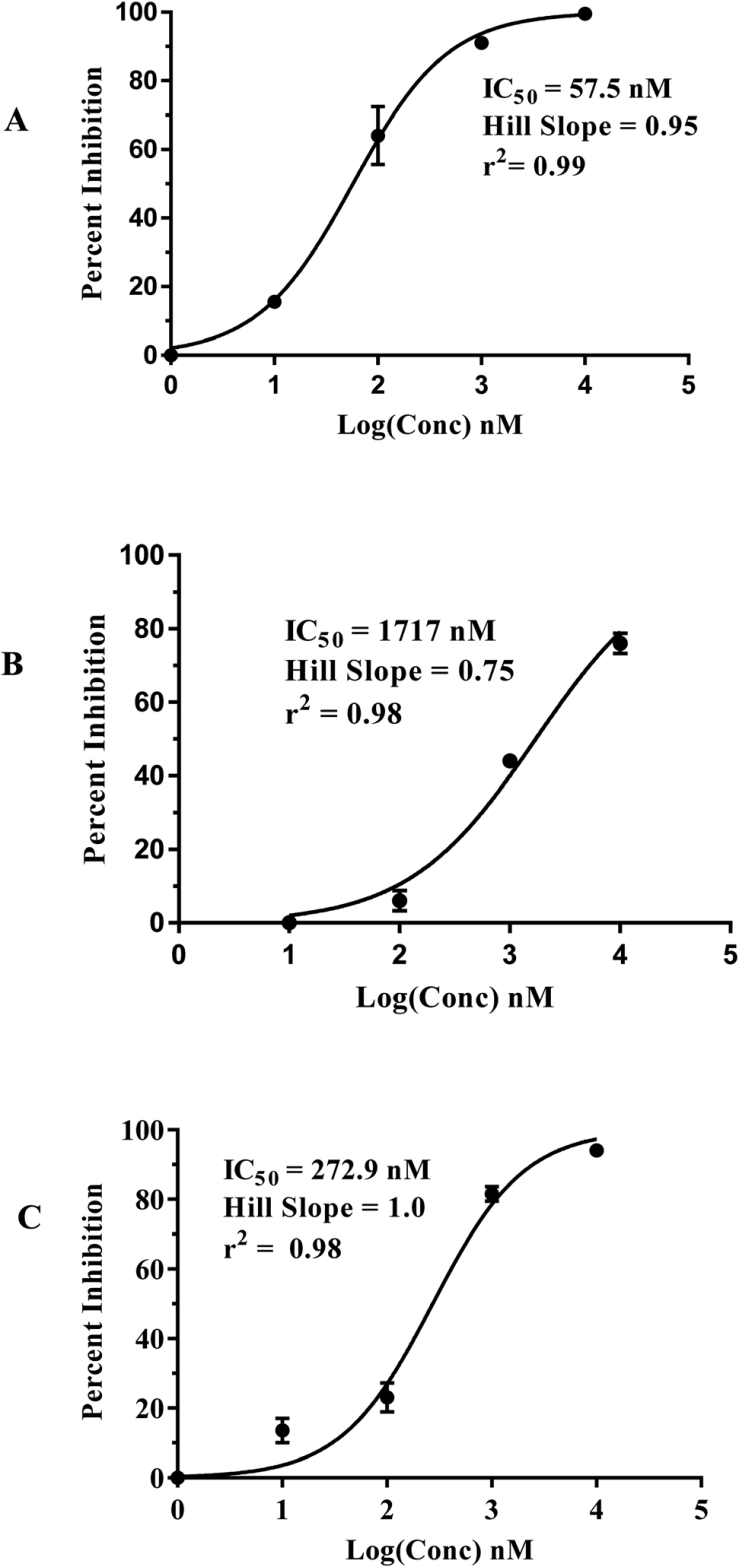
Dose–response curves of hits (A) 96, (B) 107 and (C) 109. The figures also show the corresponding IC_50_ values, Hill slopes and correlation *r*^2^ values.

**Fig. 6:**
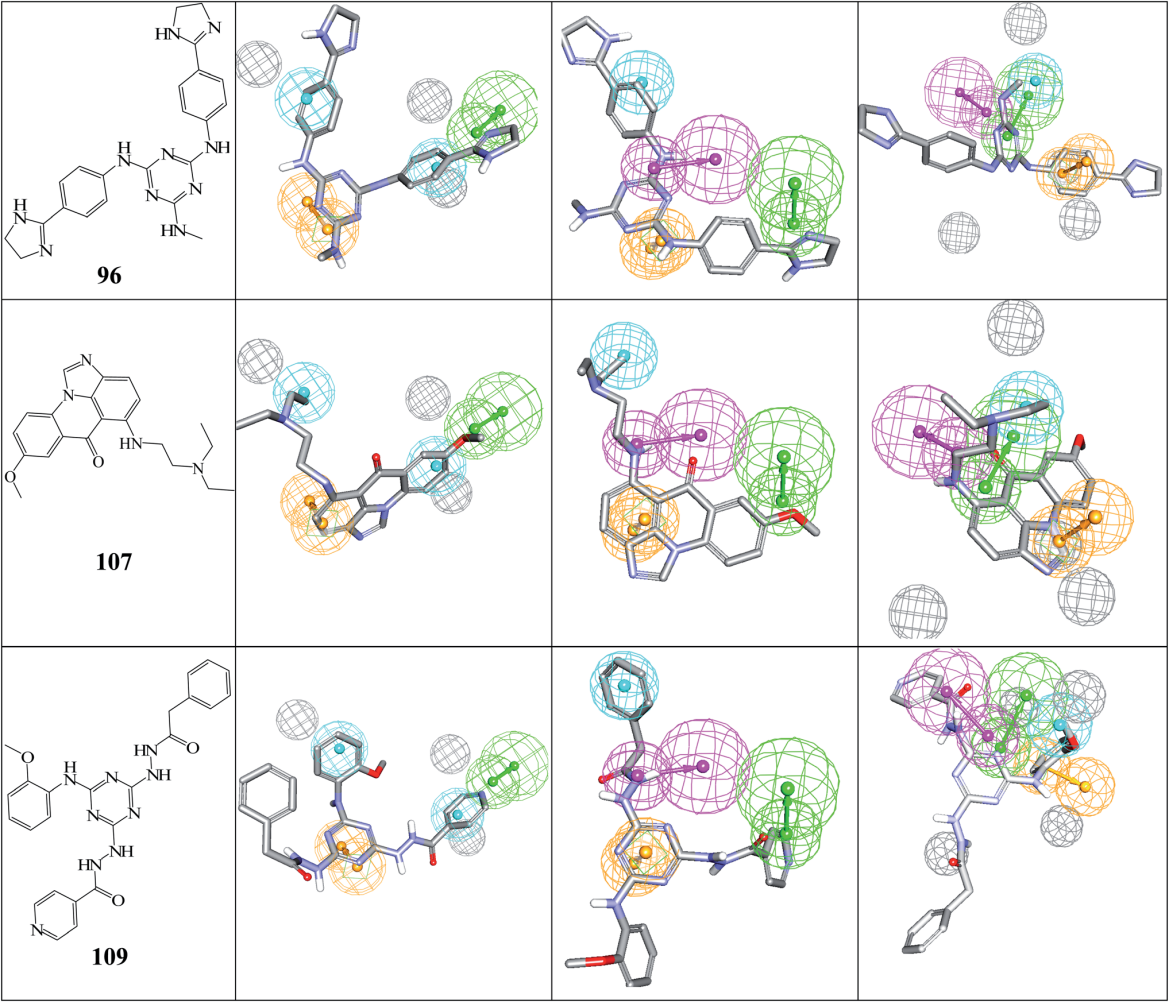
The chemical structures of captured hits (left column) and their mappings against pharmacophores Hypo(5-R2-08), Hypo(8-R3-08) and Hypo(6-R2-07) (respectively, left to right).

Fig. 5 shows the dose–response curves of the three hits, while Fig. 6 shows how the molecules map pharmacophore models Hypo(5-R2-08), Hypo(8-R3-08) and Hypo(6-R2-07). Interestingly the three hits illustrated potent anti-CLK4 inhibitory profiles with the best one, hit 96, illustrating IC_50_ value of 57 nM. The Hill slopes of the three active hits are ≤1.0 suggesting they are authentic (non-promiscuous) inhibitors.^56^

**Fig. 7:**
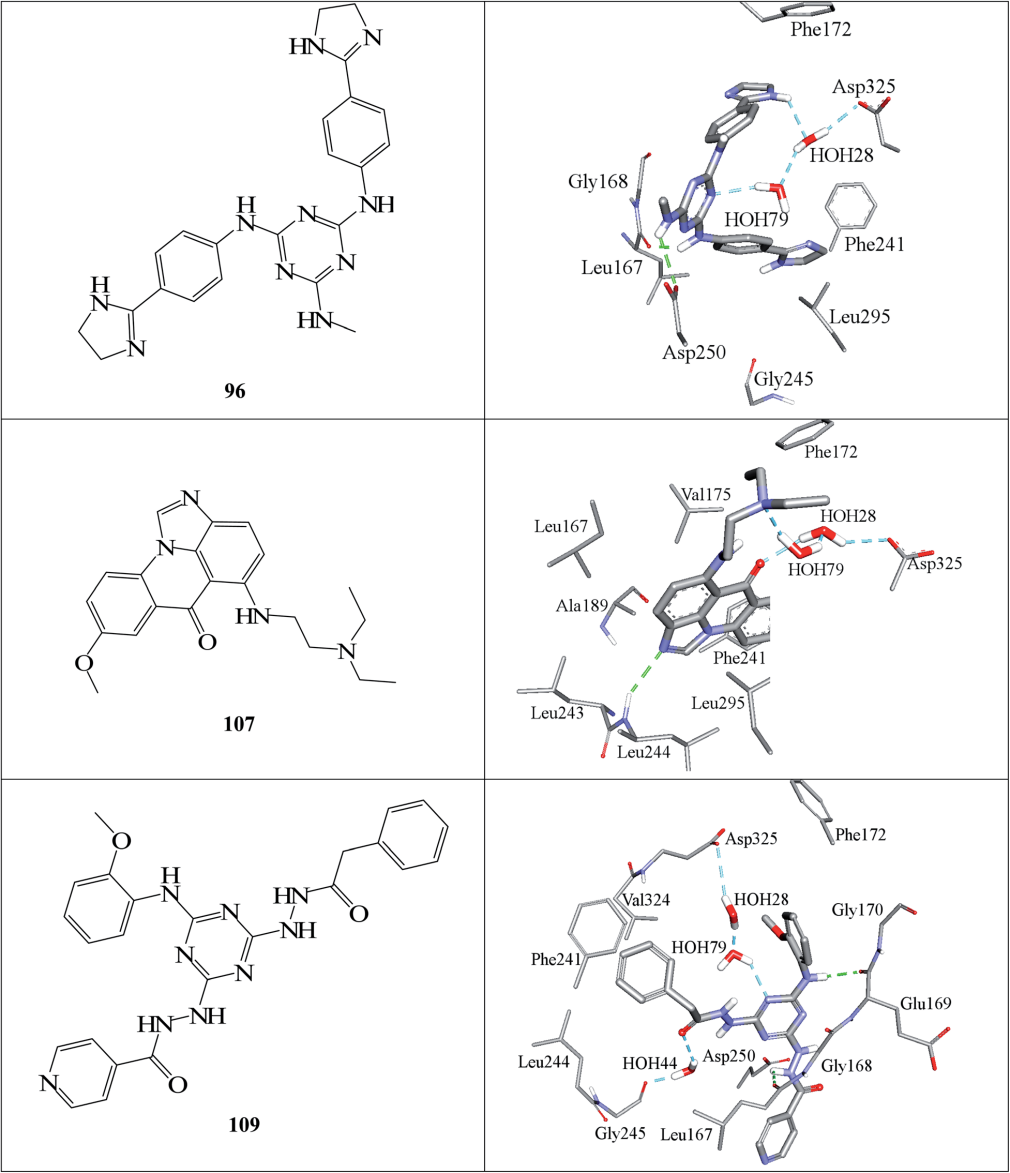
Docked poses of active hits 96, 107 and 109 as generated by successful flexible docking settings.

Fig. 6 and 7 show how active hits fit their respective capturing pharmacophores and how they dock into CLK4, respectively. Mapping the active hits against three pharmacophores seems to emphasize similar binding interactions, namely, hydrogen bonding with a network of water molecules connected to Asp325, hydrophobic interactions with the aromatic side chain of Phe172 and Phe241, as well as π-stacking interactions against the peptidic amide connecting Leu244 and Gly245.

**Fig. 8:**
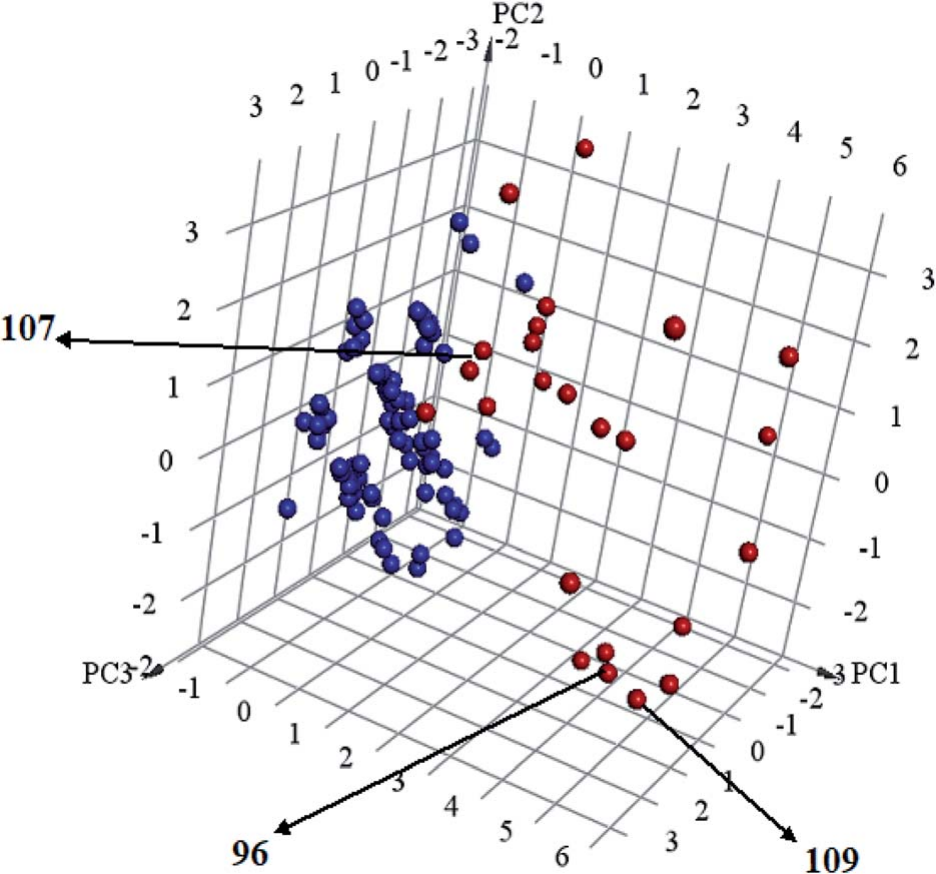
Principal component analysis showing the relative distribution of captured hits (92–115, Table S6;† red spheres 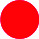) compared to modeled compounds (1–91, Table S1;† blue spheres 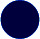). The top three principal components calculated for modeled compounds and captured hits are based on eight descriptors (*i.e.*, log *P*, molecular weight, hydrogen bond donors and acceptors, rotatable bonds, number of rings, number of aromatic rings, fractional polar surface area surface area). The active hits 96, 107 and 109 are indicated in the figure with arrows.

**Fig. 9:**
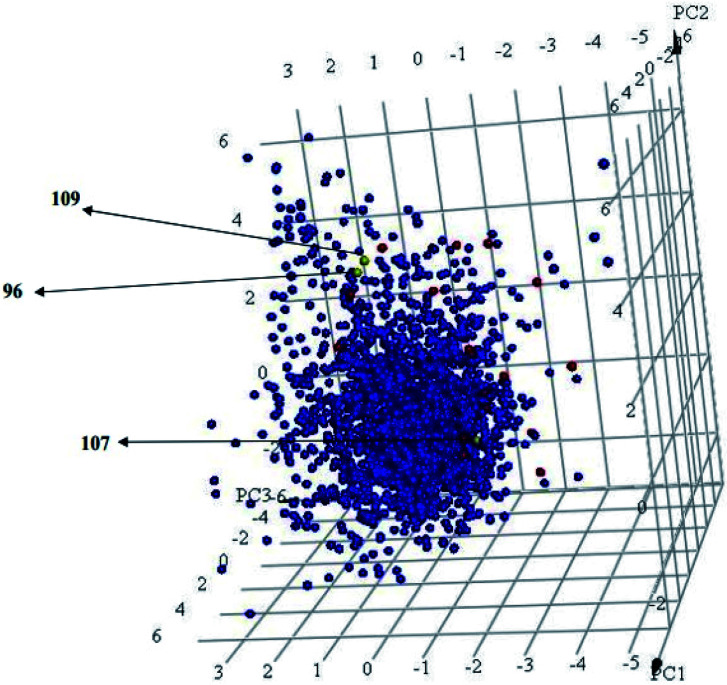
Principal component analysis showing the relative distribution of captured hits (red spheres, 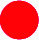) including active hits (yellow spheres, 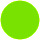) among all reported 3643 CLK4 inhibitors extracted from ChEMBL database (blue spheres, 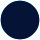). The top three principal components were calculated based on eight descriptors (*i.e.*, log *P*, molecular weight, hydrogen bond donors and acceptors, rotatable bonds, number of rings, number of aromatic rings, fractional polar surface area surface area).

Interestingly, captured hits exhibit significantly distinct chemical scaffolds compared to the modelled list of compounds (1–91, Table S1 under ESI†) and even to all known CLK4 inhibitors, as in Fig. 8 and 9. This conclusion is based on principal component analysis (PCA) (Fig. 8) showing the relative physicochemical distribution and diversity of captured hits (92–115, Table S6 under ESI†) compared to the modeled compounds (1–91, ESI Table S1†), as in Fig. 8, or even to all known CLK4 inhibitors within ChEMBL database, as in Fig. 9. In fact hits 96 and 109 represent the first in class nanomolar CLK4 inhibitors, as this the first time to report potent triazine-based CLK4 inhibitors.

## 4. Conclusions

In conclusion, the combination of pharmacophore modeling of CLK4-antagonists and LRCFs generated by flexible docking followed by GFA-driven ML-based modeling yielded self-consistent and predictive SVR-QSAR model. The resulting pharmacophores were validated by receiver operating characteristic (ROC) curve analysis and used as virtual search queries to screen the National Cancer Institute (NCI) database for promising CLK4 hits of novel chemo-types. Three hits (96, 107 and 109) showed nanomolar and low micromolar IC_50_ values. The three hits represent novel CLK4 inhibitors scaffold.

## Conflicts of interest

There are no conflicts to declare.

## Supplementary Material

RA-012-D2RA00136E-s001
