# Supplementary material for: Discovery of new Cdc2-like kinase 4 (CLK4) inhibitors via pharmacophore exploration combined with flexible docking-based ligand/receptor contact fingerprints and machine learning
Source: RSC Adv. 2022 Apr 5;12(17):10686–700. doi: 10.1039/d2ra00136e (PMC8982525; doi:10.1039/d2ra00136e)
Supplement: RA-012-D2RA00136E-s001 [file RA-012-D2RA00136E-s001.pdf]

Electronic Supplementary Material (ESI) for RSC Advances.
This journal is © The Royal Society of Chemistry 2022

Table S1. The structures of CLK4 inhibitors utilized in modelling.

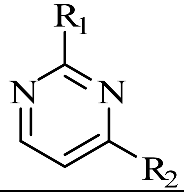

Compound	R_1	R_2	IC_{50} (nM)	Reference
**1**	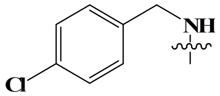	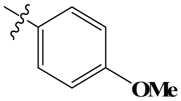	319	7
**2**	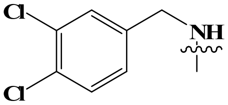	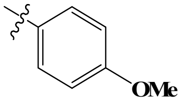	92	7
**3**	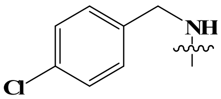	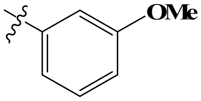	6,806	7
**4**	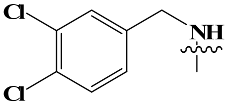	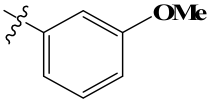	3,858	7
**5**	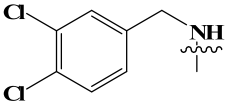	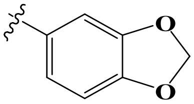	14	7
**6***	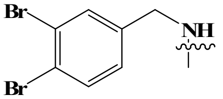	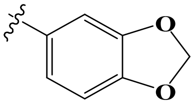	18	7
**7***	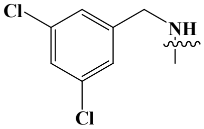	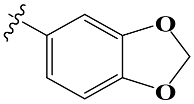	4	7
**8**	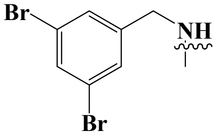	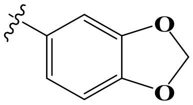	9	7
**9**	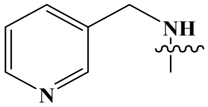	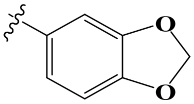	16	7
**10***	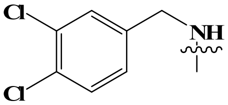	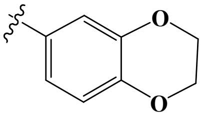	13	7
**11**	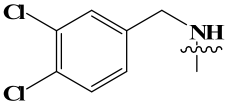	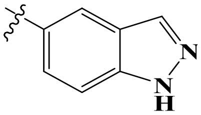	30	7
**12**	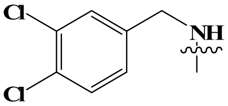	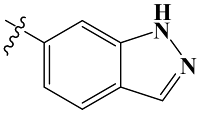	123	7
**13***	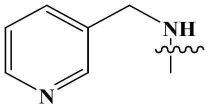	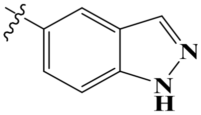	60	7

Compound	R1	R2	IC_{50} (nM)	Reference
**14**	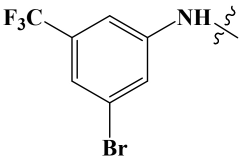	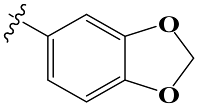	86	7
**15**	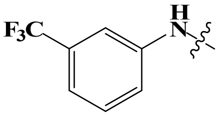	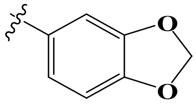	42	7
**16***	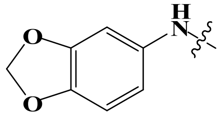	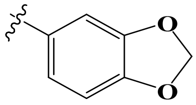	75	7
**17**	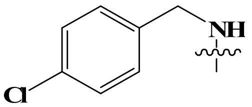	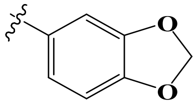	5450	7

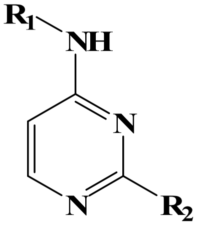

Compound	R1	R2	IC_{50} (nM)	Reference
**18**	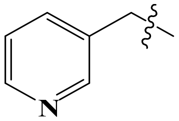	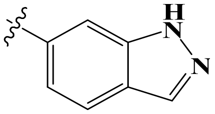	1294	7
**19***	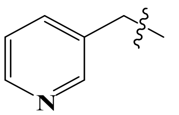	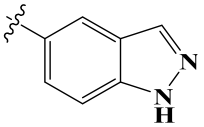	574	7
**20**	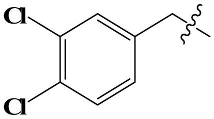	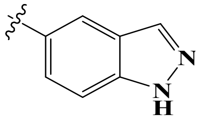	241	7
**21**	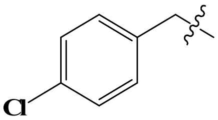	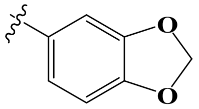	9284	7
**22**	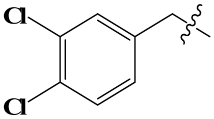	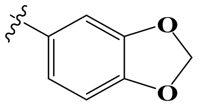	526	7
**23**	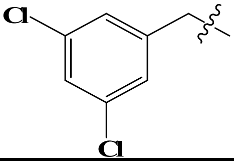	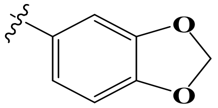	68	7

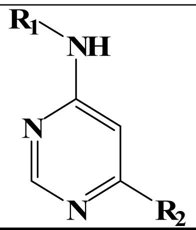

Compound	R1	R2	IC_{50} (nM)	Reference
**24**	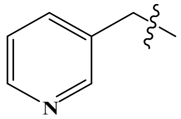	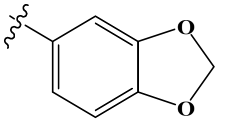	7535	7

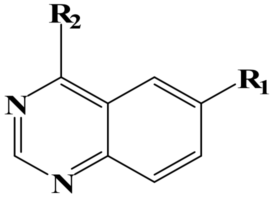

Compound	R1	R2	IC_{50} (nM)	Reference
**25**	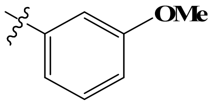	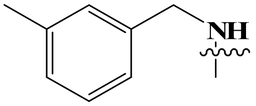	1122	8
**26**	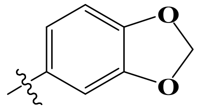	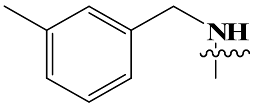	126	8
**27**	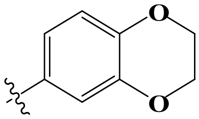	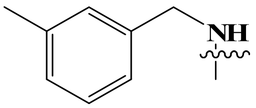	631	8
**28***	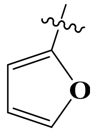	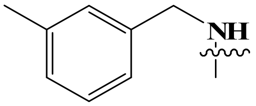	224	8
**29**	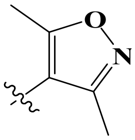	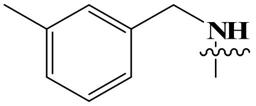	282	8
**30**	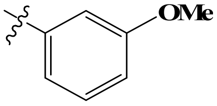	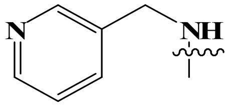	3550	8
**31**	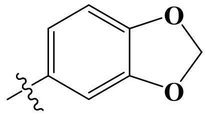	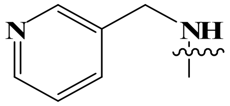	100	8
**32**	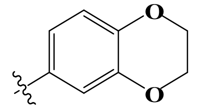	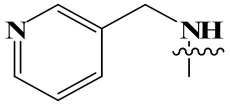	447	8

Compound	R_1	R_2	IC_{50} (nM)	Reference
**33**	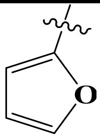	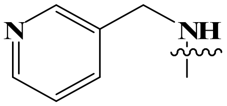	794	8
**34***	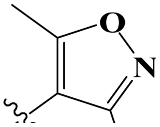	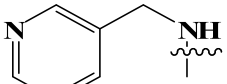	891	8
**35**	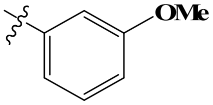	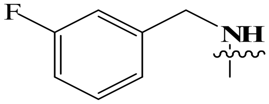	708	8
**36**	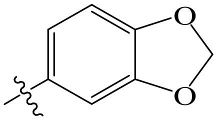	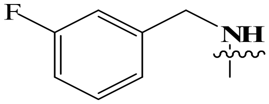	126	8
**37**	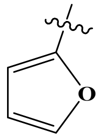	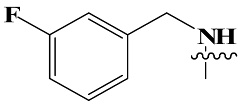	141	8
**38**	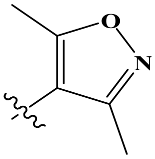	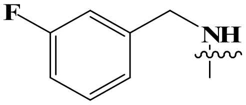	178	8
**39**	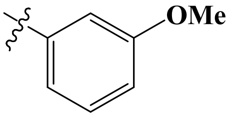	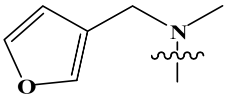	1000	8
**40**	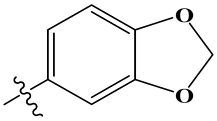	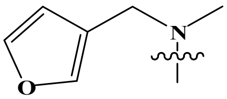	63	8
**41**	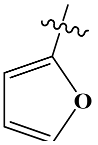	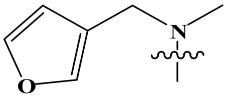	111	8
**42**	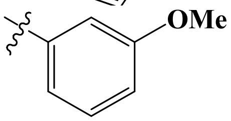	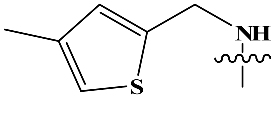	1000	8
**43**	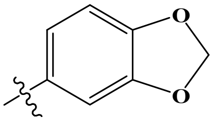	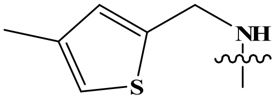	178	8

Compound	R_1	R_2	IC_{50} (nM)	Reference
**44**	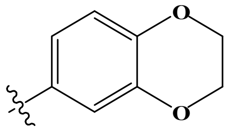	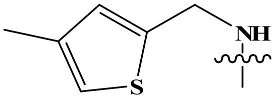	501	8
**45**	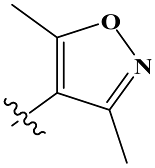	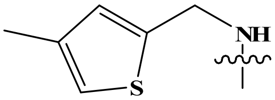	501	8
**46**	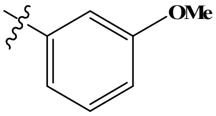	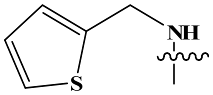	891	8
**47**	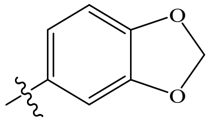	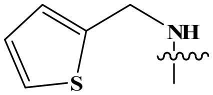	63	8
**48**	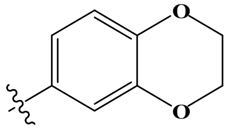	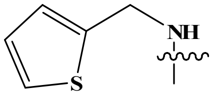	141	8

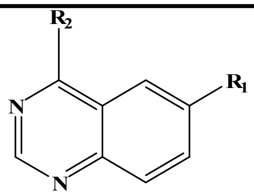

Compound	R_1	R_2	IC_{50} (nM)	Reference
**49***	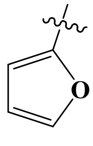	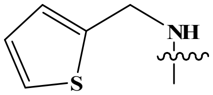	250	8
**50***	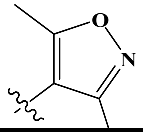	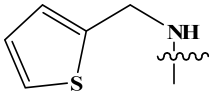	158	8

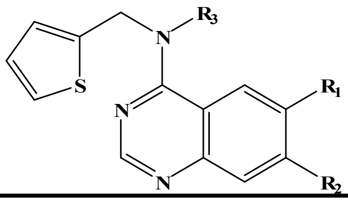

Compound	R_1	R_2	R_3	IC_{50} (nM)	Reference
**51***	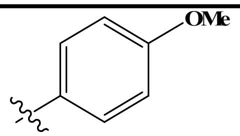	H	H	331	9
**52**	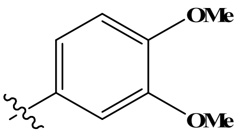	H	H	3675	9

Compound	R1	R2	R3	IC_{50} (nM)	Reference
**53***	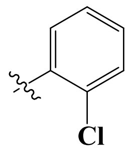	H	H	7943	9
**54***	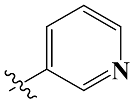	H	H	3981	9
**55**	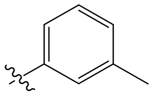	H	H	7943	9
**56***	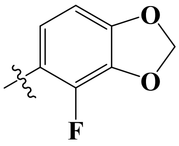	H	H	128	9
**57***	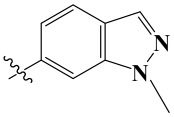	H	H	1642	9

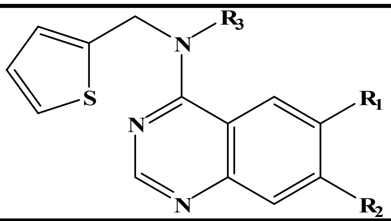

Compound	R_1	R_2	R_3	IC50 (nM)	Reference
**58**	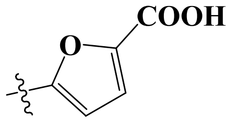	H	H	199	9
**59**	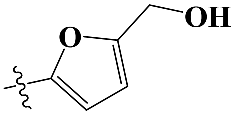	H	H	241	9

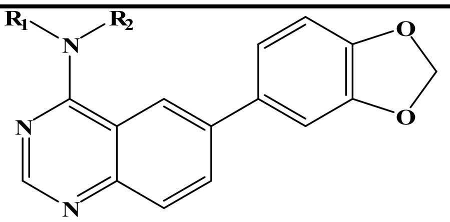

Compound	R_1	R_2	IC_{50} (nM)	Reference
**60**	CH_3	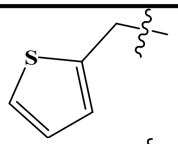	12	9
**61**	CH_2CH_3	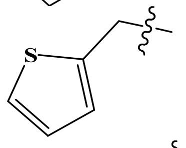	14	9
**62***	H	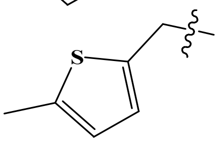	117	9

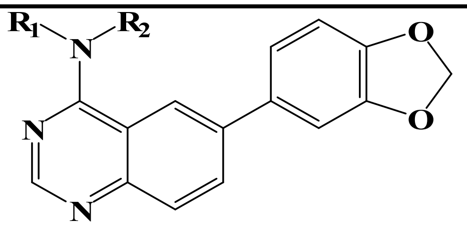

Compound	R_1	R_2	IC_{50} (nM)	Reference
**63**	CH_3	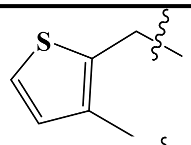	29	9
**64**	H	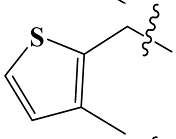	35	9
**65**	H	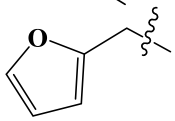	79	9
**66**	CH_3	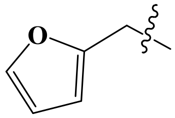	36	9
**67***	H	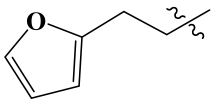	42	9
**68**	H	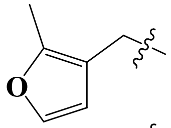	47	9
**69**	H	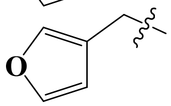	15	9
**70**	H	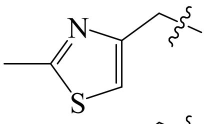	40	9
**71**	CH_3	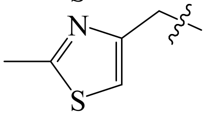	11	9
**72**	H	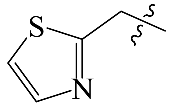	50	9
**73**	CH_3	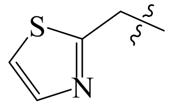	20	9
**74**	CH_3	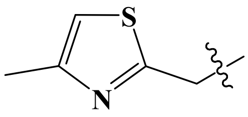	37	9
**75***	H	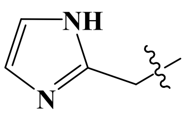	34	9
**76**	CH_3	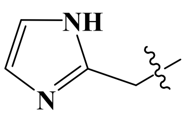	120	9

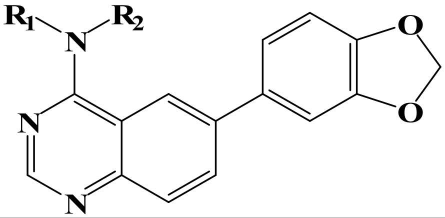

Compound	R_1	R_2	IC_{50} (nM)	Reference
**77**	H	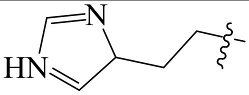	161	9
**78**	CH_3	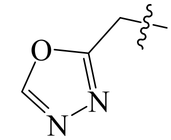	45	9
**79**	H	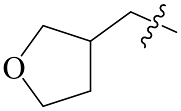	141	9
**80**	H	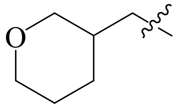	257	9
**81**	H	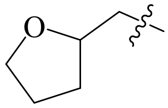	29	9
**82***	H	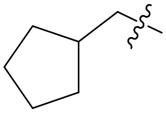	90	9
**83**	H	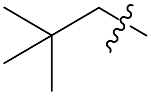	70	9

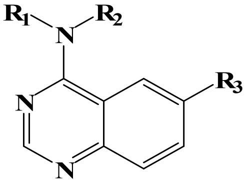

Compound	R_1	R_2	R_3	IC_{50} (nM)	Reference
**84**	H	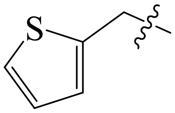	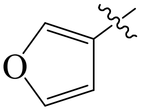	249	9
**85**	CH_3	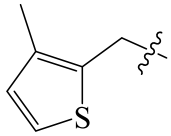	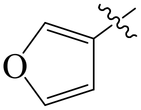	269	9
**86**	CH_3	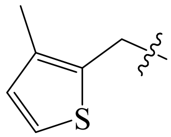	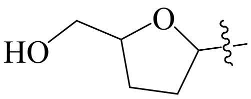	81	9

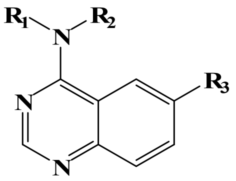

Compound	R_1	R_2	R_3	IC_{50} (nM)	Reference
**87**	H	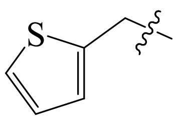	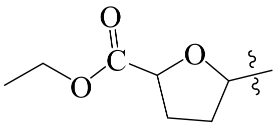	88	9
**88**	H	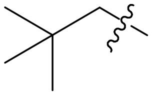	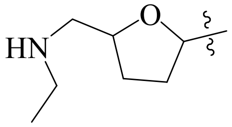	1666	9
**89**	H	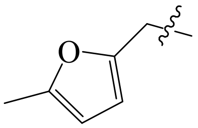	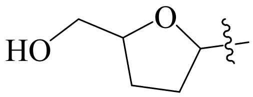	136	9
**90**	H	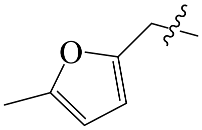	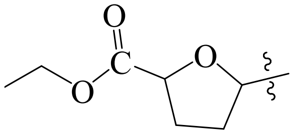	229	9
**91**	H	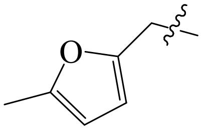	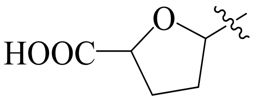	104	9

* These compounds were used as testing compounds in ML-QSAR modelling.

Table S2. Training subsets employed in exploring the pharmacophoric space of CLK4 inhibitors.

Subset	Most active [a]	Moderately active	Least active [b]	No. of compounds	Activity spread
1	**2, 10, 14, 15**	**4,12, 27, 32, 37, 44**	**21, 24, 53, 55**	**14**	**2.5**
2	**10**	**27, 32, 48, 44**	**21, 53, 55**	**8**	**2.5**
3	**60, 63, 66, 71, 73, 74, 78**	**85**	**3, 4, 17, 21, 24, 52, 53, 54, 55**	**17**	**2.5**
4	**31, 40, 43, 47, 62, 65, 67, 68, 76, 83, 85**	**-**	**4, 52, 53, 54, 55**	**16**	**2.5**
5	**26, 31, 36, 40, 47, 56, 62, 64, 65, 67, 68, 69, 70, 75, 81**	**43, 52**	**3, 17, 21, 24, 53, 55**	**23**	**2.5**
6	**11, 12, 13, 20**	**1, 18, 33**	**3, 4, 17, 21, 24, 30, 52, 53, 54, 55**	**17**	**2.0**
7	**5, 6, 7, 8, 9**	**1, 2, 12, 14, 15, 16, 22, 23, 36**	**18, 21, 52, 53, 54, 55, 57, 88**	**22**	**2.5**
8	**26, 31, 36, 40, 47, 48, 56, 62, 64, 65, 67, 68, 70, 75, 81**	**-**	**3, 17, 21, 24, 52, 53, 55**	**22**	**2.0**

a, b Potency categories [10-20].

Table S3. Training sets and HYPOGEN run parameters employed for exploring CLK4 inhibitors pharmacophoric space.

Run Number	Training set[a]	Number of compounds	Selected Features[b]	Min-Max Features[c]	Spacing[d]
1	**1**	14	HBA (1-4), HBD (0-1), Hbic (1-3), RingArom (1-3)	4-5	100
2		14	HBA (1-4), HBD (0-1), Hbic (1-3), RingArom (1-3), 10 EV	4-5	100
3		14	HBA (1-4), HBD (0-1), Hbic (1-3), RingArom (1-3)	4-5	300
4		14	HBA (1-4), HBD (0-1), Hbic (1-3), RingArom (1-3), 10 EV	4-5	300
5		14	HBA (1-4), HBD (0-1), Hbic (1-3), RingArom (1-3)	5-5	100
6		14	HBA (1-4), HBD (0-1), Hbic (1-3), RingArom (1-3), 10 EV	5-5	100
7		14	HBA (1-4), HBD (0-1), Hbic (1-3), RingArom (1-3)	5-5	300
8		14	HBA (1-4), HBD (0-1), Hbic (1-3), RingArom (1-3), 10 EV	5-5	300
1	**2**	8	HBA (1-4), HBD (0-1), Hbic (1-3), RingArom (1-3)	4-5	100
2		8	HBA (1-4), HBD (0-1), Hbic (1-3), RingArom (1-3), 10 EV	4-5	100
3		8	HBA (1-4), HBD (0-1), Hbic (1-3), RingArom (1-3)	4-5	300
4		8	HBA (1-4), HBD (0-1), Hbic (1-3), RingArom (1-3), 10 EV	4-5	300
5		8	HBA (1-4), HBD (0-1), Hbic (1-3), RingArom (1-3)	5-5	100
6		8	HBA (1-4), HBD (0-1), Hbic (1-3), RingArom (1-3), 10 EV	5-5	100
7		8	HBA (1-4), HBD (0-1), Hbic (1-3), RingArom (1-3)	5-5	300
8		8	HBA (1-4), HBD (0-1), Hbic (1-3), RingArom (1-3), 10 EV	5-5	300
1	**3**	17	HBA (1-4), Hbic (1-3), RingArom (1-3)	4-5	100
2		17	HBA (1-4), Hbic (1-3), RingArom (1-3), 10 EV	4-5	100
3		17	HBA (1-4), Hbic (1-3), RingArom (1-3)	4-5	300
4		17	HBA (1-4), Hbic (1-3), RingArom (1-3), 10 EV	4-5	300
5		17	HBA (1-4), Hbic (1-3), RingArom (1-3)	5-5	100
6		17	HBA (1-4), Hbic (1-3), RingArom (1-3), 10 EV	5-5	100
7		17	HBA (1-4), Hbic (1-3), RingArom (1-3)	5-5	300
8		17	HBA (1-4), Hbic (1-3), RingArom (1-3), 10 EV	5-5	300
1	**4**	16	HBA (1-4), HBD (0-1), Hbic (1-3), RingArom (1-3)	4-5	100
2		16	HBA (1-4), HBD (0-1), Hbic (1-3), RingArom (1-3), 10 EV	4-5	100
3		16	HBA (1-4), HBD (0-1), Hbic (1-3), RingArom (1-3)	4-5	300
4		16	HBA (1-4), HBD (0-1), Hbic (1-3), RingArom (1-3), 10 EV	4-5	300
5		16	HBA (1-4), HBD (0-1), Hbic (1-3), RingArom (1-3)	5-5	100
6		16	HBA (1-4), HBD (0-1), Hbic (1-3), RingArom (1-3), 10 EV	5-5	100
7		16	HBA (1-4), HBD (0-1), Hbic (1-3), RingArom (1-3)	5-5	300
8		16	HBA (1-4), HBD (0-1), Hbic (1-3), RingArom (1-3), 10 EV	5-5	300

[a]Training subsets as in Table S2.[b]HBA: Hydrogen Bond Acceptor, HBD: Hydrogen Bond Donor, RingArom: Ring Aromatic, Hbic: Hydrophobic. EV: maximum number of allowed exclusion spheres. Numbers in brackets refer to the allowed ranges of corresponding features in each pharmacophore modeling run.[c]Min-Max refers to the allowed range of pharmacophoric features in each model.[d]Spacing refers to the maximum interfeature distance in picometers. Other parameters were set to their default values

Table S3. Training sets and HYPOGEN run parameters employed for exploring CLK4 inhibitors pharmacophoric space.

Run Number	Training set[a]	Number of compounds	Selected Features[b]	Min-Max Features[c]	Spacing[d]
1	**5**	23	HBA (1-4), HBD (0-1), Hbic (1-3), RingArom (1-3)	4-5	100
2		23	HBA (1-4), HBD (0-1), Hbic (1-3), RingArom (1-3), 10 EV	4-5	100
3		23	HBA (1-4), HBD (0-1), Hbic (1-3), RingArom (1-3)	4-5	300
4		23	HBA (1-4), HBD (0-1), Hbic (1-3), RingArom (1-3), 10 EV	4-5	300
5		23	HBA (1-4), HBD (0-1), Hbic (1-3), RingArom (1-3)	5-5	100
6		23	HBA (1-4), HBD (0-1), Hbic (1-3), RingArom (1-3), 10 EV	5-5	100
7		23	HBA (1-4), HBD (0-1), Hbic (1-3), RingArom (1-3)	5-5	300
8		23	HBA (1-4), HBD (0-1), Hbic (1-3), RingArom (1-3), 10 EV	5-5	300
1	**6**	17	HBA (1-4), HBD (0-1), Hbic (1-3), RingArom (1-3)	4-5	100
2		17	HBA (1-4), HBD (0-1), Hbic (1-3), RingArom (1-3), 10 EV	4-5	100
3		17	HBA (1-4), HBD (0-1), Hbic (1-3), RingArom (1-3)	4-5	300
4		17	HBA (1-4), HBD (0-1), Hbic (1-3), RingArom (1-3), 10 EV	4-5	300
5		17	HBA (1-4), HBD (0-1), Hbic (1-3), RingArom (1-3)	5-5	100
6		17	HBA (1-4), HBD (0-1), Hbic (1-3), RingArom (1-3), 10 EV	5-5	100
7		17	HBA (1-4), HBD (0-1), Hbic (1-3), RingArom (1-3)	5-5	300
8		17	HBA (1-4), HBD (0-1), Hbic (1-3), RingArom (1-3), 10 EV	5-5	300
1	**7**	22	HBA (0-5), HBD (0-5), Hbic (0-5)	4-5	100
2		22	HBA (0-5), HBD (0-5), Hbic (0-5), 10 EV	4-5	100
3		22	HBA (0-5), HBD (0-5), Hbic (0-5)	4-5	300
4		22	HBA (0-5), HBD (0-5), Hbic (0-5), 10 EV	4-5	300
5		22	HBA (0-5), HBD (0-5), Hbic (0-5)	5-5	100
6		22	HBA (0-5), HBD (0-5), Hbic (0-5), 10 EV	5-5	100
7		22	HBA (0-5), HBD (0-5), Hbic (0-5)	5-5	300
8		22	HBA (0-5), HBD (0-5), Hbic (0-5), 10 EV	5-5	300
1	**8**	22	HBA (1-4), HBD (0-1), Hbic (1-3), RingArom (1-3)	4-5	100
2		22	HBA (1-4), HBD (0-1), Hbic (1-3), RingArom (1-3), 10 EV	4-5	100
3		22	HBA (1-4), HBD (0-1), Hbic (1-3), RingArom (1-3)	4-5	300
4		22	HBA (1-4), HBD (0-1), Hbic (1-3), RingArom (1-3), 10 EV	4-5	300
5		22	HBA (1-4), HBD (0-1), Hbic (1-3), RingArom (1-3)	5-5	100
6		22	HBA (1-4), HBD (0-1), Hbic (1-3), RingArom (1-3), 10 EV	5-5	100
7		22	HBA (1-4), HBD (0-1), Hbic (1-3), RingArom (1-3)	5-5	300
8		22	HBA (1-4), HBD (0-1), Hbic (1-3), RingArom (1-3), 10 EV	5-5	300

[a]Training subsets as in Table S2. [b]HBA: Hydrogen Bond Acceptor, HBD: Hydrogen Bond Donor, RingArom: Ring Aromatic, Hbic: Hydrophobic. EV: maximum number of allowed exclusion spheres. Numbers in brackets refer to the allowed ranges of corresponding features in each pharmacophore modeling run.[c]Min-Max refers to the allowed range of pharmacophoric features in each model.[d]Spacing refers to the maximum interfeature distance in picometers. Other parameters were set to their default values.

Table S4. The performance of the best representatives (cluster centres) of clustered pharmacophore hypothesis generated for CLK4 inhibitors.

Training Subset	Run[a]	Hypo thesis[b]	Pharmacophoric Features in Generated Hypothesis	Total cost	Cost of null hypothesis	Residual Cost[c]	R_{run}[d]	F_{total}[e]	Cat-Scramble
**1**	2	9	HBA, Hbic, 2×RingArom, EV	68.5	73.7	5.2	0.94	30.9	90%
	4	5	HBA, Hbic, 2×RingArom, EV	67.3	73.7	6.4	0.92	4.35	90%
	2	10	HBA, 2×Hbic, RingArom	68.6	73.7	5.1	0.94	41.4	90%
	5	10	HBA, 3×Hbic, RingArom	68.1	73.7	5.6	0.89	20.0	90%
	5	1	HBA, 3×Hbic, RingArom	65.5	73.7	8.2	0.95	2.3	90%
	7	1	HBA, 3×Hbic, RingArom	65.1	73.7	8.6	0.91	0.58	80%
	6	2	HBA, 3×Hbic, RingArom	65.4	73.7	8.3	0.95	4.8	90%
	3	2	HBA, 2×Hbic, RingArom	66.0	73.7	7.7	0.95	1.88	90%
	3	6	HBA, 2×Hbic, RingArom	68.6	73.7	5.1	0.91	0.77	90%
	4	7	HBA, 2×Hbic, RingArom, 3×EV	68.0	73.7	5.7	0.90	0.59	90%
	1	8	HBA, HBD, Hbic, RingArom	67.8	73.7	5.9	0.95	1.48	90%
**2**	2	7	HBA, Hbic, 2×RingArom, 5×EV	52.8	53.5	0.7	0.97	8.98	90%
	2	8	2×HBA, Hbic, RingArom, 2×EV	52.9	53.5	0.6	0.97	6.8	90%
	7	8	HBA, 3×Hbic, RingArom	49.5	53.5	4	0.95	5.9	90%
	8	9	HBA, 3×Hbic, RingArom	49.2	53.5	4.3	0.94	3.8	90%
	8	5	HBA, 3×Hbic, RingArom	49.0	53.5	4.5	0.97	2.2	90%
	5	2	HBA, 3×Hbic, RingArom	50.5	53.5	3.0	0.97	1.4	90%
	5	3	HBA, 3×Hbic, RingArom	50.8	53.5	2.7	0.96	0.09	90%
	5	5	HBA, 3×Hbic, RingArom	50.9	53.5	2.6	0.97	5.27	90%
	1	5	2×HBA, Hbic, RingArom	52.6	53.5	0.9	0.98	32.1	90%
	1	7	2×HBA, Hbic, RingArom	52.7	53.5	0.8	0.98	2.55	90%
3	3	7	HBA, Hbic, 2×RingArom	72.1	127.3	55.2	0.99	14.9	90%
	3	10	HBA, Hbic, 2×RingArom	72.3	127.3	55.0	0.99	3.3	90%
	4	4	HBA, Hbic, 2×RingArom, EV	71.9	127.3	55.4	0.99	9.8	90%
	1	2	HBA, Hbic, 2×RingArom	73.9	127.3	53.4	0.99	20.1	90%
	1	3	2×HBA, Hbic, RingArom	74.0	127.3	53.3	0.99	7.8	90%
	1	7	2×HBA, Hbic, RingArom	74.5	127.3	52.8	0.98	13.0	90%
	8	3	HBA, 3×Hbic, RingArom, 2×EV	69.8	127.3	57.5	0.98	10	90%
	8	4	HBA, 3×Hbic, RingArom	70	127.3	57.3	0.99	18.3	90%
	6	7	2×HBA, Hbic, RingArom, 2×EV	74.4	127.3	52.9	0.98	7.89	90%

Table S4. The performance of the best representatives (cluster centres) of clustered pharmacophore hypothesis generated for CLK4 inhibitors.

Training Subset	Run[a]	Hypo thesis[b]	Pharmacophoric Features in Generated Hypothesis	Total cost	Cost of null hypothesis	Residual Cost[c]	R_{run}[d]	F_{total}[e]	Cat-Scramble
	6	8	HBA, Hbic, 2×RingArom	74.4	127.3	52.9	0.99	15.15	90%
**4**	3	4	HBA, 2×Hbic, RingArom	72.1	78.7	6.6	0.97	4.75	90%
	3	6	2×HBA, Hbic, RingArom	73.6	78.7	5.1	0.91	14.3	90%
	2	1	HBA, 2×Hbic, RingArom	73.9	78.7	4.8	0.97	17.5	90%
	2	2	HBA, 2×Hbic, RingArom	74.7	78.7	4.0	0.95	12.9	90%
	2	3	2×HBA, Hbic, RingArom	74.8	78.7	3.9	0.95	16.29	90%
	8	8	HBA, 3×Hbic, RingArom	64.5	78.7	14.2	0.78	10.66	90%
	5	10	HBA, 3×Hbic, RingArom	72.8	78.7	5.9	0.91	5.15	90%
	5	7	HBA, 3×Hbic, RingArom	72.6	78.7	6.1	0.91	25.5	90%
	5	1	HBA, 3×Hbic, RingArom	71.8	78.7	6.9	0.93	15.4	90%
	1	2	HBA, 2×Hbic, RingArom	74.7	78.7	4.0	0.95	19.0	90%
	1	10	2×HBA, Hbic, RingArom	75.4	78.7	3.3	0.93	12.0	90%
	4	10	2×HBA, Hbic, RingArom	73.8	78.7	4.9	0.90	1.1	90%
**5**	1	6	HBA, Hbic, 2×RingArom	100.3	127.7	27.4	0.96	15.6	90%
	2	7	HBA, Hbic, 2×RingArom	99.8	127.7	27.9	0.97	13.1	90%
	**2**[f]	**8**	**HBA, 2×Hbic, RingArom, 3× EV**	**99.8**	**127.7**	**27.9**	**0.97**	**23.4**	**90%**
	8	1	HBA, 3× Hbic, RingArom, EV	90.7	127.7	37.0	0.97	2.1	90%
	8	2	HBA, 3× Hbic, RingArom, EV	93.1	127.7	34.6	0.95	1.7	90%
	7	7	HBA, 3× Hbic, RingArom	100.9	127.7	26.8	0.86	0.76	90%
	6	8	HBA, 3× Hbic, RingArom, 3× EV	98.3	127.7	29.4	0.94	29.2	90%
	6	3	HBA, 3× Hbic, RingArom, 4× EV	97.4	127.7	30.3	0.95	14.8	90%
	6	7	HBA, 3× Hbic, RingArom,EV	98.2	127.7	29.5	0.94	0.19	90%
	4	2	2×HBA, Hbic, RingArom	92.9	127.7	34.8	0.97	5.7	90%
	1	7	2×HBA, Hbic, RingArom	100.4	127.7	27.3	0.96	14	90%
	1	1	2×HBA, Hbic, RingArom	99.8	127.7	27.9	0.97	28.5	90%
**6**	4	3	HBA, Hbic, 2×RingArom, 2× EV	75.6	80.8	5.2	0.96	13.8	90%
	4	8	HBA,Hbic, 2× RingArom	76.4	80.8	4.4	0.94	2.6	90%
	1	1	HBA,Hbic, 2× RingArom	77.6	80.8	3.2	0.94	0.06	80%
	8	5	HBA,Hbic, 2×RingArom, 2× EV	76.6	80.8	4.2	0.87	1.7	90%
	4	10	2×HBA, 3× Hbic, RingArom, 4× EV	76.5	80.8	4.3	0.94	12.8	90%
	3	1	2×HBA, Hbic, RingArom	75.7	80.8	5.1	0.95	1.1	90%
	**2**[f]	**7**	**HBA, HBD, Hbic, RingArom, 4× EV**	**78.0**	**80.8**	**2.8**	**0.93**	**4.9**	**90%**
	1	9	HBA, HBD, Hbic, RingArom	78.8	80.8	2.0	0.91	0.01	90%
	2	8	HBA, HBD, Hbic, RingArom, EV	78.0	80.8	2.8	0.93	1.5	90%

Table S4. The performance of the best representatives (cluster centres) of clustered pharmacophore hypothesis generated for CLK4 inhibitors.

Training Subset	Run[a]	Hypo thesis[b]	Pharmacophoric Features in Generated Hypothesis	Total cost	Cost of null hypothesis	Residual Cost[c]	R_{run}[d]	F_{total}[e]	Cat-Scramble
	2	3	HBA, HBD, Hbic, RingArom, EV	77.1	80.8	3.7	0.95	3.8	90%
	4	6	2×HBA, 2×Hbic	97.9	124.8	26.9	0.91	8.3	90%
**7**	4	5	2×HBA, 2×Hbic	98.7	124.8	26.1	0.89	2.7	90%
	4	8	2×HBA, 2×Hbic, EV	99.2	124.8	25.6	0.89	7.8	90%
	3	9	2×HBA, 3×Hbic	100.1	124.8	24.7	0.88	9.2	90%
	8	5	HBA, HBD, 3×Hbic, 2×EV	92.6	124.8	32.2	0.90	6.6	90%
	7	3	HBA, HBD, 3×Hbic	91.8	124.8	33.0	0.92	1.4	90%
	7	10	HBA, HBD, 3×Hbic	95.7	124.8	29.1	0.87	0.48	90%
	6	5	HBA, HBD, 3×Hbic, 3×EV	97.9	124.8	26.9	0.92	6.1	90%
	6	2	HBA, HBD, 3×Hbic, 2×EV	96.3	124.8	28.5	0.93	3.1	90%
	1	6	HBA, HBD, 3×Hbic	99.9	124.8	24.9	0.91	1.3	90%
	5	8	HBA, HBD, 3×Hbic	98.3	124.8	26.5	0.91	0.96	90%
	3	10	HBA, HBD, 2×Hbic	100.5	124.8	24.3	0.87	0.07	90%
	4	7	HBA, HBD, 2×Hbic, 2×EV	99.1	124.8	25.7	0.89	0.56	90%
	4	10	HBA, HBD, 2×Hbic,EV	99.4	124.8	25.4	0.89	0.01	90%
	2	2	2×HBA, HBD, Hbic, EV	97.3	124.8	27.5	0.94	22.5	90%
	1	10	2×HBA, HBD, Hbic	100.6	124.8	24.2	0.89	1.0	90%
**8**	1	7	HBA, Hbic, 2×RingArom	93.6	117.7	26.1	0.98	15.6	90%
	2	8	HBA, Hbic, 2× RingArom	93.6	117.7	24.1	0.98	14.9	90%
	3	3	HBA, 2×Hbic, RingArom	90.8	117.7	26.9	0.98	3.6	90%
	2	4	HBA, 2×Hbic, RingArom	93.3	117.7	24.4	0.98	3.6	90%
	6	3	HBA, 3×Hbic, RingArom	90.3	117.7	27.4	0.95	10.8	90%
	6	9	HBA, 3×Hbic, RingArom	94.1	117.7	23.6	0.90	4.5	90%
	5	9	HBA, 3×Hbic, RingArom	94.6	117.7	23.1	0.92	11.2	90%
	3	1	2×HBA, Hbic, RingArom	90.5	117.7	27.2	0.98	5.4	90%
	**3**[f]	**8**	**HBA, HBD, Hbic, RingArom**	**91.3**	**117.7**	**26.4**	**0.97**	**1.0**	**90%**

[a]Correspond to runs in Table S3.
[b]Best models from their respective clusters, as judged based on F-statistic generated by correlating fit values of the whole list of collected compounds with the corresponding bioactivities (table S1). Numbers correspond to rank of each hypothesis in each particular CATALYST automatic pharmacophore generating run.
[c]The difference between the total cost and the cost of the corresponding null hypothesis.
[d]The correlation coefficients between bioactivity estimates and bioactivities of corresponding training compounds in the particular subset.
[e]Fisher statistic calculated based on the linear regression between the fit values of collected inhibitors (**1-91**, table S1) against pharmacophore hypothesis (employing the "best fit" option) and their respective CLK4 inhibitors bioactivities.
[f]Bolded models represent the criteria of pharmacophores that emerged in the in the successful ML-QSAR models.

Table S5. The chemical structures of the ROC-testing set in SMILES format and their corresponding bioactivities.

ChEMBL code	Smiles	IC_{50} or Ki	Activity Class
CHEMBL1980407	[H]n5c(C([H])=C4c3c(c([H])c([H])c(c2c([H])nc([H])c(OC([H])([H])[C@]([H])(N([H])[H])C([H])([H])c1c([H])c([H])c([H])c([H])c1[H])c2[H])c3[H])N([H])C4=O)c([H])c([H])c5[H]	0.3162	Active
CHEMBL1965660	O=C(N([H])[H])c2c([H])c(c1nc(nc([H])c1[H])N([H])[H])n([H])c2c3c([H])c([H])c([Cl])c([H])c3[Cl]	0.631	Active
CHEMBL1965660	O=C(N([H])[H])c2c([H])c(c1nc(nc([H])c1[H])N([H])[H])n([H])c2c3c([H])c([H])c([Cl])c([H])c3[Cl]	0.631	Active
CHEMBL2007574	[Cl]c1nc(nc(c1[H])c3c([H])n([H])c2nc([H])c([H])c([H])c23)N([H])[H]	0.631	Active
CHEMBL1973720	[Cl]c1nc(nc(c1[H])c3c([H])n([H])c2nc([H])c([H])c([H])c23)N([H])[C@@]4([H])C([H])([H])C([H])([H])[C@@]([H])(N([H])[H])C([H])([H])C4([H])[H]	1	Active
CHEMBL1977138	N#Cc4c([H])c([H])c3nc([H])n(c2nc(N([H])[C@]([H])(c1c([H])c([H])c([H])c([H])c1[F])C([H])([H])[H])c([H])nc2[H])c3c4[H]	1	Active
CHEMBL1973720	[Cl]c1nc(nc(c1[H])c3c([H])n([H])c2nc([H])c([H])c([H])c23)N([H])[C@@]4([H])C([H])([H])C([H])([H])[C@@]([H])(N([H])[H])C([H])([H])C4([H])[H]	1	Active
CHEMBL1981133	[H]n1c([H])c(c2c1c([H])c([H])c([H])c2[H])C([H])([H])[C@]([H])(N([H])[H])C([H])([H])Oc5c([H])nc([H])c(c4c([H])c([H])c3c([H])nc([H])c([H])c3c4[H])c5[H]	1	Active
CHEMBL1990162	N#Cc5c([H])c([H])c(C(=O)N([H])c4nn([H])c3c([H])c([H])c(c1nnn(c1[H])C([H])([H])c2c([H])c([H])c([H])c([H])c2[H])c([H])c34)c([H])c5[H]	1.259	Active
[illegible]MBL1996980	O=C(N([H])[C@]4([H])C([H])([H])C([H])([H])[C@]([H])(N([H])c1nc([Cl])c([H])c(n1)c3c([H])n([H])c2nc([H])c([H])c([H])c23)C([H])([H])C4([H])[H])C([H])([H])N([H])C([H])([H])[H]	1.259	Active
CHEMBL1987448	[H]n2c([H])c(c1c([H])c(nc([Cl])c1[H])N([H])[H])c3c2nc([H])c([H])c3[H]	1.585	Active
CHEMBL1983575	O=C(N([H])N=C([H])c1c([H])c([Br])c(O[H])c([Br])c1[H])[illegible])c2c([H])c([H])c([H])c([H])c2[H]	1.585	Active
CHEMBL1967252	[H]n1nc(c3c1c([H])c([H])c(c2c([H])c([H])c([H])nc2[H])c3[H])C([H])([H])[H]	1.995	Active
CHEMBL1980562	O=[S](=O)(N([H])[C@@]4([H])C([H])([H])C([H])([H])[C@]([H])(N([H])c1nc([Cl])c([H])c(n1)c3c([H])n([H])c2nc([H])c([H])c([H])c23)C([H])([H])C4([H])[H])C([H])([H])[H]	1.995	Active
CHEMBL1982271	O=C(N([H])[C@]4([H])C([H])([H])C([H])([H])[C@]([H])(N([H])c1nc([Cl])c([H])c(n1)c3c([H])n([H])c2nc([H])c([H])c([H])c23)C([H])([H])C4([H])[H])C([H])([H])N(C([H])([H])[H])C([H])([H])[H]	1.995	Active
CHEMBL1980995	O=C8c2c(c1c7c(n5c1c4c2c3c([H])c([H])c([H])c([H])c3n4[C@@]6([H])O[C@]5(C([H])([H])[H])[C@@]([H])(OC([H])([H])[H])[C@@]([H])(N([H])C([H])([H])[H])C6([H])[H])c([H])c([H])c([H])c7[H])C([H])([H])N8[H]	1.995	Active
CHEMBL2002613	O=C(c1c([H])c(OC([H])([H])[H])c([H])c(OC([H])([H])[H])c1[H])N([H])c5nn([H])c4c([H])c([H])c(c2nnn(c2[H])C([H])([H])c3c([H])c([H])c([H])c([H])c3[H])c([H])c45	2.512	Active
CHEMBL1969151	[Cl]c1nc(nc(c1[H])c3c([H])n([H])c2nc([H])c([H])c([H])c23)N([H])[C@@]4([H])C([H])([H])C([H])([H])[C@@]([H])(N(C([H])([H])[H])C([H])([H])[H])C([H])([H])C4([H])[H]	2.512	Active

CHEMBL1974935	[H]n1c([H])c(c2c1c([H])c([H])c([H])c2[H])C([H])([H])[C@@]([H])(N([H])[H])C([H])([H])Oc4c([H])nc([H])c(C([H])=C([H])c3c([H])c([H])nc([H])c3[H])c4[H]	3.162	Active
CHEMBL1990912	[H]OC([H])([H])C([H])([H])N([H])c1nc([H])c3c(c1[H])c([H])c(c2c([H])c([H])c([H])nc2[H])c([H])c3[H]	3.162	Active
CHEMBL1999279	[H]n3c([H])c(c1nc(nc([H])c1[H])N([H])C(c2c([H])c([H])c([H])c([H])c2[H])(C([H])([H])[H])C([H])([H])[H])c4c3nc([H])c([H])c4[H]	3.162	Active
CHEMBL1987948	O=[S](=O)(N([H])[C@]4([H])C([H])([H])C([H])([H])C([H])([H])[C@]([H])(N([H])c1nc([Cl])c([H])c(n1)c3c([H])n([H])c2nc([H])c([H])c([H])c23)C4([H])[H])C([H])([H])[H]	3.162	Active
CHEMBL1971951	O=C(N([H])[H])c2c([H])c([H])c(c1nc(nc([H])c1[H])N([H])[H])c([H])c2N3C([H])([H])C([H])([H])C([H])([H])C([H])([H])C3([H])[H]	3.162	Active
CHEMBL1999484	[H]C([H])([H])N([H])c1nc(c([s]1)c2nc(nc([H])c2[H])N([H])c4c([H])c([H])c(N3C([H])([H])C([H])([H])N([H])C([H])([H])C3([H])[H])c([H])c4[H])C([H])([H])[H]	3.162	Active
CHEMBL1983157	[H]n3nc(N([H])[H])c2c(N([H])C([H])([H])C([H])([H])C([H])([H])C([H])([H])[H])c([H])c(c1c([H])c([H])nc([H])c1[H])c([H])c23	3.162	Active
CHEMBL1977128	N#Cc5c([H])c([H])c([H])c(C(=O)N([H])c4nn([H])c3c([H])c([H])c(c1nnn(c1[H])C([H])([H])c2c([H])c([H])c([H])c([H])c2[H])c([H])c34)c5[H]	3.981	Active
CHEMBL2005718	O=[S](=O)(N([H])C([H])([H])C([H])([H])N([H])c1nc([Cl])c([H])c(c1[H])c3c([H])n([H])c2nc([H])c([H])c([H])c23)C([H])([H])[H]	3.981	Active
CHEMBL1375640	[Cl]c3c(OC([H])([H])c1nc(c([s]1)c2nc(nc([H])c2[H])N([H])[H])C([H])([H])[H])c([H])c([H])c([H])c3[H]	3.981	Active
CHEMBL1997643	O=C(c1c(OC([H])([H])[H])c([H])c([H])c([H])c1[H])N([H])c5nn([H])c4c([H])c([H])c(c2nnn(c2[H])C([H])([H])c3c([H])c([H])c([H])c([H])c3[H])c([H])c45	3.981	Active
CHEMBL225519	O=C2N([H])C([H])([H])C([H])([H])c3n([H])c(c1c([H])c([H])nc([H])c1[H])c([H])c23	3.981	Active
CHEMBL1996339	[H]n2c([H])c(c1c([H])c(nc([Cl])c1[H])N([H])C([H])([H])C([H])=C([H])[H])c3c2nc([H])c([H])c3[H]	3.981	Active
CHEMBL1982400	[H]n2nc(c1c([H])c([H])c([H])c([H])c1[H])c5c2c([H])c([H])c(c3nnn(c3[H])C([H])([H])c4c([H])c([H])c([H])c([H])c4[H])c5[H]	3.981	Active
CHEMBL1997924	[H]n4nc(N([H])c1nc(nc2c1[s]c([H])c2[H])N([H])C([H])([H])c3c([H])c([H])c([H])c([H])c3[H])c([H])c4C5([H])C([H])([H])C5([H])[H]	3.981	Active
CHEMBL1682554	[H]n1nc([H])c4c1c([H])c([H])c(c3nc2nc([H])c([H])c([H])n2c3N([H])C([H])([H])C([H])([H])C([H])([H])C([H])([H])[H])c4[H]	3.981	Active
CHEMBL1969473	[H]n1nc([H])c5c1c([H])c([H])c(c2nnn(c2c3c([H])c([H])c([H])c([H])c3[H])C([H])([H])c4c([H])c([H])c([H])c([H])c4[H])c5[H]	5.012	Active
CHEMBL1988163	O=[S](=O)(N([H])[H])c4c([H])c([H])c([H])c(N([H])c3nc([H])c2c([H])c([H])n(c1c([H])c([H])c([H])c([H])c1[H])c2n3)c4[H]	5.012	Active
CHEMBL1998121	O=C(N([H])c2c([H])c([H])c1n([H])nc([H])c1c2[H])[C@@]([H])(c3c([H])c([H])c([Cl])c([Cl])c3[H])C([H])([H])C([H])([H])N([H])[H]	5.012	Active
CHEMBL1988331	[H]n3c([H])c(c1c([H])c(nc([Cl])c1[H])N([H])C([H])([H])c2c([H])c([H])c([H])c(c2C([H])([H])[H])C([H])([H])[H])c4c3nc([H])c([H])c4[H]	5.012	Active
CHEMBL1964804	O=[S](=O)(N([H])C([H])([H])C([H])([H])Oc3c([H])nc([H])c(c2c([H])c([H])c1c([H])nc([H])c([H])c1c2[H])c3[H])C([H])([H])[H]	5.012	Active

CHEMBL1974250	O=C(N([H])C([H])([H])c1c([H])c([H])c([H])c([H])c1[H])c3c([H])n([H])n2c(=O)c([H])c(nc23)c4c([H])c([H])nc([H])c4[H]	5.012	Active
CHEMBL1985723	[H]n2c([H])c(c1c([H])c(nc([Cl])c1[H])N([H])C([H])([H])C(C([H])([H])[H])(C([H])([H])[H])C([H])([H])N([H])[H])c3c2nc([H])c([H])c3[H]	5.012	Active
CHEMBL1979773	O=C(N(C([H])([H])[H])C([H])([H])C([H])([H])N([H])[H])c4c([H])c([H])c([H])c(n1nc(c([H])c1N([H])C(=O)N([H])c3c([H])c([H])c([H])c2c([H])c([H])c([H])c([H])c23)C(C([H])([H])[H])(C([H])([H])[H])C([H])([H])[H])c4[H]	5.012	Active
CHEMBL1971172	[H]n2c([H])c(c1c([H])c(nc([Cl])c1[H])N([H])C([H])([H])C(C([H])([H])[H])(C([H])([H])[H])C([H])([H])N([H])[S](=O)(=O)C([H])([H])[H])c3c2nc([H])c([H])c3[H]	5.012	Active
CHEMBL2005387	[H]n2c([H])c(c1c([H])c(nc([Cl])c1[H])N([H])C([H])([H])C(O[H])(C([H])([H])[H])C([H])([H])[H])c3c2nc([H])c([H])c3[H]	5.012	Active
CHEMBL2002346	[H]n3c([H])c(c2nc([H])nc(N([H])C([H])([H])c1c([H])c([H])c([H])c([H])c1[H])c2[H])c4c3nc([H])c([H])c4[H]	5.012	Active
CHEMBL1987679	[H]n5c4c([H])c([H])c(c3c([H])nc([H])c(OC([H])([H])[C@@]([H])(N([H])[H])C([H])([H])c2c([H])n([H])c1c([H])c([H])c([H])c([H])c12)c3[H])c([H])c4oc5=O	5.012	Active
CHEMBL1972290	[H]n2c([H])c(c1c([H])c(nc([Cl])c1[H])N([H])C([H])([H])C([H])([H])N([H])[H])c3c2nc([H])c([H])c3[H]	5.012	Active
CHEMBL1983932	O=C(N([H])c2c1c([H])c([H])c(OC([H])([H])[H])c([H])c1nc([H])c2[H])N([H])c3nc([Br])c([H])c([H])c3[H]	5.012	Active
CHEMBL1998068	[H]n2c([H])c(c1c([H])c(nc([Cl])c1[H])N([H])[C@]([H])(C([H])([H])OC([H])([H])[H])C([H])([H])[H])c3c2nc([H])c([H])c3[H]	6.31	Active
CHEMBL1968930	[Cl]c1nc(nc(c1[H])c3c([H])n([H])c2nc([H])c([H])c([H])c23)N([H])C([H])([H])C(C([H])([H])[H])(C([H])([H])[H])C([H])([H])N([H])[H]	6.31	Active
CHEMBL1997129	O=C3N([H])C([H])([H])C([H])([H])c4n([H])c(c2c([H])c([H])nc(C([H])=C([H])c1c([H])c([H])c([H])c([H])c1[H])c2[H])c([H])c34	6.31	Active
CHEMBL1993877	[H]C#Cc2c([H])n(c1nc([H])nc(N([H])[H])c12)C3([H])C([H])([H])C([H])([H])C([H])([H])C3([H])[H]	6.31	Active
CHEMBL1986499	[H]n1nc(N([H])[H])c5c1c([H])c([H])c(c2nnn(c2c3c([H])c([H])c([F])c([H])c3[H])C([H])([H])c4c([H])c([H])c([H])c([H])c4[H])c5[H]	6.31	Active
CHEMBL2005936	O=C(N([H])c5nn([H])c4c([H])c([H])c(c1nnn(c1c2c([H])c([H])c([H])c([H])c2[H])C([H])([H])c3c([H])c([H])c([H])c([H])c3[H])c([H])c45)C([H])([H])C([H])([H])C([H])([H])[H]	6.31	Active
CHEMBL1995813	[H]n3c([H])c(c1nc(nc([H])c1[H])N([H])[C@@]2([H])[C@@]([H])(N([H])[H])C([H])([H])C([H])([H])C([H])([H])C2([H])[H])c4c3nc([H])c([H])c4[H]	6.31	Active
CHEMBL1995712	O=C(N([H])[C@]4([H])C([H])([H])C([H])([H])C([H])([H])[C@]([H])(N([H])c1nc([Cl])c([H])c(n1)c3c([H])n([H])c2nc([H])c([H])c([H])c23)C4([H])[H])C([H])([H])N(C([H])([H])[H])C([H])([H])[H]	6.31	Active
CHEMBL1995932	[H]n3c([H])c(c2nc([H])nc(N([H])C([H])([H])c1c([H])c([H])c([H])c(c1[H])C([H])([H])[H])c2[H])c4c3nc([H])c([H])c4[H]	6.31	Active
CHEMBL1973540	O=C3N([H])c2c([H])c([H])c1nc([H])[s]c1c2/C3=C(/[H])N([H])c4c([H])c([H])c(c([H])c4[H])[S](=O)(=O)N([H])c5nc([H])c([H])c([H])c5[H]	6.31	Active
CHEMBL1972158	[H]n1nc(N([H])[H])c5c1c([H])c([H])c(c2nnn(c2c3c([H])c([H])c([H])c([H])c3[H])C([H])([H])c4c([H])c([H])c([H])c([H])c4[H])c5[H]	7.943	Active
CHEMBL1983595	O=C(N([H])c4nn([H])c3c([H])c([H])c(c1nnn(c1[H])C([H])([H])c2c([H])c([H])c([H])c([H])c2[H])c([H])c34)c5c([H])c([H])c([H])c([Cl])c5[H]	7.943	Active

CHEMBL1988581	O=c1c([H])c(nc2n1c([H])c([H])c(c2O[H])C([H])([H])N(C([H])([H])[H])C([H])([H])[H])C([H])([H])[H]	7.943	Active
CHEMBL1992922	[H]n2c1nc([H])nc(N([H])C([H])(C([H])([H])O[H])C([H])([H])O[H])c1c(c2[H])c3c([H])c([H])c([H])c([H])c3[H]	7.943	Active
CHEMBL1983315	[H]n1nc(c5c1c([H])c([H])c(c2nnn(c2c3c(c([H])c([H])c([H])c3[H])C([H])([H])[H])C([H])([H])c4c([H])c([H])c([H])c([H])c4[H])c5[H])C([H])([H])[H]	7.943	Active
CHEMBL1998470	O=C(N([H])c1nc([Cl])c([H])c(c1[H])c3c([H])n([H])c2nc([H])c([H])c([H])c23)C([H])([H])C([H])([H])C([H])([H])[H]	7.943	Active
CHEMBL1975440	O=C(N([H])[H])c4c([H])c([H])c(N([H])c3nc([H])nc2n([H])c([H])c(c1c([H])c([H])c([H])c([H])c1[H])c23)c([H])c4[H]	10	Active
CHEMBL226232	O=C2N([H])C([H])([H])C([H])([H])c3n([H])c(c1c([H])c([H])nc([Cl])c1[H])c([H])c23	10	Active
CHEMBL1996447	[H]n3nc(N([H])[H])c2c(OC([H])([H])C([H])([H])C([H])([H])C([H])([H])[H])c([H])c(c1c([H])c([H])nc([H])c1[H])c([H])c23	10	Active
CHEMBL1985654	[H]n2c([H])c(c1c([H])c(nc([Cl])c1[H])N(C([H])([H])C([H])([H])OC([H])([H])[H])C([H])([H])C([H])([H])OC([H])([H])[H])c3c2nc([H])c([H])c3[H]	10	Active
CHEMBL1991410	O=C(N([H])c1nc([Cl])c([H])c(c1[H])c3c([H])n([H])c2nc([H])c([H])c([H])c23)c4c([F])c([H])c([H])c([H])c4[F]	10	Active
CHEMBL1983855	[H]n1nc(c3c1c([H])c([H])c(c2c([H])nc([H])c(OC([H])([H])C([H])([H])N([H])C([H])([H])[H])c2[H])c3[H])C([H])([H])[H]	10	Active
CHEMBL2003637	O=[N+]([O-])c4c([H])c([H])c([H])c(c2nnc1c([H])c([H])c(nn12)N3C([H])([H])C([H])([H])C([H])([H])C([H])([H])C3([H])[H])c4[H]	10	Active
CHEMBL1977223	O=C(N([H])c4nn([H])c3c([H])c([H])c(c1nnn(c1[H])C([H])([H])c2c([H])c([H])c([H])c([H])c2[H])c([H])c34)C([H])([H])C([H])([H])C([H])([H])[H]	10	Active
CHEMBL1190711	O=C(c1c(nc([H])c([H])c1[H])N([H])c3nc(c2c([H])c([H])c(OC([H])([H])[H])c(OC([H])([H])[H])c2[H])c([H])c4nc([H])c([H])n34)N([H])[H]	10	Active
CHEMBL1682552	[H]n1nc([H])c5c1c([H])c([H])c(c3nc2nc([H])c([H])c([H])n2c3N([H])C4([H])C([H])([H])C([H])([H])C([H])([H])C([H])([H])C4([H])[H])c5[H]	10	Active
CHEMBL1995811	O=C(N([H])c4nn([H])c3c([H])c([H])c(c1nnn(c1[H])C([H])([H])c2c([H])c([H])c([H])c([H])c2[H])c([H])c34)c5c([H])c([F])c([H])c([H])c5[F]	10	Active
CHEMBL2007002	[H]n2nc(C(=O)N([H])c1c([H])c([H])c([H])c([H])c1[H])c4c2c([H])c([H])c(c3c([H])c(OC([H])([H])[H])c(O[H])c([H])c3[H])c4[H]	10	Active
CHEMBL1989646	[H]n1nc(N([H])[H])c4c1c([H])c([H])c(c2nnn(c2C([H])([H])[H])C([H])([H])c3c([H])c([H])c([H])c([H])c3[H])c4[H]	10	Active
CHEMBL1983715	O=C(N4C([H])([H])C([H])([H])N(c3c([H])c([H])c(N([H])c1nc([H])c([H])c(n1)c2[s]c(nc2C([H])([H])[H])N([H])C([H])([H])C([H])([H])[H])c([H])c3[H])C([H])([H])C4([H])[H])C([H])([H])[H]	10	Active
CHEMBL1682358	[H]n1nc(N([H])[H])c4c1c([H])c([H])c(c2onc(c2[H])C([H])([H])c3c([H])c([H])c([H])c([H])c3[H])c4[H]	10	Active
CHEMBL1992937	O=[N+]([O-])c3c([H])c([H])c2nc(N([H])c1nc([H])c(c([H])c1[H])C([H])([H])[H])n([H])c2c3[H]	10	Active
CHEMBL1996066	N#Cc4c([H])c([H])c3nc(N([H])[H])n(c2nc(c1c([H])c([H])c([H])c([H])c1[H])c([H])[s]2)c3c4[H]	10	Active

CHEMBL2006450	O=C(N([H])c1nc([Cl])c([H])c(c1[H])c3c([H])n([H])c2nc([H])c([H])c([H])c23)C([H])([H])C([H])([H])C([H])([H])C([H])([H])[H]	12.59	Active
CHEMBL1999811	O=C(N([H])N=C([H])c1c([H])c([H])c(O[H])c([H])c1O[H])N([H])c3c([H])c([H])c([H])c2n[s]nc23	12.59	Active
CHEMBL2003229	[H]n3nc(N([H])[H])c2c(N(C([H])([H])[H])C([H])([H])[H])c([H])c(c1c([H])c([H])nc([H])c1[H])c([H])c23	12.59	Active
CHEMBL1966343	[H]n1nc(N([H])[H])c3c1c([H])c([H])c(c2onc(c2[H])C([H])([H])C([H])(C([H])([H])[H])C([H])([H])[H])c3[H]	12.59	Active
CHEMBL1965702	[Cl]c1nc(nc(c1[H])c3c([H])n([H])c2nc([H])c([H])c([H])c23)N([H])[C@@]4([H])C([H])([H])C([H])([H])C([H])([H])[C@@]([H])(N(C([H])([H])[H])C([H])([H])[H])C4([H])[H]	12.59	Active
CHEMBL396523	O=C(O[H])c1c([H])c([H])c([H])c([H])c1N([H])c2nc(nc([H])c2[H])N([H])c4c([H])c([H])c3n([H])nc([H])c3c4[H]	12.59	Active
CHEMBL2002456	[H]n3nc(N([H])[H])c2c(OC(C([H])([H])[H])(C([H])([H])[H])C([H])([H])[H])c([H])c(c1c([H])c([H])nc([H])c1[H])c([H])c23	12.59	Active
CHEMBL1241473	O=[N+]([O-])c1c([H])nc(nc1N([H])c2c([H])c([H])c([H])c([H])c2C(=O)N([H])[H])N([H])c3c([H])c(OC([H])([H])[H])c(OC([H])([H])[H])c(OC	12.59	Active
CHEMBL1975128	[H]n1nc(N([H])[H])c4c1c([H])c([H])c(c2nnn(c2[I])C([H])([H])c3c([H])c([H])c([H])c([H])c3[H])c4[H]	12.59	Active
CHEMBL1966816	O=C(N([H])c1nc(c([H])c([H])c1[H])C([F])([F])[F])N([H])c3c([H])c([H])nc2c([F])c([H])c([H])c([H])c23	12.59	Active
CHEMBL1682540	[H]n1nc([H])c4c1c([H])c([H])c(c3nc2nc([H])c([H])c([H])n2c3[H])c4[H]	12.59	Active
CHEMBL2003682	O=c2c([H])c(nc(N([H])C([H])([H])c1c([H])c([F])c([H])c([F])c1[H])n2[H])c3c([H])c([H])nc([H])c3[H]	12.59	Active
CHEMBL1976090	[H]N([H])c1nc([H])nc2c1[s]c3nc([H])nc(N([H])[H])c23	12.59	Active
CHEMBL1979176	O=C(N([H])c2c1c([H])c([H])c(OC([H])([H])[H])c([H])c1nc([H])c2[H])N([H])c3nc([H])c([H])c(c3[H])C([F])([F])[F]	15.85	Active
CHEMBL1996791	[I]c1nn(c2nc([H])nc(N([H])[H])c12)[C@]3([H])C([H])([H])C([H])([H])[C@]([H])(O[H])C([H])([H])C3([H])[H]	15.85	Active
CHEMBL1993584	[H]n1c([H])c(c2c1c([H])c([H])c([H])c2[H])C([H])([H])[C@]([H])(O[H])C([H])([H])Oc4c([H])nc([H])c(C([H])=C([H])c3c([H])c([H])nc([H])c3[H])c4[H]	15.85	Active
CHEMBL1987535	O=C(N([H])C([H])([H])C([H])([H])c1[s]c([H])c([H])c1[H])N4C([H])([H])C([H])=C(c3c([H])n([H])c2nc([H])c([H])c([H])c23)C([H])([H])C4([H])[H]	15.85	Active
CHEMBL2006765	O=C(N([H])C([H])([H])C([H])([H])C([H])([H])[H])c3c([H])c([H])c(N([H])c2nc(N([H])C([H])([H])C([F])([F])[F])c1c([H])c([H])n([H])c1n2)c([H])c3[H]	15.85	Active
CHEMBL1967992	N#Cc2c([H])c([H])c(c1c([H])c([H])nc([H])c1[H])c([H])c2N([H])C([H])([H])c3c([H])c([H])c([H])c([H])c3[H]	15.85	Active
CHEMBL1973516	N#Cc1c([H])nc(nc1N([H])c3c([H])c([H])c2n([H])nc([H])c2c3[H])N([H])c5c([H])c([H])c(N4C([H])([H])C([H])([H])N(C([H])([H])C([H])([H])[H])C([H])([H])C4([H])[H])c([H])c5[H]	15.85	Active

CHEMBL458997	O=C(N([H])C([H])([H])[H])c1c([H])c([H])c([H])c([H])c1N([H])c2nc(nc([H])c2[Cl])N([H])c4c([H])c([H])c(N3C([H])([H])C([H])([H])OC([H])([H])C3([H])[H])c([H])c4OC([H])([H])[H]	15.85	Active
CHEMBL210887	O=C(N([H])c2nc1c([H])c([H])c([H])c([H])c1n2C([H])([H])C([H])([H])C([H])([H])O[H])c3c([H])c([H])c([H])c([N+](=O)[O-])c3[H]	15.85	Active
CHEMBL1982957	O=C(N([H])[H])c4c([H])c([H])c([H])c(c3c([H])nc2n([H])c([H])c(c1c([H])c([H])c([H])c([H])c1[H])c2c3[H])c4[H]	15.85	Active
CHEMBL1976455	O=C(N([H])c1nc([H])c([H])nc1[H])N([H])c3c([H])c([H])nc2c([H])c([H])c(c([H])c23)C([F])([F])[F]	19.95	Active
CHEMBL189963	O=C(c2c(c1c([H])nc(nc1n(c2=O)C3([H])C([H])([H])C([H])([H])C([H])([H])C3([H])[H])N([H])c4nc([H])c(c([H])c4[H])N5C([H])([H])C([H])([H])N([H])C([H])([H])C5([H])[H])C([H])([H])[H])C([H])([H])[H]	19.95	Active
CHEMBL461876	[F]c5c([H])c([H])c([H])c(N([H])c1nc(nc2c1c([H])c([H])n2[H])N([H])c3c(OC([H])([H])[H])c([H])c4c(c3[H])N(C(=O)C([H])([H])N(C([H])([H])[H])C([H])([H])[H])C([H])([H])C4([H])[H])c5C(=O)N([H])[H]	19.95	Active
CHEMBL2000071	[H]n2c1nc([H])nc(N([H])C([H])([H])C([H])([H])C([H])([H])O[H])c1c(c2[H])c3c([H])c([H])c([H])c([H])c3[H]	19.95	Active
CHEMBL340384	O=C(N([H])[H])c4c([H])n(c1c([H])c([H])c(O[H])c([H])c1[Cl])c3c([H])c(c2c([H])c([H])nc([H])c2[H])c([H])c([H])c3c4=O	19.95	Active
CHEMBL1993904	O=C(N([H])[H])c1[s]c5c(c1[H])c(c4c([H])c([H])c(N([H])c3nc2c([H])c([H])c([H])c([H])c2o3)c([H])c4[H])c([H])nc5N([H])[H]	19.95	Active
CHEMBL509032	[Cl]c1c([H])nc(nc1N([H])c2c([H])c([H])c([H])c([H])c2[S](=O)(=O)C([H])(C([H])([H])[H])C([H])([H])[H])N([H])c5c(OC([H])([H])[H])c([H])c(N4C([H])([H])C([H])([H])[C@]([H])(N3C([H])([H])C([H])([H])N(C([H])([H])[H])C([H])([H])C3([H])[H])C([H])([H]	19.95	Active
CHEMBL1968459	[H]n2c([H])c(c1nc([H])nc(N([H])C([H])([H])C([H])([H])C([H])([H])C([H])([H])[H])c1[H])c3c2nc([H])c([H])c3[H]	19.95	Active
CHEMBL1991674	[Cl]c2nc(nc(N([H])[C@@]1([H])C([H])([H])C([H])([H])[C@@]([H])(O[H])C([H])([H])C1([H])[H])c2[H])c4c([H])n([H])c3nc([H])c([H])c([H])c34	19.95	Active
CHEMBL1958401	O=c2c([H])c(nc(N([H])C1([H])C([H])([H])C([H])([H])C([H])([H])C([H])([H])C1([H])[H])n2[H])c3c([H])c([H])nc([H])c3[H]	19.95	Active
CHEMBL1973359	O=C(N([H])[H])c3c([H])c([H])c2nc(c1oc(c([H])c1[H])[N+](=O)[O-])c([H])n2c3[H]	19.95	Active
CHEMBL1973516	N#Cc1c([H])nc(nc1N([H])c3c([H])c([H])c2n([H])nc([H])c2c3[H])N([H])c5c([H])c([H])c(N4C([H])([H])C([H])([H])N(C([H])([H])C([H])([H])[H])C([H])([H])C4([H])[H])c([H])c5[H]	19.95	Active
CHEMBL1985406	O=C(N([H])c3nn([H])c2c([H])c([H])c(c1c([H])c([H])c([H])c([F])c1[F])c([H])c23)C([H])([H])C([H])([H])C([H])([H])[H]	19.95	Active
CHEMBL1971289	O=C1C([S]/C(=N\[H])N1[H])=C([H])c2c([H])c([H])c(OC([H])([H])C([H])([H])[H])c([H])c2O[H]	19.95	Active
CHEMBL189963	O=C(c2c(c1c([H])nc(nc1n(c2=O)C3([H])C([H])([H])C([H])([H])C([H])([H])C3([H])[H])N([H])c4nc([H])c(c([H])c4[H])N5C([H])([H])C([H])([H])N([H])C([H])([H])C5([H])[H])C([H])([H])[H])C([H])([H])[H]	19.95	Active
CHEMBL1985095	O=C(N([H])[H])C([H])([H])n5c([H])c(c4c([H])c(c1[s]c2c(c1[H])c([H])c([H])c([H])c2[H])c3n([H])nc([H])c3c4[H])c6nc(nc([H])c56)N([H])[H]	25.12	Active
CHEMBL1993722	[H]n1nc(N([H])[H])c2c1c([H])c([H])c([H])c2c4c([H])c([H])c(N([H])C(=O)N([H])c3c([H])c(C(=O)O[H])c([H])c([H])c3[F])c([H])c4[H]	25.12	Active
CHEMBL1991725	O=C(N([H])[C@]([H])(C([H])([H])Oc3c([H])nc([H])c(c2c([H])c([H])c1c([H])nc([H])c([H])c1c2[H])c3[H])C([H])([H])c5c([H])n([H])c4c([H])c([H])c([H])c([H])c45)C([H])([H])N(C([H])([H])[H])C([H])([H])[H]	25.12	Active

CHEMBL1989293	O=C3N([H])c1c([H])c([H])c([H])c([H])c1N([H])c4c([H])c(c2c([H])c([H])nc([H])c2[F])c([H])c([H])c34	25.12	Active
CHEMBL2000354	O=C2C([S]/C(=N\c1c([H])c([H])c([H])c([H])c1[H])N2[H])=C([H])c3c([H])c([H])c(OC([H])([H])[H])c(O[H])c3[H]	25.12	Active
CHEMBL1979690	[Cl]c1c([H])nc(nc1N([H])c3c([H])c([H])c2n([H])nc([H])c2c3[H])N([H])c5c([H])c([H])c(N4C([H])([H])C([H])([H])N(C([H])([H])[H])C([H])([H])C4([H])[H])c([H])c5[H]	25.12	Active
CHEMBL1973868	[H]Oc3c([H])nc2c([H])c([H])c(c1c([H])c([H])nc([H])c1[H])c([H])c2c3[H]	25.12	Active
CHEMBL1967998	[Cl]c1c([H])nc(nc1N([H])c3c([H])c([H])c2n([H])nc([H])c2c3[H])N([H])c5c([H])c([H])c(N4C([H])([H])C([H])([H])N(C([H])([H])C([H])([H])[H])C([H])([H])C4([H])[H])c([H])c5[H]	25.12	Active
CHEMBL1973098	[S]=C2[S]/C(=C(/[H])c1c([H])c(OC([H])([H])[H])c(O[H])c([H])c1[H])C(=O)N2[H]	25.12	Active
CHEMBL1983589	[Cl]c2nc(nc(N([H])[C@@]1([H])C([H])([H])C([H])([H])[C@@]([H])(N([H])[H])C([H])([H])C1([H])[H])c2[H])c4c([H])n([H])c3nc([H])c([H])c([H])c34	25.12	Active
CHEMBL1993413	O=C(N([H])c1nc([Cl])c([H])c(c1[H])c3c([H])n([H])c2nc([H])c([H])c([H])c23)c4c([H])c([H])c([H])c([H])c4[H]	25.12	Active
CHEMBL1991734	[H]C([H])([H])N3c1c([H])c([H])c(N([H])[H])c([H])c1C([H])(c2c([H])c([H])c([H])c([H])c2[H])c4c([H])c(N([H])[H])c([H])c([H])c34	25.12	Active
CHEMBL1977749	[H]C([H])=C([H])C([H])([H])c2c([H])c([H])c([H])c1c(=NO[H])c([H])c(oc12)c3c([H])c([H])c([H])c([H])c3[H]	25.12	Active
CHEMBL1982660	O=C(N([H])c2c1c([H])c([H])c(OC([H])([H])[H])c([H])c1nc([H])c2[H])N([H])c3nc(c([H])c([H])c3[H])C([F])([F])[F]	25.12	Active
CHEMBL1993661	O=[S](=O)(N([H])C([H])([H])[H])c1c([H])c([H])c([H])c([H])c1N([H])c2nc(nc([H])c2[Cl])N([H])c3c([H])c(OC([H])([H])[H])c(OC([H])([H])[H])c(OC([H])([H])[H])c3[H]	25.12	Active
CHEMBL2004631	[H]n1nc(N([H])[H])c5c1c([H])c([H])c(c2nnn(c2C3([H])C([H])([H])C3([H])[H])C([H])([H])c4c([H])c([H])c([H])c([H])c4[H])c5[H]	25.12	Active
CHEMBL1983348	N#CC([H])([H])C([H])([H])N(C(=O)c4c([H])c([H])c3nc(c2nn([H])c1c([H])c([H])c([H])c([H])c12)n([H])c3c4[H])C([H])([H])C([H])([H])C([H])([H])C([H])([H])[H]	25.12	Active
CHEMBL1971029	O=[S](=O)(N([H])C([H])([H])[H])c1c([H])c([H])c([H])c([H])c1N([H])c2nc(nc([H])c2[Br])N([H])c4c([H])c(N3C([H])([H])C([H])([H])N(C(=O)C([H])([H])[H])C([H])([H])C3([H])[H])c([H])c([H])c4OC([H])([H])[H]	25.12	Active
CHEMBL2000029	[H]n1nc(N([H])[H])c5c1c([H])c([H])c(c2nnn(c2c3c(c([H])c([H])c([H])c3[H])C([H])([H])[H])C([H])([H])c4c([H])c([H])c([H])c([H])c4[H])c5[H]	25.12	Active
CHEMBL1982660	O=C(N([H])c2c1c([H])c([H])c(OC([H])([H])[H])c([H])c1nc([H])c2[H])N([H])c3nc(c([H])c([H])c3[H])C([F])([F])[F]	25.12	Active
CHEMBL1990708	O=C(OC([H])([H])C([H])([H])[H])c2c([H])n([H])n1c(=O)c([H])c(nc12)c3c([H])c([H])nc([H])c3[H]	25.12	Active
CHEMBL1682553	[H]n1nc([H])c4c1c([H])c([H])c(c3nc2nc([H])c([H])c([H])n2c3N([H])C([H])(C([H])([H])[H])C([H])([H])[H])c4[H]	31.62	Active
CHEMBL1969843	[H]n1nc([H])c2c1c([H])c([H])c(c2[H])c3nnn(c3[H])C([H])([H])C4([H])C([H])([H])C([H])([H])OC([H])([H])C4([H])[H]	31.62	Active
CHEMBL1983534	[H]c1c([H])c([H])c(c([H])c1[H])C([H])([H])N([H])c4nc3c([H])c([H])c(c2c([H])c([H])nc([H])c2[H])c([H])c3[s]4	31.62	Active

CHEMBL1985681	N#Cc1c([H])c([H])c4c(c1[H])[C@]([H])(c2nc3c(c([H])c2[H])[C@@]([H])(O[H])C([H])([H])C([H])([H])C3([H])[H])C(=O)N4[H]	31.62	Active
CHEMBL1997534	O=C(N([H])[C@]([H])(c1c([H])c([H])c([H])c(OC([H])([H])[H])c1[H])C([H])([H])[H])c2[s]c(c(c2[H])C([H])([H])[H])c4c([H])c([H])c3n([H])nc(c3c4[H])C([H])([H])[H]	31.62	Active
CHEMBL1995211	[H]n2c1nc([H])nc(N([H])C(C([H])([H])[H])(C([H])([H])[H])C([H])([H])[H])c1c(c2[H])c3c([H])c([H])c([H])c([H])c3[H]	31.62	Active
CHEMBL1879463	O=c1n(c6c(n1c2c([H])c([H])c(c([H])c2[H])C(C#N)(C([H])([H])[H])C([H])([H])[H])c5c([H])c(c4c([H])nc3c([H])c([H])c([H])c([H])c3c4[H])c([H])c([H])c5nc6[H])C([H])([H])[H]	31.62	Active
CHEMBL592030	[H]n1nc([H])c4c1c(c([H])c(c2c([H])n(c3c2nc(nc3[H])N([H])[H])C([H])([H])C([H])([H])C([H])([H])N([H])[H])c4[H])c5[s]c6c(c5[H])c([H])c([H])c([H])c6[H]	31.62	Active
CHEMBL1986767	O=c2c([H])c(nc1c(c([H])n(n12)C([H])([H])[H])c3c([H])c([H])c([H])c([H])c3[H])c4c([H])c([H])nc([H])c4[H]	31.62	Active
CHEMBL1994438	[Cl]c1nc(nc(c1[H])c3c([H])n([H])c2nc([H])c([H])c([H])c23)N([H])[C@@]4([H])[C@]([H])(N([H])[H])C([H])([H])C([H])([H])C([H])([H])C4([H])[H]	31.62	Active
CHEMBL1974617	[H]n1nc([H])c3c1c([H])c([H])c(c2onc(c2[H])C([H])([H])O[H])c3[H]	31.62	Active
CHEMBL1970522	O=C2C([S]/C(=N\c1c([H])c([H])c([H])c([H])c1[H])N2[H])=C([H])c3c([H])c(OC([H])([H])[H])c(O[H])c([H])c3[H]	31.62	Active
CHEMBL1994830	[Cl]c4c([H])c([H])c(Oc2c1c([H])c([s]c1c([H])nc2[H])c3nnc(o3)N([H])[H])c([H])c4[H]	31.62	Active
CHEMBL2001288	[I]c1nn(c2nc([H])nc(N([H])[H])c12)[C@]3([H])O[C@]([H])(C([H])([H])O[H])[C@]([H])(O[H])[C@@]3([H])O[H]	31.62	Active
CHEMBL1971519	[H]n1nc(N([H])[H])c2c1c([H])c([H])c([H])c2c3c([H])c([H])c([F])c([H])c3[H]	39.81	Active
CHEMBL2003817	O=C(N([H])c1nc([H])c([H])nc1[H])N([H])c3c([H])c([H])nc2c([H])c(c([H])c([H])c23)C([F])([F])[F]	39.81	Active
CHEMBL1976936	[Cl]c2nc(nc(N([H])[C@@]1([H])C([H])([H])C([H])([H])C([H])([H])[C@@]([H])(O[H])C1([H])[H])c2[H])c4c([H])n([H])c3nc([H])c([H])c([H])c34	39.81	Active
CHEMBL1993648	[F]c3c([H])c([H])c([H])c(c2c([H])n([H])c1nc([H])c([H])c([H])c12)c3[H]	39.81	Active
CHEMBL2000114	[H]n1c([H])c([S]C([H])([H])[H])c2c1nc([H])nc2N([H])C([H])([H])C([H])([H])C([H])([H])O[H]	39.81	Active
CHEMBL1989136	O=C3N([H])c1c([H])c([H])c([H])c([H])c1N([H])c4c([H])c(c2c([H])c([H])nc([H])c2[H])c([H])c([H])c34	39.81	Active
CHEMBL1988141	O=C(N([H])c2c([H])c([H])c(C(=O)N1C([H])([H])C([H])([H])N([H])C([H])([H])C1([H])[H])c(O[H])c2[H])C([H])([H])C([H])([H])[H]	39.81	Active
CHEMBL1982413	[Cl]c2c([H])c(c1c(nc([H])c([H])c1[H])c2O[H])C([H])([H])N(C([H])([H])[H])C3([H])C([H])([H])C([H])([H])C([H])([H])C([H])([H])C3([H])[H]	50.12	Active
CHEMBL2002479	[Cl]c2c([H])c([H])c1nc(oc1c2[H])N([H])c5c([H])c([H])c(c3c4c(c([H])c(OC([H])([H])C([H])([H])C([H])([H])N([H])[H])c3[H])C(=O)N([H])C4([H])[H])c([H])c5[H]	50.12	Active
CHEMBL2005886	[H]n2c1c([H])c(c([H])c(c1nc2c4c(N([H])C([H])([H])[C@]([H])(O[H])c3c([H])c([H])c([H])c([Cl])c3[H])c([H])c([H])n([H])c4=O)C([H])([H])[H])N5C([H])([H])C([H])([H])OC([H])([H])C5([H])[H]	50.12	Active

CHEMBL243298	O=C(O[H])c1c([H])c([H])c([H])c([H])c1N([H])c2nc(nc([H])c2[H])N([H])c3c([H])c([H])c([H])c([F])c3[H]	50.12	Active
CHEMBL1982610	O=c3n([H])c([H])c([I])c2nc(c1c([H])c([H])c([H])c([Cl])c1[H])c([H])n23	50.12	Active
CHEMBL1973808	O=C(N([H])c2c([H])c([H])c1n([H])nc([H])c1c2[H])c3c([H])c([H])c([Cl])c([H])c3[H]	50.12	Active
CHEMBL2004515	[H]n2c([H])c([H])c1c(nc([H])nc12)c4c([H])c([H])c3OC([H])([H])Oc3c4[H]	50.12	Active
CHEMBL1994693	O=C(N([H])c4c([H])c([H])c3c2c([s]c1nc([H])nc(N([H])[H])c12)C([H])([H])C([H])([H])c3c4[H])N([H])c5c([H])c([H])c([H])c([H])c5[H]	50.12	Active
CHEMBL1987745	O=C3N([H])c1c([H])c([H])c([H])c([H])c1N([H])c4c([H])c(c2c([H])c([H])nc([H])c2[Cl])c([H])c([H])c34	63.1	Active
CHEMBL1972988	[Cl]c1nc([H])nc(c1[H])c3c([H])n([H])c2nc([H])c([H])c([H])c23	63.1	Active
CHEMBL1975534	[I]c2c([H])n(c1nc([H])nc(c12)N([H])[H])C([H])([H])[C@@]([H])(O[H])[C@@]([H])(O[H])C([H])([H])N([H])[H]	63.1	Active
CHEMBL1084546	O=C4N([H])c3c([H])c([H])c(N([H])c2nc(N([H])C([H])([H])c1c(nc([H])c([H])c1[H])N(C([H])([H])[H])[S](=O)(=O)C([H])([H])[H])c(c([H])n2)C([F])([F])[F])c([H])c3C4([H])[H]	63.1	Active
CHEMBL1997335	[H]n2nc(c1c([H])c([H])c(OC([H])([H])[H])c(OC([H])([H])[H])c1[H])c4c([H])c([H])c(OC([H])([H])[H])c3c([H])c([H])c([H])c2c34	63.1	Active
CHEMBL1870106	O=C(c2c([H])nc([H])c1nc(n(c12)C([H])([H])C([H])([H])[H])c3nonc3N([H])[H])N4C([H])([H])C([H])([H])[C@]([H])(N([H])[H])C4([H])[H]	63.1	Active
CHEMBL1964937	O=C(N([H])[C@]([H])(c1c([H])c([H])c([H])c([H])c1[H])C([H])([H])O[H])N4C([H])([H])C([H])=C(c3c([H])n([H])c2nc([H])c([H])c([H])c23)C([H])([H])C4([H])[H]	63.1	Active
CHEMBL2006299	O=C(N([H])C([H])([H])c1c([H])c([H])c([H])c(OC([H])([H])[H])c1[H])c3c([H])c([H])c(c2c([H])c([H])nc([H])c2[H])c([H])c3[H]	63.1	Active
CHEMBL226403	O=C4N([H])C([H])([H])C([H])([H])c5n([H])c(c3c([H])c([H])nc(c2c([H])nc1c([H])c([H])c([H])c([H])c1c2[H])c3[H])c([H])c45	63.1	Active
CHEMBL340921	O=C(c2c([H])n(c1c(c([H])c(O[H])c([H])c1[H])C([H])([H])[H])c4c(c2=O)c([H])c([H])c(c3c([H])c([H])nc([H])c3[H])c4[H])N([H])[H]	63.1	Active
CHEMBL1988608	O=C(N([H])c1c([H])c([H])c([H])c(c1[H])C([H])([H])N([H])c4c(N([H])c3c([H])c([H])c2n([H])nc([H])c2c3[H])c(=O)c4=O)C([H])([H])[H]	63.1	Active
CHEMBL1966040	[F]C([F])([F])c1c([H])c([H])c([H])c(c1[H])C([H])([H])N([H])c2nc([H])nc(c2[H])c4c([H])n([H])c3nc([H])c([H])c([H])c34	63.1	Active
CHEMBL2005216	O=[S](=O)(N([H])[C@]4([H])C([H])([H])C([H])([H])[C@]([H])(N([H])c1nc(nc([Cl])c1[H])c3c([H])n([H])c2nc([H])c([H])c([H])c23)C([H])([H])C4([H])[H])C([H])([H])[H]	63.1	Active
CHEMBL526133	O=c4nc(c2c([H])c([H])c1n([H])nc([H])c1c2[H])c([H])c(c3c([H])c([H])c([H])c([H])c3[H])n4[H]	63.1	Active
CHEMBL1986530	O=C(c1c([H])c(c([H])c([H])c1[F])C([H])([H])c2nn([H])c(=O)c3c2C([H])([H])C([H])([H])C([H])([H])C3([H])[H])N5C([H])([H])C([H])([H])N(c4nc([H])c([H])c([H])n4)C([H])([H])C5([H])[H]	63.1	Active
CHEMBL1725279	[H]Oc3c1c([H])c([H])c([H])c([H])c1c2N=Nc4c2c3c([H])c([H])c4[H]	63.1	Active

CHEMBL1985367	[H]C([H])([H])c3c([H])c([H])nc(N([H])c1nc(nc(c1[H])C([H])([H])[H])c2c([H])c([H])c([H])c([H])c2[H])c3[H]	63.1	Active
CHEMBL1986756	O=C(N([H])c2c1c([H])c([H])c(c([H])c1nc([H])c2[H])C([H])([H])C([H])([H])[H])N([H])c3nc(c([H])c([H])c3[H])C([F])([F])[F]	63.1	Active
CHEMBL2006778	O=C(N([H])C([H])([H])[H])C([H])=C([H])c4c([H])nc(N([H])[H])c3c(c2c([H])c([F])c1n([H])c(c([H])c1c2[H])C([H])([H])[H])c([H])[s]c34	63.1	Active
CHEMBL1981511	O=C4c2c([H])c([H])c(c1c([H])c(nc([H])c1[H])C([H])([H])[H])c([H])c2N([H])c3c([H])c([H])c([H])c([H])c3N4[H]	63.1	Active
CHEMBL2007296	O=C(N([H])[C@]([H])(c1c([H])c([H])c([H])c(OC([H])([H])C([H])([H])C([H])([H])[H])c1[H])C([H])([H])[H])c3c([H])c([H])c(c2c([H])c([H])nc([H])c2[H])c([H])c3[H]	63.1	Active
CHEMBL1682545	[H]n1nc([H])c4c1c([H])c([H])c(c2nc([s]c2[H])N([H])c3c([H])c([H])c([H])c([H])c3[H])c4[H]	79.43	Active
CHEMBL1990482	O=C(N([H])[H])c2c([H])c([H])c([H])c1n([H])c(nc12)c3c([H])c([H])nc([H])c3[H]	79.43	Active
CHEMBL482967	[H]C([H])([H])c1nc([s]c1c2nc(nc([H])c2[H])N([H])c4c([H])c([H])c(N3C([H])([H])C([H])([H])OC([H])([H])C3([H])[H])c([H])c4[H])N([H])[H]	79.43	Active
CHEMBL1984363	O=C(N([H])c1c([H])c(c([F])c([H])c1[H])C([H])([H])[H])C([H])([H])c4c([H])c([H])c(c3c([H])c([H])c([H])c2n([H])nc(N([H])[H])c23)c([H])c4[H]	79.43	Active
CHEMBL2003341	O=C(N([H])[C@@]([H])(c1c([H])c([Cl])c([H])c([Cl])c1[H])C([H])([H])O[H])c3c([H])c([H])c(c2c([H])c([H])nc([H])c2[H])c([H])c3[H]	79.43	Active
CHEMBL1972355	[H]n1c(nc2c([H])c([H])c([H])c([H])c12)c4c(OC([H])([H])[H])c3c(oc(c([H])c3=O)C([H])([H])[H])c([H])c4O[H]	79.43	Active
CHEMBL220057	O=C(N([H])c1c([H])c([H])c([H])c(c1[H])C([H])([H])[H])N([H])c4c([H])c([H])c(c3c([H])c([H])c([H])c2n([H])nc(N([H])[H])c23)c([H])c4[H]	79.43	Active
CHEMBL1992634	[Br]c3c([H])nc2n([H])c([H])c(c1c([H])c([H])c([H])c([H])c1[H])c2c3[H]	79.43	Active
CHEMBL1970104	[H]n3nc(c2nc1c([H])c([H])c(c([H])c1n2[H])C(C([H])([H])[H])(C([H])([H])[H])C([H])([H])[H])c4c3c([H])c([H])c([H])c4[H]	79.43	Active
CHEMBL1992536	[H]n1nc([H])c4c1c([H])c([H])c(N([H])c3c(N([H])C([H])([H])c2c([H])c([H])c(OC([H])([H])[H])c([H])c2[H])c(=O)c3=O)c4[H]	79.43	Active
CHEMBL1983945	[H]n1c([H])c(c2c1c([H])c([H])c([H])c2[H])C([H])([H])[C@@]([H])(N([H])[H])C(=O)N([H])c4c([H])nc([H])c(C([H])=C([H])c3c([H])c([H])nc([H])c3[H])c4[H]	79.43	Active
CHEMBL243518	O=C(O[H])c3c(N([H])c1nc(nc([H])c1[H])N([H])c2c([H])c([H])c(c([F])c2[H])C([H])([H])[H])c([H])c([H])c([H])c3[H]	79.43	Active
CHEMBL1998611	O=C(N([H])[H])c2[s]c1c([Br])c([H])c([H])c([Cl])c1c2N([H])[H]	79.43	Active
CHEMBL1970104	[H]n3nc(c2nc1c([H])c([H])c(c([H])c1n2[H])C(C([H])([H])[H])(C([H])([H])[H])C([H])([H])[H])c4c3c([H])c([H])c([H])c4[H]	79.43	Active
CHEMBL1974328	O=C(c1nc([H])c(nc1N([H])c2c([H])c([H])c([H])c(c2[H])C([H])([H])[H])N([H])[C@@]3([H])C([H])([H])C([H])([H])C([H])([H])C([H])([H])[C@]3([H])N([H])[H])N([H])[H]	79.43	Active
CHEMBL1997041	[H]n3c2nc([H])nc(c1c([H])c(c([F])c([H])c1[H])C([H])([H])[H])c2c([H])c3[H]	79.43	Active

CHEMBL1965631	O=C(N([H])c4nn([H])c3c([H])c([H])c(c1nnn(c1[H])C([H])([H])c2c([H])c([H])c([H])c([H])c2[H])c([H])c34)C([H])([H])N(C([H])([H])[H])C([H])([H])[H]	79.43	Active
CHEMBL1968868	N#Cc1nc(c([H])c([H])c1[H])N([H])C(=O)N([H])c3c([H])c([H])nc2c([H])c(c([H])c([H])c23)C([F])([F])[F]	79.43	Active
CHEMBL1973142	O=C(N([H])c2c([s]c1c([H])c([H])c([Cl])c([Cl])c12)C(=O)N([H])[H])C([H])([H])[H]	79.43	Active
CHEMBL223360	[F]c1c([H])c([H])c(c([H])c1N([H])C(=O)N([H])c4c([H])c([H])c(c3c([H])c([H])c([H])c2n([H])nc(N([H])[H])c23)c([H])c4[H])C([H])([H])[H]	79.43	Active
CHEMBL1976134	[H]n4nc([H])c3c([H])c([H])c(c2c([H])c1c([H])c([H])n([H])c1nc2[H])c([H])c34	79.43	Active
CHEMBL2001641	[H]n1nc([H])c3c1c([H])c([H])c(n2nnc(c2[H])C(O[H])(C([H])([H])[H])C([H])([H])[H])c3[H]	79.43	Active
CHEMBL1965845	O=C(N([H])[C@]([H])(c1c([H])c([H])c([H])c(OC([H])([H])[H])c1[H])C([H])([H])[H])N4C([H])([H])C([H])=C(c3c([H])n([H])c2nc([H])c([H])c([H])c23)C([H])([H])C4([H])[H]	79.43	Active
CHEMBL2007375	O=C(N([H])c2c([H])c([H])nc1c([H])c([H])c(C#CC([H])([H])[H])c([H])c12)N([H])c3nc(c([H])c([H])c3[H])C([F])([F])[F]	79.43	Active
CHEMBL1988662	[H]n3nc(N([H])[H])c2c(OC([H])([H])C([H])([H])C([H])([H])N(C([H])([H])[H])C([H])([H])[H])c([H])c(c1c([H])c([H])nc([H])c1[H])c([H])c23	79.43	Active
CHEMBL1996702	O=C(c4c([H])c([H])c3N([H])c2c(c([H])c([H])c(c1c([H])c(OC([H])([H])[H])c(O[H])c([H])c1[H])c2[H])C(=O)N([H])c3c4[H])N([H])C([H])([H])C([H])([H])C([H])([H])N(C([H])([H])[H])C([H])([H])[H]	79.43	Active
CHEMBL2005528	O=C(OC([H])([H])C([H])([H])[H])c2c([H])c([H])c1n([H])c3c(c1c2[H])C([H])([H])C([H])([H])N([H])C3=O	79.43	Active
CHEMBL21156	[F]c3c([H])c([H])c2c1nc(n([H])c1c4c(c2c3[H])c(=O)n([H])c([H])c4[H])C(C([H])([H])[H])(C([H])([H])[H])C([H])([H])[H]	79.43	Active
CHEMBL583144	O=c1n([H])c([H])nc2c1[s]c3c([Cl])c([H])c([H])c([Cl])c23	89.6	Active
CHEMBL2002599	O=C(N([H])c1nc([H])c([H])nc1[H])N([H])c5c([H])c([H])nc4c([H])c([H])c(N3C([H])([H])[C@@]2([H])C([H])([H])OC([H])([H])[C@@]2([H])C3([H])[H])c([H])c45	100	Active
CHEMBL1964444	O=C(N([H])c2c([H])c([H])nc1c([H])c([H])c(c([H])c12)C([H])([H])[H])N([H])c3nc(c([H])c([H])c3[H])C([F])([F])[F]	100	Active
CHEMBL244378	O=C(O[H])c1c([H])c([H])c([H])c([H])c1N([H])c2nc(nc([H])c2[H])N([H])c4c([H])c([H])c3c([H])nn([H])c3c4[H]	100	Active
CHEMBL2003286	O=C2[S]/C(=C(/[H])c1c([H])c(OC([H])([H])[H])c(O[H])c([H])c1[H])C(=O)N2[H]	100	Active
CHEMBL1982982	O=C(N([H])[H])c2c([H])c([H])c(c1c([H])c([H])nc([H])c1[H])c([H])c2N([H])C([H])([H])c3c([H])c([H])c([H])c([F])c3[H]	100	Active
CHEMBL1164265	[F]c1c(c([Cl])c([F])c([H])c1[H])C([H])([H])N5c4c(nc([H])c(c2c([H])c([H])c(nc2[H])N3C([H])([H])C([H])([H])N(C([H])([H])[H])C([H])([H])C3([H])[H])c4[H])N([H])C([H])([H])C5([H])[H]	100	Active
CHEMBL1993424	O=[S](=O)(N([H])[H])c4c([H])c([H])c([H])c(N([H])c2nc([H])c1c([H])c([H])n(c1n2)C([H])([H])c3c([H])c([H])c([H])c([H])c3[H])c4[H]	100	Active
CHEMBL1964692	O=C(N([H])c3c([H])c([H])c(c2c1c(nn(c1c([H])nc2[H])C([H])([H])[H])N([H])[H])c([H])c3[H])N([H])c4c([H])c([H])c([H])c(c4[H])C([F])([F])[F]	100	Active

CHEMBL583144	O=c1n([H])c([H])nc2c1[s]c3c([Cl])c([H])c([H])c([Cl])c23	100	Active
CHEMBL1967544	O=[S](=O)(c2c1c([H])c([H])c([H])c(N(C([H])([H])[H])C([H])([H])[H])c1c([H])c([H])c2[H])N(c5c([H])nc([H])c(c4c([H])c([H])c3c([H])nc([H])c([H])c3c4[H])c5[H])C([H])([H])C([H])([H])N([H])[H]	100	Active
CHEMBL2007603	O=C(N([H])c1nc(c([H])c([H])c1[H])C([F])([F])[F])N([H])c3c([H])c([H])nc2c([H])c([H])c([F])c([H])c23	100	Active
CHEMBL1965131	O=C(c1c([H])c([H])c(c([H])c1[H])C([H])([H])[H])N([H])c3c([s]c2c([H])c([H])c([Cl])c([Cl])c23)C(=O)N([H])[H]	100	Active
CHEMBL1965169	[Br]c3nc2c([H])c(c1c([H])c([H])c(c([H])c1[H])C([H])([H])[H])n([H])c2nc3[H]	100	Active
CHEMBL1971606	O=C(N([H])[C@]([H])(c1c([H])c([H])c([H])c(OC([H])([H])C([H])([H])C([H])([H])[H])c1[H])C([H])([H])[H])c3c([H])c([H])c(c2c([H])c([H])nc([H])c2[F])c([H])c3[H]	100	Active
CHEMBL1967878	[F]c1c([H])nc(nc1N([H])[C@]3([H])[C@]2([H])C([H])=C([H])[C@@]([H])(C2([H])[H])[C@]3([H])C(=O)N([H])[H])N([H])c4c([H])c(c(c([H])c4[H])N5C([H])([H])C([H])([H])N(C([H])([H])[H])C([H])([H])C5([H])[H])C([H])([H])[H]	100	Active
CHEMBL522892	[F]c5c4c(c(c3nc2c([H])c([H])c(N1C([H])([H])C([H])([H])N(C([H])([H])[H])C([H])([H])C1([H])[H])c([H])c2n3[H])c(=O)n([H])c4c([H])c([H])c5[H])N([H])[H]	100	Active
CHEMBL2003271	O=C(N([H])[H])c2[s]c1c([H])c([H])c([Cl])c([Cl])c1c2N([H])C(=O)c3c([H])c([H])c([Cl])c([H])c3[H]	100	Active
CHEMBL497151	O=c4nc(c1[s]c3c(c1[H])c(Oc2c([H])c([H])c([I])c([H])c2[H])c([H])nc3[H])n([H])o4	125.89	Active
CHEMBL1982992	[Cl]c3c([H])[s]c2nc([H])nc(N([H])c1c([H])c([H])c([H])c(O[H])c1[H])c23	125.89	Active
CHEMBL1989471	[H]n2c1nc([H])c([H])c(c1c(=O)c3c2c([H])c(c([H])c3[H])C([H])(C([H])([H])[H])C([H])([H])[H])C([H])([H])[H]	125.89	Active
CHEMBL1977681	O=C(N([H])C([H])([H])[H])c1[s]c2c(c1[H])c(nn2[H])c3c([H])c([H])c([H])c([H])c3[F]	125.89	Active
CHEMBL1988537	O=C(c1c([H])c([H])c(OC([H])([H])[H])c([H])c1[H])N3c2c([H])c([H])c([H])c([H])c2[S]c4c([Cl])c([H])nc([H])c34	125.89	Active
CHEMBL1990254	O=C(N([H])[C@@]([H])(c1c([H])c([H])c([H])c([H])c1[H])C([H])([H])N([H])[H])c3c([H])c([H])c(c2c([H])c([H])nc([H])c2[H])c([H])c3[H]	125.89	Active
CHEMBL1971943	[H]n1c([H])c(c2c1nc([H])c([H])c2Oc4c([F])c([H])c(N([H])c3nc(nc([Cl])c3[H])N([H])[H])c([H])c4[F])C([H])([H])[H]	125.89	Active
CHEMBL1990254	O=C(N([H])[C@@]([H])(c1c([H])c([H])c([H])c([H])c1[H])C([H])([H])N([H])[H])c3c([H])c([H])c(c2c([H])c([H])nc([H])c2[H])c([H])c3[H]	125.89	Active
CHEMBL360847	O=C6C3=C(c1c([H])n(c2c1c([H])c([H])c([H])c2[H])C([H])([H])C([H])([H])[C@@]([H])(OC([H])([H])C([H])([H])n4c([H])c3c5c4c([H])c([H])c([H])c5[H])C([H])([H])N(C([H])([H])[H])C([H])([H])[H])C(=O)N6[H]	125.89	Active
CHEMBL196363	O=C(N([H])c1c([H])c([H])c([H])c(c1[H])C([H])([H])[H])N([H])c4c([H])c([H])c(c3c([H])[s]c2nc([H])nc(N([H])[H])c23)c([H])c4[H]	125.89	Active
CHEMBL475251	[F]c1c([H])nc(nc1N([H])c3nc2c(OC(C(=O)N2[H])(C([H])([H])[H])C([H])([H])[H])c([H])c3[H])N([H])c4c([H])c(OC([H])([H])[H])c(OC([H])([H])[H])c(OC([H])([H])[H])c4[H]	125.89	Active
CHEMBL1994241	N#C/C(=C(/[H])c1c([H])c(OC([H])([H])[H])c(O[H])c([H])c1[Br])c3nc2c([H])c([H])c([H])c([H])c2n3[H]	125.89	Active

CHEMBL2000345	N#C/C(=C(\[H])c1c([H])c(OC([H])([H])[H])c(O[H])c([H])c1[Br])c3nc2c([H])c(c([H])c([H])c2n3[H])C([H])([H])[H]	125.89	Active
CHEMBL1992371	O=C(N([H])c1nc([H])c([H])nc1[H])N([H])c3c([H])c([H])c([H])c2c(O[H])c([H])c([H])c([H])c23	125.89	Active
CHEMBL1986666	O=C(O[H])c1c([H])c([H])c([H])c([H])c1N([H])c2nc([H])nc(n2)N([H])c4c([H])c([H])c3c([H])nn([H])c3c4[H]	125.89	Active
CHEMBL1975900	O=c3c(N([H])C([H])([H])c1c([H])c([H])c([Cl])c([Cl])c1[H])c(N([H])c2c([H])c([H])nc([H])c2[H])c3=O	125.89	Active
CHEMBL2002099	N#CC([H])([H])[C@@]([H])(n1nc([H])c(c1[H])c3nc([H])nc2n([H])c([H])c([H])c23)[C@@]4([H])C([H])([H])C([H])([H])C([H])([H])C4([H])[H]	125.89	Active
CHEMBL242865	O=C(O[H])c1c([H])c([H])c([H])c([H])c1N([H])c2nc(nc([H])c2[H])N([H])c3c([H])c([H])c([N+](=O)[O-])c([H])c3[H]	125.89	Active
CHEMBL1242373	O=C(N([H])c1c([H])c([H])c([H])c(c1[H])C([H])([H])[H])N([H])c2c([H])c([H])c4c(c2[H])C([H])([H])C([H])([H])c5[s]c3nc([H])nc(N([H])[H])c3c45	125.89	Active
CHEMBL1981215	O=C(N([H])c1nc(c([H])c([H])c1[H])C([F])([F])[F])N([H])c3c([H])c([H])nc2c([H])c(OC([F])([F])[H])c([H])c([H])c23	125.89	Active
CHEMBL2004156	O=c4c([H])c(c1c([H])c([H])c([H])c([F])c1[H])n([H])c3c(c2c([H])c([H])c([F])c([H])c2[H])c([H])nn34	158.49	Active
CHEMBL2002992	O=C(N([H])c2c([H])c([H])nc1n([H])c([H])c([H])c12)[C@@]3([H])C([H])([H])C([H])([H])[C@]([H])([C@@]([H])(N([H])[H])C([H])([H])[H])C([H])([H])C3([H])[H]	158.49	Active
CHEMBL1997846	[Cl]c1nc(nc(c1[H])N([H])C2([H])C([H])([H])C([H])([H])C([H])([H])C([H])([H])C2([H])[H])c4c([H])n([H])c3nc([H])c([H])c([H])c34	158.49	Active
CHEMBL1516890	O=C4/C(=C(\[H])c1c([H])n(c2c1c([H])c([H])c([H])c2[H])C([H])([H])[H])c3nc([H])c([H])c([H])c3N4[H]	158.49	Active
CHEMBL1993335	[Br]c4nc3c([H])c(c1c([H])n(c2c1c([H])c([F])c([H])c2[H])C([H])([H])[H])n([H])c3nc4[H]	158.49	Active
CHEMBL1973893	O=[S](=O)(N([H])c3c([H])c([H])c(c2c([H])c([H])c1n([H])nc(N([H])C(=O)C([H])([H])[H])c1c2[H])c([H])c3[H])C([H])([H])C([H])([H])C([H])([H])[H]	158.49	Active
CHEMBL1967513	O=C(N([H])c1nc(c([H])c([H])c1[H])C([F])([F])[F])N([H])c3c([H])c([H])nc2c([H])c([H])c([Cl])c([H])c23	158.49	Active
CHEMBL1979970	O=C(N([H])c3nn([H])c2nnc(c1c([H])c([H])c([H])c([F])c1[F])c([H])c23)C([H])([H])C([H])([H])C([H])([H])[H]	158.49	Active
CHEMBL1975927	[H]C([H])([H])Oc4c([H])c([H])c(n1c([H])c([H])c2c([H])nc(nc12)N([H])c3c([H])c(OC([H])([H])[H])c(OC([H])([H])[H])c(OC([H])([H])[H])c3[H])c([H])c4[H]	158.49	Active
CHEMBL1998545	O=C(N([H])[C@]([H])(C([H])([H])O[H])C([H])([H])c1c([H])c([H])c([H])c([H])c1[H])c3c([H])c([H])c(c2c([H])c([H])nc([H])c2[H])c([H])c3[H]	158.49	Active
CHEMBL1989518	[H]N([H])C([H])([H])C([H])([H])Oc2c([H])nc([H])c(C([H])=C([H])c1c([H])c([H])nc([H])c1[H])c2[H]	158.49	Active
CHEMBL1973483	O=C(N([H])c1nc(c([H])c([H])c1[H])C([F])([F])[F])N([H])c3c([H])c([H])nc2c([H])c(c([H])c([H])c23)C([F])([F])[F]	158.49	Active
CHEMBL1987430	O=C(N([H])c2c([H])c([H])nc1c([H])c([H])c(c([H])c12)C([H])([H])C([H])([H])C([H])([H])[H])N([H])c3nc(c([H])c([H])c3[H])C([F])([F])[F]	158.49	Active

CHEMBL1978195	O=C(N([H])[H])c1[s]c3c(c1[H])c(c2c([H])c([H])c([Br])c([H])c2[H])c([H])nc3[H]	158.49	Active
CHEMBL2007559	O=C(N([H])C([H])([H])C([H])([H])C([H])([H])[H])c3c([H])c([H])c(N([H])c2nc(N([H])C([H])([H])C([F])([F])[F])c1[s]c([H])c([H])c1n2)c([H])c3[H]	158.49	Active
CHEMBL2006237	O=C(c4c3nc(c2nc(N([H])C([H])([H])C([H])([H])C([H])([H])N1C([H])([H])C([H])([H])N(C([H])([H])[H])C([H])([H])C1([H])[H])c([H])c([H])c2[H])n([H])c3c([H])c([H])c4[H])N([H])[H]	158.49	Active
CHEMBL2004443	O=c4c([H])c(c1c([H])c([H])c([F])c([H])c1[H])n([H])c3c(c2c([H])c([H])c([F])c([H])c2[H])c([H])nn34	158.49	Active
CHEMBL2000894	O=C(c2c([H])n(c1c(c([H])c(O[H])c([H])c1[H])C([H])([H])[H])c4c(c2=O)c([H])c([H])c(c3c([H])c([H])nc([H])c3[H])c4[H])N([H])N([H])[H]	158.49	Active
CHEMBL1974310	O=C(N([H])[H])c1[s]c3c(c1[H])c(c2c([H])c([H])c([F])c([H])c2[H])c([H])nc3[H]	158.49	Active
CHEMBL1972489	O=C(N([H])c2c([H])c([H])c1c([H])nn([H])c1c2[H])c3c([H])c([H])c([Cl])c([H])c3[H]	158.49	Active
CHEMBL1983268	O=C(c2c([H])c([H])c(N1C([H])([H])C([H])([H])N(C([H])([H])[H])C([H])([H])C1([H])[H])c([H])c2N([H])C3([H])C([H])([H])C([H])([H])OC([H])([H])C3([H])[H])N([H])c5nn([H])c4c([H])c([H])c(c([H])c45)C([H])([H])c6c([H])c([F])c([H])c([F])c6[H]	199.53	Active
CHEMBL116070	O=C(N([H])[H])c1[s]c3c(c1[H])c(Oc2c([H])c([H])c([Br])c([H])c2[H])c([H])nc3[H]	199.53	Active
CHEMBL1980671	O=C2c1c(nc([H])nc1N([H])c3c(N2[H])c([H])c([H])c([H])c3[H])N([H])[H]	199.53	Active
CHEMBL1982135	O=C(N([H])c1c(c([H])c([H])c([H])c1[H])C([H])([H])[H])N([H])c2c([H])c([H])c4c(c2[H])C([H])([H])C([H])([H])c5[s]c3nc([H])nc(N([H])[H])c3c45	199.53	Active
CHEMBL1461728	O=C(N([H])c2c([H])c([H])c1n([H])nc([H])c1c2[H])c3c([H])c([H])c([H])c([F])c3[H]	199.53	Active
CHEMBL1982924	O=C(c1c([H])c([H])c([H])c(OC([H])([H])[H])c1[H])N([H])c4c([H])nc3n([H])c([H])c(c2c([H])c([H])c([H])c([H])c2[H])c3c4[H]	199.53	Active
CHEMBL1997764	O=C(c1c([H])c([H])c(OC([H])([H])[H])c([H])c1[H])N([H])c4c([H])nc3n([H])c([H])c(c2c([H])c([H])c([H])c([H])c2[H])c3c4[H]	199.53	Active
CHEMBL191003	O=[S](=O)(c3c([H])c([H])c(N([H])c1nc(N([H])[H])n(n1)C(=O)c2c([F])c([H])c([H])c([H])c2[F])c([H])c3[H])N([H])[H]	199.53	Active
CHEMBL1974664	[H]n2c1c(nc([H])nc1c3c2c([H])c([H])c([H])c3[H])N([H])N=C([H])c4c([H])c([H])c(OC([H])([H])[H])c(OC([H])([H])[H])c4[H]	199.53	Active
CHEMBL411903	O=C(N([H])c3c([H])c([H])c(/C2=N/c1c(nc([H])nc1N([H])C([H])([H])C2([H])[H])N([H])[H])c([H])c3[H])N([H])c4c([H])c(c([H])c([H])c4[F])C([F])([F])[F]	199.53	Active
CHEMBL443962	[Br]c3c2nc([H])c(c1c([H])c([H])c(OC([H])([H])[H])c([H])c1[H])c([H])n2nc3[H]	199.53	Active
CHEMBL1986603	O=C(N([H])c1noc(c1[H])C([H])([H])[H])C([H])([H])C([H])([H])N([H])C(=O)N([H])c2nc(c([s]2)c4c([H])c([H])c(n3nc([H])c([H])c3[H])c([H])c4[H])C([H])([H])[H]	199.53	Active
CHEMBL2005478	[Br]c4nc3c([H])c(c2c1c(c([H])c([H])c(OC([H])([H])[H])c1[H])n(c2[H])C([H])([H])[H])n([H])c3nc4[H]	199.53	Active
CHEMBL1981782	O=C(N([H])C([H])([H])c1c([H])c([H])c([Cl])c([Cl])c1[H])N([H])c3c([H])c([H])c2n([H])nc([H])c2c3[H]	199.53	Active

CHEMBL1988805	N#Cc3c([H])c([H])c2nc(N([H])[H])n(c1nc([H])c([H])[s]1)c2c3[H]	199.53	Active
CHEMBL1968406	O=C(N([H])c1c([H])c([H])c([H])c(c1[H])C([H])([H])C([H])([H])[H])N([H])c2c([H])c([H])c4c(c2[H])C([H])([H])C([H])([H])c5[s]c3nc([H])nc(N([H])[H])c3c45	199.53	Active
CHEMBL1972454	O=C(n1nc([H])c2c([H])c(N([H])[H])c([H])c([H])c12)C([H])([H])[H]	199.53	Active
CHEMBL41783	O=C(N([H])c3nn([H])c2nnc(c1c([H])c([H])c([H])c([F])c1[F])c([H])c23)C([H])([H])C([H])([H])C([H])([H])N(C([H])([H])[H])C([H])([H])[H]	199.53	Active
CHEMBL1976220	O=C(N([H])c3c([H])c([H])c(c2c([H])[s]c1c([H])c([H])nc(N([H])[H])c12)c([H])c3[H])N([H])c4c([H])c([H])c([H])c([Br])c4[H]	199.53	Active
CHEMBL508928	O=C(N([H])c1c([H])c([H])c([H])c(c1[H])C([H])([H])[H])N([H])c5c([H])c([H])c(c4c([H])nc3c(c2c([H])nn(c2[H])C([H])([H])[H])c([H])nn3c4N([H])[H])c([H])c5[H]	199.53	Active
CHEMBL2004872	O=C(N([H])[C@@]([H])(c1c([H])c([H])c([H])c(OC([H])([H])[H])c1[H])C([H])([H])[H])c3c([H])c([H])c(c2nc([H])nc([H])c2[H])c([H])c3[H]	199.53	Active
CHEMBL1983111	O=C(N([H])C([H])([H])C(=O)N4[C@@]5([H])c3c(c([H])c(N([H])c1nc([H])c(c(n1)N([H])[C@@]2([H])C([H])([H])C([H])([H])C2([H])[H])C([F])([F])[F])c([H])c3[H])[C@]4([H])C([H])([H])C5([H])[H])C([H])([H])[H]	199.53	Active
CHEMBL1969372	[F]c4c([H])c([H])c(c2nc1c([H])nc([H])c([H])n1c2c3nc(nc([H])c3[H])N([H])C([H])([H])C([H])([H])OC([H])([H])[H])c([H])c4[H]	199.53	Active
CHEMBL1969301	O=C(N([H])c3nc2c([H])c(N(C(=O)C1([H])C([H])([H])C([H])([H])C([H])([H])C([H])([H])C1([H])[H])C([H])([H])[H])c([H])c([H])c2n3C([H])([H])C([H])([H])C([H])([H])C([H])([H])[H])c4c([H])c([H])c([H])c(C#N)c4[H]	199.53	Active
CHEMBL2004892	O=[S](=O)(N([H])c3c([H])c([H])c(c2c([H])c([H])c1n([H])nc(N([H])[H])c1c2[H])c([H])c3[H])C([H])([H])[H]	199.53	Active
CHEMBL1994555	O=C(c3c([H])c([H])c(c2c(c1nc([H])c([H])nc1n2[H])C([H])([H])C([H])([H])[H])c([H])c3[H])C([H])([H])[H]	251.19	Active
CHEMBL2000393	O=c2n([H])c1c([H])nc(nc1n2[C@]3([H])c4c(OC([H])([H])C3([H])[H])c([F])c([H])c([H])c4[H])n5c([H])nc6c([H])c([H])c([F])c([H])c56	251.19	Active
CHEMBL1682546	[H]n1nc([H])c4c1c([H])c([H])c(c2nc([s]c2[H])N([H])C([H])([H])c3c([H])c([H])c([H])c([H])c3[H])c4[H]	251.19	Active
CHEMBL1997611	O=C(OC([H])([H])C([H])([H])[H])c3nc2c(=O)n([H])c1c([H])c([N+](=O)[O-])c(N([H])C(=O)C([H])([H])[H])c([H])c1n2c3C([H])([H])[H]	251.19	Active
CHEMBL1979252	O=c3c(N([H])C([H])([H])c1c([H])c([H])c([H])c(c1[H])C([F])([F])[F])c(N([H])c2c([H])c([H])nc([H])c2[H])c3=O	251.19	Active
CHEMBL2002649	[H]n1c([H])c(nc1[H])C([H])=C3c2c(c([H])c([H])c(OC([H])([H])[H])c2[H])N([H])C3=O	251.19	Active
CHEMBL1966842	O=C(N([H])c5c([H])c([H])c([H])c(c2nc1[s]c([H])c([H])n1c2c3nc(nc([H])c3[H])N([H])c4c(OC([H])([H])[H])c([H])c([H])c(c4[H])C([H])([H])N(C([H])([H])[H])C([H])([H])[H])c5[H])C([H])([H])c6c([H])c([H])c([H])c([H])c6[H]	251.19	Active
CHEMBL1975121	O=C(N([H])[C@]([H])(c1c([H])c([H])c([H])c(OC([H])([H])[H])c1[H])C([H])([H])[H])c3c([H])c([H])c(c2c([H])c([H])nc([H])c2[H])c([H])c3[H]	251.19	Active
CHEMBL1992581	O=C(N([H])c1c([H])c([H])c([S]C([F])([F])[H])c([H])c1[H])C([H])([H])c3c([H])c([H])c(c2c([H])c([H])c([H])nc2[H])c([H])c3[H]	251.19	Active
CHEMBL1975647	O=C(N([H])c1nc([H])c([H])nc1[H])N([H])c3c([H])c([H])nc2c([F])c([H])c([H])c([H])c23	251.19	Active

CHEMBL1978014	O=C(n3nc(N([H])[H])c4c([H])c(c1nnn(c1[H])C([H])([H])c2c([H])c([H])c([H])c([H])c2[H])c([H])c([H])c34)C5([H])C([H])([H])C([H])([H])N(C([H])([H])[H])C([H])([H])C5([H])[H]	251.19	Active
CHEMBL1972258	[H]N([H])C([H])([H])C([H])([H])C([H])([H])Oc2c([H])nc([H])c(C([H])=C([H])c1c([H])c([H])nc([H])c1[H])c2[H]	251.19	Active
CHEMBL1977346	O=C2N([H])c1c([H])c([H])c([H])c([H])c1N([H])c3nnc([I])c([H])c23	251.19	Active
CHEMBL1964413	[F]C([F])([F])c1c([H])c([H])c(c(c1[H])C([F])([F])[F])C([H])([H])n2nnc(c2[H])c4c([H])c([H])c3n([H])nc([H])c3c4[H]	251.19	Active
CHEMBL1991356	O=c3c2c([H])c(c1c([H])c([H])c([H])c(OC([H])([H])[H])c1[H])c([H])n2c4c(n3[H])c([H])c([H])c([H])c4[H]	251.19	Active
CHEMBL2000934	O=C(N([H])c2c([H])c([H])nc1c([H])c([H])c(c([H])c12)C([H])([H])C([H])([H])[H])N([H])c3nc(c([H])c([H])c3[H])C([F])([F])[F]	251.19	Active
CHEMBL1988838	O=[S](=O)(c3c([H])c([H])c([H])c(N([H])c1nc([H])c(c(n1)N([H])c2c([H])c([H])c(OC([H])([H])C(=O)N([H])[H])c([H])c2[H])C([H])([H])[H])c3[H])N([H])[H]	251.19	Active
CHEMBL1965570	O=C(N([H])c4c([H])c([H])c(c2c([H])[s]c1c(c([H])nc(N([H])[H])c12)c3c([H])c([H])oc3[H])c([H])c4[H])N([H])c5c([H])c([H])c([H])c([H])c5[F]	251.19	Active
CHEMBL213505	O=C(N([H])c3c([H])c([H])c(c2noc1nc([H])nc(N([H])[H])c12)c([H])c3[H])N([H])c4c([H])c([H])c([H])c(c4[H])C([F])([F])[F]	251.19	Active
CHEMBL546797	[H]n1c(nc2c([H])c([H])c([H])c([H])c12)C(=NO[H])c4nc3c([H])c([H])c([H])c([H])c3n4[H]	251.19	Active
CHEMBL2007064	[H]n1c(c(c(c1C([H])=C2C(=O)N([H])c3c([H])c([H])c([H])c([H])c23)C([H])([H])[H])C([H])([H])C([H])([H])C(=O)O[H])C([H])([H])[H]	251.19	Active
CHEMBL574738	O=C(N([H])c1c([H])c([H])c(c(c1[H])C([F])([F])[F])C([H])([H])N2C([H])([H])C([H])([H])N(C([H])([H])C([H])([H])[H])C([H])([H])C2([H])[H])N([H])c4c([H])c([H])c(Oc3nc([H])nc(c3[H])N([H])C([H])([H])[H])c([H])c4[H]	316.23	Active
CHEMBL1978099	O=C(N([H])C([H])([H])[H])C([H])([H])Oc3c([H])c([H])c(N([H])c1nc(nc([H])c1[F])N([H])c2c([H])c([H])c(c(c2[H])[S](=O)(=O)N([H])[H])C([H])([H])[H])c([H])c3[H]	316.23	Active
CHEMBL1986143	O=C(N([H])[C@@]([H])(c1c([H])c([H])c([H])c([H])c1[H])C([H])([H])C([H])([H])O[H])c3c([H])c([H])c(c2c([H])c([H])nc([H])c2[H])c([H])c3[H]	316.23	Active
CHEMBL226471	O=C3N([H])C([H])([H])C([H])([H])c4n([H])c(c2c([H])c([H])nc(c1c([H])c([H])c([H])c([H])c1[F])c2[H])c([H])c34	316.23	Active
CHEMBL2006580	[Cl]c3c([H])[s]c2nc([H])nc(N([H])c1c([H])c([H])c(O[H])c([H])c1[H])c23	316.23	Active
CHEMBL1991188	O=C(N([H])[C@@]([H])(c1c([H])c([H])c([H])c(OC([H])([H])[H])c1[H])C([H])([H])[H])c3c([H])c([H])c(c2c([H])c([H])nc([H])c2[F])c([H])c3OC([H])([H])[H]	316.23	Active
CHEMBL1998829	O=C(N([H])c3c([H])nc2n([H])c([H])c(c1c([H])c([H])c([H])c([H])c1[H])c2c3[H])c4c([H])c([H])c([Cl])c([H])c4[H]	316.23	Active
CHEMBL1994526	O=C(N([H])c1c([H])c([H])nc([H])c1[H])[C@@]2([H])C([H])([H])C([H])([H])[C@@]([H])([C@@]([H])(N([H])[H])C([H])([H])[H])C([H])([H])C2([H])[H]	316.23	Active
CHEMBL1964687	O=C(N([H])c1c([H])c(c([H])c(c1[H])C([H])([H])[H])C([H])([H])[H])N([H])c2c([H])c([H])c4c(c2[H])C([H])([H])C([H])([H])c5[s]c3nc([H])nc(N([H])[H])c3c45	316.23	Active
CHEMBL2001149	[H]n1c(nc2c([H])c([H])c(O[H])c([H])c12)c6c([H])c([H])c([S]c5c([H])c([H])c(c4nc3c([H])c([H])c(O[H])c([H])c3n4[H])c([H])c5[H])c([H])c6[H]	316.23	Active

CHEMBL1999718	N#Cc1c([H])c([H])c(nc1[H])N([H])C(=O)N([H])c3c([H])c([H])nc2c([H])c(c([H])c([H])c23)C([F])([F])[F]	316.23	Active
CHEMBL178737	O=C3N([H])C(=O)C(c2c([H])nc1c([H])c([H])c([H])c([H])n12)=C3c5c([H])n7c4c(c([H])c([H])c([H])c45)C([H])([H])N(C(=O)N6C([H])([H])C([H])([H])OC([H])([H])C6([H])[H])C([H])([H])C7([H])[H]	316.23	Active
CHEMBL1997197	[H]C([H])([H])Oc1c([H])c([H])c([H])c([H])c1C([H])([H])N([H])C(=N[H])N([H])c2nc([H])c([H])c(n2)c3[s]c([H])c([H])c3[H]	316.23	Active
CHEMBL2007479	O=C(c1[s]c(nc1N([H])[H])N([H])C([H])([H])C([H])([H])C([H])([H])C([H])([H])[H])c2c([H])c([H])c([H])c([H])c2[H]	316.23	Active
CHEMBL243088	O=C(O[H])c1c([H])c([H])c([H])c([H])c1N([H])c2nc(nc([H])c2[H])N([H])c3c([H])c([H])c([H])c(O[H])c3[H]	316.23	Active
CHEMBL2004615	O=C(c2c([S]C([H])([H])[H])nn(c1nc([H])c([H])c([H])c1[H])c2N([H])[H])N([H])[H]	316.23	Active
CHEMBL482538	[H]n3c2nc([H])c([H])c(c1c([H])c([H])c([H])c([H])c1[H])c2c([H])c3[H]	316.23	Active
CHEMBL1994724	O=C(N([H])[C@]([H])(c1c([H])c([H])c([H])c(OC([H])([H])[H])c1[H])C([H])([H])[H])c2[s]c(c([H])c2[H])c3c([H])c([H])nc([H])c3[H]	316.23	Active
CHEMBL1976240	O=C(N([H])[C@]([H])(c1c([H])c([H])c([H])c(OC([H])([H])[H])c1[H])C([H])([H])N([H])[H])c3c([H])c([H])c(c2c([H])c([H])nc([H])c2[H])c([H])c3[H]	316.23	Active
CHEMBL1973860	[H]OC([H])([H])C([H])([H])N([H])c1nc([H])c3c(c1[H])c([H])c(c2c([H])c([H])[s]c2[H])c([H])c3[H]	316.23	Active
CHEMBL38380	O=[S](=O)(c2c([H])c([H])c([H])c1c([H])nc([H])c([H])c12)N3C([H])([H])C([H])([H])C([H])([H])N([H])C([H])([H])C3([H])[H]	316.23	Active
CHEMBL1990415	O=C(N([H])c3nn([H])c2nc1c([H])c([H])c([H])c([H])c1c([H])c23)c4c([H])c([H])nc([H])c4[H]	316.23	Active
CHEMBL2002099	N#CC([H])([H])[C@@]([H])(n1nc([H])c(c1[H])c3nc([H])nc2n([H])c([H])c([H])c23)[C@@]4([H])C([H])([H])C([H])([H])C([H])([H])C4([H])[H]	316.23	Active
CHEMBL1988153	[F]C([F])([F])c4c([H])c([H])c(Oc2c1c([H])c([s]c1c([H])nc2[H])c3nnc(o3)C([H])([H])[H])c([H])c4[H]	316.23	Active
CHEMBL1974416	O=C(N([H])c3c([H])nc2n([H])c([H])c(c1c([H])c([H])c([H])c([H])c1[H])c2c3[H])c4c([H])c([H])c([H])c([F])c4[F]	316.23	Active
CHEMBL211378	O=C(N([H])c1c([H])c([H])c([H])c(c1[H])C([H])([H])[H])N([H])c4c([H])c([H])c(c3noc2nc([H])nc(N([H])[H])c23)c([H])c4[H]	316.23	Active
CHEMBL2001477	O=C(N([H])[H])c2c([H])c([H])c(c1c([H])c([H])nc([H])c1[H])c([H])c2N([H])C([H])([H])c3c([H])c([H])c([H])c([H])c3[H]	316.23	Active
CHEMBL1973016	O=[S](=O)(c3c([H])c([H])c([H])c(C(=O)N([H])c2nc1c([H])c([H])c([H])c([H])c1n2C([H])([H])C([H])([H])C([H])([H])O[H])c3[H])C([H])([H])[H]	316.23	Active
CHEMBL38380	O=[S](=O)(c2c([H])c([H])c([H])c1c([H])nc([H])c([H])c12)N3C([H])([H])C([H])([H])C([H])([H])N([H])C([H])([H])C3([H])[H]	316.23	Active
CHEMBL2006564	O=C(N(c4c([H])c([H])c(N([H])c3nc(N([H])C1([H])C([H])([H])C1([H])[H])c2nc([H])n([H])c2n3)c([H])c4[H])C([H])([H])C([H])([H])[H])C([H])([H])[H]	398.11	Active
CHEMBL514499	[F]C([F])([F])c1c(c([H])c([H])c([H])c1[H])C([H])([H])Oc2c([H])c([s]c2C(=O)N([H])[H])n4c([H])nc3c([H])c(OC([H])([H])[H])c(OC([H])([H])[H])c([H])c34	398.11	Active

CHEMBL1993781	[H]N([H])[C@]([H])(C([H])([H])OC([H])([H])c1c([H])c([H])c([H])c([H])c1[H])C([H])([H])Oc3c([H])nc([H])c(C([H])=C([H])c2c([H])c([H])nc([H])c2[H])c3[H]	398.11	Active
CHEMBL1967531	O=C(N([H])c3c([H])c([H])c(c2c([H])[s]c1nc([H])nc(N([H])[H])c12)c([H])c3[H])N([H])c4c([H])c([H])c([H])c([H])c4[H]	398.11	Active
CHEMBL1972221	O=[S](=O)(N([H])c3c([H])c([H])c(c2c([H])c([H])c1n([H])nc(N([H])C(=O)C([H])([H])C([H])([H])[H])c1c2[H])c([H])c3[H])C([H])([H])C([H])([H])C([H])([H])[H]	398.11	Active
CHEMBL1984296	O=C(N([H])c1c(c([H])c([H])c(c1[H])C([H])([H])[H])C([H])([H])[H])c3c([H])c(nc2c([H])c([H])c([H])c([H])c23)c4oc([H])c([H])c4[H]	398.11	Active
CHEMBL1996587	[F]c5c([H])c([H])c(c1nc4n(c1c3nn2c(nnc2c([H])c3[H])C(C([H])([H])[H])(C([H])([H])[H])C([H])([H])O[H])C([H])([H])C([H])([H])C4([H])[H])c([F])c5[H]	398.11	Active
CHEMBL2002736	[H]n2nc(N([H])[H])c1c(O[C@]([H])(C([H])([H])OC([H])([H])C([H])([H])[H])C([H])([H])[H])c([H])c([H])c([H])c12	398.11	Active
CHEMBL1984760	O=C(N([H])C([H])([H])C([H])([H])C([H])([H])[H])c3c([H])c([H])c(N([H])c2nc(c1c([H])c([H])n([H])c1n2)C([H])([H])C([H])([H])C([F])([F])[F])c([H])c3[H]	398.11	Active
CHEMBL1991818	[H]C([H])([H])C([H])([H])N3c1c([H])c(N([H])[H])c([H])c([H])c1c2c([H])c([H])c(N([H])[H])c([H])c2[C@]3(O[H])c4c([H])c([H])c([H])c([H])c4[H]	398.11	Active
CHEMBL1994538	O=C(N([H])c1nc([H])c([H])nc1[H])N([H])c3c([H])c([H])nc2c(c([H])c([H])c([H])c23)C([F])([F])[F]	398.11	Active
CHEMBL1996979	O=C(N([H])c3c([H])nc2n([H])c([H])c(c1c([H])c([H])c([H])c([H])c1[H])c2c3[H])c4c([F])c([H])c([H])c([H])c4[F]	398.11	Active
CHEMBL1982506	O=C3c2c(nc([H])c1c([H])c(OC([H])([H])[H])c(OC([H])([H])[H])c([H])c12)C(=O)N3[H]	398.11	Active
CHEMBL1999414	O=C(N([H])C([H])([H])C([H])([H])C([H])([H])N(C([H])([H])C([H])([H])[H])C([H])([H])C([H])([H])[H])c4c([H])nc(N([H])[H])c3c(c2c([H])c([H])c(N([H])C(=O)N([H])c1c([H])c([H])c([H])c(c1[H])C([H])([H])[H])c([H])c2[H])c([H])[s]c34	398.11	Active
CHEMBL2005475	O=C(N([H])C([H])([H])C1([H])C([H])([H])C([H])([H])C([H])([H])C([H])([H])C1([H])[H])c2[s]c3c(c2[H])c(nn3[H])c4c([H])c([H])c([H])c([H])c4[H]	398.11	Active
CHEMBL1997822	O=[S](=O)(c5c([H])c([H])c([H])c(c4c3[s]c([H])c(c2c([H])c1c([H])c(n([H])c1c([H])c2[H])C([H])([H])[H])c3c(nc4[H])N([H])[H])c5[H])C([H])([H])[H]	398.11	Active
CHEMBL1974288	O=C(N([H])[C@@]([H])(c1c([H])c([H])c([H])c([H])c1[H])C([H])([H])O[H])c3c([H])c([H])c(c2c([H])c([H])nc([H])c2[H])c([H])c3[H]	398.11	Active
CHEMBL1968705	O=C(N([H])c6c([H])c([H])c([H])c(c5nc(N([H])c2c([H])c([H])c1n([H])nc([H])c1c2[H])c4c([H])c(OC([H])([H])C([H])([H])N3C([H])([H])C([H])([H])C([H])([H])C3([H])[H])c([H])c([H])c4n5)c6[H])C([H])([H])C([H])([H])C([H])([H])[H]	398.11	Active
CHEMBL1974803	O=c3n([H])c1c([H])c([Cl])c([H])c([Cl])c1c(c2c([H])c([Cl])c([H])c([H])c2O[H])c3O[H]	398.11	Active
CHEMBL1993781	[H]N([H])[C@]([H])(C([H])([H])OC([H])([H])c1c([H])c([H])c([H])c([H])c1[H])C([H])([H])Oc3c([H])nc([H])c(C([H])=C([H])c2c([H])c([H])nc([H])c2[H])c3[H]	398.11	Active
CHEMBL1997597	O=[S](=O)(N([H])c3c([H])c([H])c(c2c([H])c([H])c1n([H])nc(N([H])[H])c1c2[H])c([H])c3[H])C([H])([H])C([H])([H])[H]	398.11	Active
CHEMBL1987009	O=C(N([H])C([H])([H])c1c([H])c([H])c([H])c(OC([H])([H])[H])c1[H])c2[s]c3c(c2[H])c(nn3[H])c4c([H])c([H])c([H])c([H])c4[H]	398.11	Active
CHEMBL124660	O=C(N4C([H])([H])C([H])([H])N(c1nc([H])nc3c1c([H])c(OC([H])([H])[H])c(OC([H])([H])C([H])([H])C([H])([H])N2C([H])([H])C([H])([H])C([H])([H])C([H])([H])C2([H])[H])c3[H])C([H])([H])C4([H])[H])N([H])c5c([H])c([H])c(OC([H])(C([H])([H])[H])C([	398.11	Active

CHEMBL1966087	[H]/C(=C(\[H])c2c([H])nc([H])c(O[C@]1([H])C([H])([H])C([H])([H])N([H])C1([H])[H])c2[H])c3c([H])c([H])nc([H])c3[H]	398.11	Active
CHEMBL1970369	O=C(N([H])[H])[C@]4([H])C([H])([H])C([H])([H])C([H])([H])N(C(=O)c1noc(c1[H])c3c([H])c([H])c2n([H])nc([H])c2c3[H])C4([H])[H]	398.11	Active
CHEMBL2002702	O=C2/C(=C(/[H])c1c(c([H])c(n1[H])C([H])([H])[H])C([H])([H])[H])c3c(N2[H])c([H])c([H])c([H])c3[H]	398.11	Active
CHEMBL262623	[H]N([H])c1nc([H])nc3c1/N=C(\c2c([H])c([H])c([H])c([H])c2[H])C([H])([H])C([H])([H])N3[H]	398.11	Active
CHEMBL296468	O=C(N([H])c2nc([H])c([S]C([H])([H])c1nc([H])c(o1)C(C([H])([H])[H])(C([H])([H])[H])C([H])([H])[H])[s]2)C3([H])C([H])([H])C([H])([H])N([H])C([H])([H])C3([H])[H]	398.11	Active
CHEMBL1984686	[Br]c3c([H])c([H])c2c([H])nc(N([H])c1nc([H])nc([H])c1[H])c([H])c2c3[H]	398.11	Active
CHEMBL1972568	[H]C([H])([H])Oc2c([H])nc([H])c(C([H])=C([H])c1c([H])c([H])nc([H])c1[H])c2[H]	398.11	Active
CHEMBL1987533	O=C(N([H])c2c([H])c([H])c1c([H])nn([H])c1c2[H])C([H])([H])c3c([H])c([H])c([H])c([H])c3[Cl]	398.11	Active
CHEMBL1978167	[Br]c4nc3c([H])c(c1c([H])n(c2c1c([H])c([H])c([H])c2[H])C([H])([H])[H])n([H])c3nc4[H]	398.11	Active
CHEMBL1965589	[F]c5c([H])c([H])c(c2nc1c(nc([H])c([H])n1c2c3nc(nc([H])c3[H])N([H])C([H])([H])C4(O[H])C([H])([H])C4([H])[H])C([H])([H])[H])c([F])c5[H]	3,162.28	Inactive
CHEMBL1991395	[F]c4c([H])c([H])c(c2nc1c([H])nc([H])c([H])n1c2c3nc(nc([H])c3[H])N([H])C([H])([H])C(C([H])([H])[H])(C([H])([H])[H])C([H])([H])O[H])c([H])c4[H]	3,162.28	Inactive
CHEMBL387971	[H]n2nc(c1c([H])c(C#CC([H])([H])OC([H])([H])C([H])([H])OC([H])([H])[H])[s]c1[H])c3c2c4c(C3=O)c([H])c(c([H])c4[H])C([H])([H])N5C([H])([H])C([H])([H])N(C([H])([H])[H])C([H])([H])C5([H])[H]	3,162.28	Inactive
CHEMBL1984788	O=C(N([H])[H])c2c([H])c([Cl])c([H])c1n([H])c(nc12)c3c([H])c([H])c(c([H])c3[F])[C@]4([H])C([H])([H])C([H])([H])C([H])([H])N([H])C4([H])[H]	3,162.28	Inactive
CHEMBL1996923	[Cl]c2c([H])c1nnn([H])c1c([H])c2[Cl]	3,162.28	Inactive
CHEMBL1996473	O=c2n([H])c1c([H])c([H])c([Br])c([H])c1n2c3c([H])c([Cl])c(O[H])c([H])c3O[H]	3,162.28	Inactive
CHEMBL2007097	O=c2nc(N([H])[H])c1c([s]c([H])c1[H])n2[H]	3,162.28	Inactive
CHEMBL2001539	[H]n1nc([H])c3c1c2c([H])c([H])[s]c2C([H])([H])C3([H])[H]	3,162.28	Inactive
CHEMBL2007592	O=c3n([H])c1[s]c5c(c1c2nc(nn23)c4c([H])c([H])c([H])c([H])c4[H])C([H])([H])C([H])([H])C([H])([H])C5([H])[H]	3,162.28	Inactive
CHEMBL2002446	[H]n1nc(c3c1c2c([H])c([H])c(c([H])c2C3([H])[H])C([H])([H])N4C([H])([H])C([H])([H])N(C([H])([H])[H])C([H])([H])C4([H])[H])c6c([H])[s]c(C#CC([H])([H])OC([H])([H])C5([H])C([H])([H])C5([H])[H])c6[H]	3,162.28	Inactive
CHEMBL2005186	O=C(N([H])C([H])(C([H])([H])[H])C([H])([H])[H])C([H])([H])Oc5c([H])c([H])c([H])c(c4nc(N([H])c2c([H])c([H])c1n([H])nc([H])c1c2[H])c3c([H])c([H])c([H])c([H])c3n4)c5[H]	3,162.28	Inactive
CHEMBL1964718	O=C2N([H])N=C1N(C([H])=C(C([H])=C1[H])C([H])([H])[H])C2([H])[H]	3,162.28	Inactive

CHEMBL1980144	O=C(N([H])c1c(c([H])c([H])c([H])c1[H])C([H])([H])[H])N([H])c4c([H])c([H])c(N([H])C(=O)c3c([H])[s]c2nc([H])nc(N([H])[H])c23)c([H])c4[H]	3,162.28	Inactive
CHEMBL1967538	O=c3c2[s]c1n(nc(c1c2nc([H])n3[H])C([H])([H])[H])C([H])([H])[H]	3,162.28	Inactive
CHEMBL2005828	O=C(N([H])c1c([H])c([H])c(c([H])c1[H])C([H])([H])[H])N([H])c4c([H])c([H])c(c3c([H])oc2nc([H])nc(N([H])[H])c23)c([H])c4[H]	3,162.28	Inactive
CHEMBL1966722	O=C(N([H])N([H])C(=[S])N([H])c1c([H])c([H])c([Cl])c([H])c1[H])C(O[H])(c2c([H])c([H])c([H])c([H])c2[H])c3c([H])c([H])c([H])c([H])c3[H]	3,162.28	Inactive
CHEMBL392642	O=[N+]([O-])c1c([H])nc(nc1N([H])C([H])([H])[C@]2([H])C([H])([H])C([H])([H])[C@]([H])(C([H])([H])N([H])[H])C([H])([H])C2([H])[H])N	3,162.28	Inactive
CHEMBL2006188	O=C(N([H])[C@@]([H])(c1c([H])c([H])c([H])c(OC([H])([H])[H])c1[H])C([H])([H])[H])c3c([H])c([H])c(c2c([H])c([H])nc([H])c2[H])c([H])c3N([H])C([H])([H])C([H])([H])C([H])([H])N([H])[H]	3,162.28	Inactive
CHEMBL1995736	O=C(OC([H])([H])C([H])([H])[H])c2c([H])c([H])c([H])c1n([H])c(nc12)c4c([H])c([H])c(N3C([H])([H])C([H])([H])[C@]([H])(N([H])[H])C3([H])[H])c([H])c4[H]	3,162.28	Inactive
CHEMBL483158	[Cl]c3c([H])c([H])c2c1nc(nc([H])c1C([H])([H])N=C(c2c3[H])c4c([F])c([H])c([H])c([H])c4OC([H])([H])[H])N([H])c5c([H])c(OC([H])([H])[H])c(c([H])c5[H])C(=O)O[H]	3,162.28	Inactive
CHEMBL375293	[H]c4c([H])nc([H])c(N([H])c2nc([H])c(c1nc([H])nc([H])c1[H])c(n2)c3oc([H])c([H])c3[H])c4[H]	3,162.28	Inactive
CHEMBL1991800	[F]c5c([H])c([H])c(c2nc1c(nc([H])c([H])n1c2c3nc(nc([H])c3[H])N([H])C([H])([H])C(C([H])([H])[H])(C([H])([H])[H])C([H])([H])O[H])C([H])([H])C4([H])C([H])([H])C4([H])[H])c([H])c5[H]	3,981.07	Inactive
CHEMBL1976093	[F]c4c([H])c([H])c(c2nc1c([H])nc([H])c([H])n1c2c3nc(nc([H])c3[H])N([H])C([H])([H])C(C([H])([H])N(C([H])([H])[H])C([H])([H])[H])(C([H])([H])[H])C([H])([H])[H])c([H])c4[H]	3,981.07	Inactive
CHEMBL1984367	O=C(N([H])C([H])([H])c1c([H])c([H])c(N(C([H])([H])[H])C([H])([H])[H])c([H])c1[H])c2[s]c3c(c2[H])c(nn3[H])c4c([H])c([H])c([H])c([H])c4[H]	3,981.07	Inactive
CHEMBL1965909	O=C4c2c(n([H])c1c([H])c([Cl])c([H])c([H])c1c2=O)C([H])([H])[C@@]([H])(c3c([H])c([H])c([H])c([H])c3[H])C4([H])[H]	3,981.07	Inactive
CHEMBL2007336	O=C(c3c([H])c([H])c([H])c(c2c([H])nc1c([H])c([H])c([H])c([H])c1c2[H])c3[H])C([H])([H])[H]	3,981.07	Inactive
CHEMBL1993243	O=C(N([H])C([H])([H])[H])C([H])=C([H])c4c([H])nc(N([H])[H])c3c(c2c([H])c([H])c1OC([H])([H])Oc1c2[H])c([H])[s]c34	3,981.07	Inactive
CHEMBL1974875	O=C(O[H])c1c([H])[s]c2c1N([H])C([H])([H])C([H])([H])N([H])C2=O	3,981.07	Inactive
CHEMBL1987815	O=C(N([H])[H])c2nc1n(nc([H])c1[H])c(c2[H])c3[s]c([H])c([H])c3[H]	3,981.07	Inactive
CHEMBL1979883	O=C(N([H])c1c([H])c([H])c([H])c(c1[H])C([H])([H])[H])N([H])c4c([H])c([H])c(c3c([H])[s]c2c(C#CC([H])([H])N(C([H])([H])[H])C([H])([H])[H])c([H])nc(N([H])[H])c23)c([H])c4[H]	3,981.07	Inactive
CHEMBL1968515	O=C3N([H])N=C1N(c2c(OC1([H])[H])c([H])c([H])c([H])c2[H])C3([H])[H]	3,981.07	Inactive
CHEMBL1999112	O=C(OC([H])([H])C([H])([H])[H])C3=c2[s]c(=C([H])c1oc([H])c([H])c1[H])c(=O)n2C(N([H])[H])=C(C#N)[C@@]3([H])c4oc([H])c([H])c4[H]	3,981.07	Inactive
CHEMBL1969190	O=C(N([H])c3c([H])c([H])c(c2c([H])c([H])c(OC([H])([H])[H])c1onc(c12)N([H])[H])c([H])c3[H])N([H])c4c([H])c([F])c([H])c([F])c4[H]	3,981.07	Inactive

CHEMBL1981492	O=C(OC([H])([H])[H])c2c(nc1c([H])c([H])c([H])c([H])n12)N([H])[H]	3,981.07	Inactive
CHEMBL1979093	O=C(N([H])c3c([H])c([H])c(c2c([H])c([H])c(OC([H])([H])[H])c1onc(c12)N([H])[H])c([H])c3[H])N([H])c4c([H])c([H])c([F])c(c4[H])C([H])([H])[H]	3,981.07	Inactive
CHEMBL1971649	[F]c1c([H])c([H])c(c([H])c1N([H])C(=O)N([H])c4c([H])c([H])c(c3c([H])c([H])c2nc([H])c([H])nc2c3N([H])[H])c([H])c4[H])C([H])([H])[H]	3,981.07	Inactive
CHEMBL1988622	[H]/C(=C(\[H])c2nn1c(nc([H])c1[H])c([H])c2[H])c3c([H])c([H])c([H])c([H])c3[H]	3,981.07	Inactive
CHEMBL1991782	O=C1N([H])C([H])([H])c2c1c([H])c([H])c([H])c2c3[s]c([H])c([H])c3[H]	3,981.07	Inactive
CHEMBL1990288	[F]c5c([H])c([H])c(c2nc1oc([H])c([H])n1c2c3nc(nc([H])c3[H])N([H])[C@]([H])(c4c([H])c([H])c([H])c([H])c4[H])C([H])([H])[H])c([H])c5[H]	3,981.07	Inactive
CHEMBL31	[F]c1c(c(OC([H])([H])[H])c2c(c1[H])c(=O)c(C(=O)O[H])c([H])n2[C@@]3([H])C([H])([H])C3([H])[H])N4C([H])([H])C([H])([H])N([H])[C@]([H])(C([H])([H])[H])C4([H])[H]	3,981.07	Inactive
CHEMBL1973145	O=C(N([H])c1c([H])c([H])c([H])c(c1[H])C([H])([H])[H])N([H])c4c([H])c([H])c(c3c([H])[s]c2nc(nc(N([H])[H])c23)N([H])[H])c([H])c4[H]	3,981.07	Inactive
CHEMBL1971694	[O-][s+]1nc(N([H])C([H])([H])C([H])([H])C([H])([H])C([H])([H])[H])c(n1)N([H])c2c([H])c([H])c([F])c([H])c2[H]	3,981.07	Inactive
CHEMBL316264	[F]c4c([H])c([H])c(c3nc(c1c([H])c([H])c([F])c([H])c1[H])c(c2c([H])c([H])nc([H])c2[H])n3[H])c([H])c4[H]	3,981.07	Inactive
CHEMBL1972583	O=C(N([H])[H])c3c([H])nn2c(c1c([H])c([H])c([H])c([H])c1[H])c([H])c([H])nc23	3,981.07	Inactive
CHEMBL1972937	O=C2C([S]/C(=N\c1c([H])c([H])c([H])c([H])c1[H])N2[H])=C([H])c3c([H])c([H])c([H])c(OC([H])([H])[H])c3[H]	3,981.07	Inactive
CHEMBL1980163	[H]c1c([H])c([H])c(c([H])c1[H])C([H])([H])Oc4c([H])c([H])c(N([H])c3nc([H])nc2c([H])c([H])c([H])c([H])c23)c([H])c4[H]	5,011.87	Inactive
CHEMBL1972584	O=C(N7C([H])([H])C([H])([H])N([C@@]6([H])C([H])([H])C([H])([H])[C@@]([H])(n4nc(c3c([H])c([H])c(N([H])C(=O)c2c([H])c1c([H])c([H])c([H])c([H])c1n2C([H])([H])[H])c([H])c3[H])c5c(nc([H])nc45)N([H])[H])C([H])([H])C6([H])[H])C([H])([H])C7([H]	5,011.87	Inactive
CHEMBL1976872	O=C(N([H])c1nn2c(c1[H])c([H])c([H])c([H])c2[H])C([H])([H])[H]	5,011.87	Inactive
CHEMBL1999120	O=c1c([H])c(c2c(n1[H])n([H])n([H])c2=O)C([H])(C([H])([H])[H])C([H])([H])[H]	5,011.87	Inactive
CHEMBL2005214	[H]n1nc(c2c1O/C(=C(/C#N)[C@]2([H])c3c([H])c([H])c([H])c([H])c3[Br])N([H])[H])C([H])([H])[H]	5,011.87	Inactive
CHEMBL1972584	O=C(N7C([H])([H])C([H])([H])N([C@@]6([H])C([H])([H])C([H])([H])[C@@]([H])(n4nc(c3c([H])c([H])c(N([H])C(=O)c2c([H])c1c([H])c([H])c([H])c([H])c1n2C([H])([H])[H])c([H])c3[H])c5c(nc([H])nc45)N([H])[H])C([H])([H])C6([H])[H])C([H])([H])C7([H]	5,011.87	Inactive
CHEMBL1979577	O=C(N([H])c1c([H])c([H])c([H])c(c1[H])C([H])([H])[H])N([H])c4c([H])c([H])c(c3c([H])[s]c2c(C#CC(N([H])[H])(C([H])([H])[H])C([H])([H])[H])c([H])nc(N([H])[H])c23)c([H])c4[H]	5,011.87	Inactive
CHEMBL1994159	O=C(c3c([H])c([H])c([H])c(c2nn1c(nc([H])c1[H])c([H])c2[H])c3[H])C([H])([H])[H]	5,011.87	Inactive
CHEMBL1983195	O=C(N(C([H])([H])C([H])([H])[H])C([H])([H])C([H])([H])[H])N([H])c3c([H])c([H])c2nc(c1oc([H])c([H])c1[H])c(nc2c3[H])c4oc([H])c([H])c4[H]	5,011.87	Inactive

CHEMBL1980376	[H]C([H])([H])c3c([H])c([H])c([H])c(c2c([H])c([H])c([H])c1onc(N([H])[H])c12)c3[H]	5,011.87	Inactive
CHEMBL1973399	O=C(N([H])[H])c1c([H])[s]c2c1N([H])C([H])([H])C([H])([H])C2=O	5,011.87	Inactive
CHEMBL2002240	O=C(N([H])c1nc(c(C#N)c(c1[H])N([H])[H])c3c([H])c([H])c2c([H])c([H])c([H])c([H])c2c3[H])C([H])([H])[H]	5,011.87	Inactive
CHEMBL1965423	[F]c5c([H])c([H])c(c2nc1[s]c([H])c([H])n1c2c4c([H])c([H])c3nc(n(c3c4[H])C([H])([H])C([H])(C([H])([H])[H])C([H])([H])[H])N([H])[H])c([H])c5[H]	5,011.87	Inactive
CHEMBL1970950	O=C(O[H])c1c([H])c([H])c([H])c([H])c1N([H])c2nc(nc([H])c2[H])N([H])C([H])([H])c3c([H])c([H])c([H])c(O[H])c3[H]	5,011.87	Inactive
CHEMBL1972576	O=C(N([H])c1c([H])c([H])c(c([H])c1[H])C([H])([H])[H])N([H])c4c([H])c([H])c(N([H])C(=O)c3c([H])[s]c2nc([H])nc(N([H])[H])c23)c([H])c4[H]	5,011.87	Inactive
CHEMBL1984586	O=C(N([H])c1c(c([H])c([H])c([H])c1[H])C([H])([H])[H])N([H])c4c([H])c([H])c(c3c([H])oc2nc([H])nc(N([H])[H])c23)c([H])c4[H]	5,011.87	Inactive
CHEMBL1991867	[H]n2nc(N([H])[H])c1[s]c([Cl])nc12	5,011.87	Inactive
CHEMBL1966279	O=[S](=O)(N([H])C([H])([H])C([H])([H])C([H])([H])N([H])[H])c4c([H])c([H])c([H])c(C(=O)N([H])c3c([H])c([H])c(c2nn([H])c(=O)c1c([H])c([H])c([H])c([H])c12)c([H])c3[H])c4[H]	5,011.87	Inactive
CHEMBL1988000	O=C(N=c1[s]c(c(n1C([H])([H])C([H])([H])OC([H])([H])[H])C([H])([H])[H])C([H])([H])[H])c2c([H])c([S]C([H])([H])[H])c([H])c([H])c2[Cl]	5,011.87	Inactive
CHEMBL1965988	O=C(N([H])c1c([H])c([H])c([H])c(c1[H])C([H])([H])[H])N([H])c4c([H])c([H])c(c3c([H])[s]c2c(C#CC([H])([H])N([H])[H])c([H])nc(N([H])[H])c23)c([H])c4[H]	5,011.87	Inactive
CHEMBL1982465	O=C(N([H])c1c([H])c([H])c([H])c(c1[H])C([H])([H])[H])N([H])c5c([H])c([H])c(c4c([H])[s]c3c(C#CC([H])([H])N2C([H])([H])C([H])([H])OC([H])([H])C2([H])[H])c([H])nc(N([H])[H])c34)c([H])c5[H]	6,309.57	Inactive
CHEMBL2002165	O=C(N([H])c4c([H])c([H])c(c3c2c(nc([H])c(c1c([H])n(nc1[H])C([H])([H])[C@@]([H])(O[H])C([H])([H])[H])c2[s]c3[H])N([H])[H])c([H])c4[H])N([H])c5c([H])c([H])c([H])c([F])c5[H]	6,309.57	Inactive
CHEMBL1986328	O=C(N(C([H])([H])[H])C([H])([H])[H])N([H])c3c([H])c([H])c2nc(c1oc([H])c([H])c1[H])c(nc2c3[H])c4oc([H])c([H])c4[H]	6,309.57	Inactive
CHEMBL1975815	O=C4c2c(n([H])c1c([H])c([Cl])c([H])c([H])c1c2=O)C([H])([H])[C@@]([H])(c3c([H])c([H])c([F])c([H])c3[H])C4([H])[H]	6,309.57	Inactive
CHEMBL1983630	O=C2N([H])c1nc([H])c(c([H])c1C2([H])[H])c3c([H])c([H])c([H])c([H])c3[H]	6,309.57	Inactive
CHEMBL1990884	O=C(c4c([H])c([H])c([H])c(c3c([H])c([H])c(N([H])c2oc1c(c([H])c(c([H])c1n2)C([H])([H])[H])C([H])([H])[H])c([F])c3[H])c4N([H])[H])N([H])[H]	6,309.57	Inactive
CHEMBL17370	[F]c4c([H])c([H])c(c2nc(c1c([H])c([H])c([S]C([H])([H])[H])c([H])c1[H])n([H])c2c3c([H])c([H])nc([H])c3[H])c([H])c4[H]	6,309.57	Inactive
CHEMBL1973937	O=C(N([H])c3c([H])c([H])c(c1c([H])c([H])c(c2c1c(nn2[H])N([H])[H])C([H])([H])[H])c([H])c3[H])N([H])c4c([H])c([H])c([H])c([Br])c4[H]	6,309.57	Inactive
CHEMBL1984500	O=C1N([H])C([H])([H])c2c([Br])c([H])c([H])c([H])c12	6,309.57	Inactive
CHEMBL1978200	[F]c4c([H])c([H])c(c2nc1c(nc([H])c([H])n1c2c3nc(nc([H])c3[H])N([H])C([H])([H])C([H])([H])C(O[H])(C([H])([H])[H])C([H])([H])[H])C([H])([H])[H])c([F])c4[H]	6,309.57	Inactive

CHEMBL1989569	O=C(c3c2nc(c1c([H])c([H])c([H])c(OC([H])([H])C([H])([H])N(C([H])([H])[H])C([H])([H])[H])c1[H])n([H])c2c([H])c([H])c3[H])N([H])[H]	6,309.57	Inactive
CHEMBL2003456	O=c3n([H])c2c([H])c(c1c([H])c([H])c([H])c([H])c1[H])c([H])nc2n3[H]	6,309.57	Inactive
CHEMBL1967720	O=C(N([H])[H])C(n1nc([H])c(c1[H])c5c([H])nc(N([H])[H])c4c(c3c([H])c([H])c(N([H])C(=O)N([H])c2c([H])c([H])c([H])c([F])c2[H])c([H])c3[H])c([H])[s]c45)(C([H])([H])[H])C([H])([H])[H]	6,309.57	Inactive
CHEMBL1990432	O=C4c2c(n([H])c1c([H])c([Cl])c([H])c([H])c1c2=O)C([H])([H])[C@@]([H])(c3c([H])c([H])c([Cl])c([H])c3[H])C4([H])[H]	6,309.57	Inactive
CHEMBL1997872	O=C(c3c([H])c([H])c([H])c(c2c([H])nc1n([H])c([H])c([H])c1c2[H])c3[H])N4C([H])([H])C([H])([H])OC([H])([H])C4([H])[H]	6,309.57	Inactive
CHEMBL354676	O=C(N([H])c1c([H])c([H])c([H])c([H])c1[H])N([H])c2c([H])c([H])c([H])c([H])c2[H]	7,943.28	Inactive
CHEMBL2004311	O=C(N([H])c1c([H])c(c([H])c(c1[H])C([H])([H])[H])C([H])([H])[H])N([H])c4c([H])c([H])c(c2c([H])c([H])c([H])c3c2C([H])([H])N([H])C3=O)c(c4[H])C([H])([H])[H]	7,943.28	Inactive
CHEMBL1982874	[F]c6c([H])c([H])c(c2nc1[s]c([H])c([H])n1c2c4c([H])c([H])c3nc(N([H])[H])n(c3c4[H])C([H])([H])C5([H])C([H])([H])C5([H])[H])c([H])c6[H]	7,943.28	Inactive
CHEMBL1984402	O=C(c1c(nn(c(=O)c1N([H])[H])C([H])([H])[H])c2c([H])c([H])c([H])c([H])c2[H])C([H])([H])[H]	7,943.28	Inactive
CHEMBL1975256	O=C(c2nnn(c1nonc1N([H])[H])c2c3[s]c([H])c([H])c3[H])N([H])N=C([H])c4[s]c([H])c([H])c4[H]	7,943.28	Inactive
CHEMBL1999778	N#Cc1c(nc(OC([H])([H])[H])c(C#N)c1c2c([H])c([H])c([Cl])c([H])c2[Cl])N([H])[H]	7,943.28	Inactive
CHEMBL1964644	O=C(N([H])C([H])([H])c1c([H])c([H])c([H])c([H])c1[H])N([H])c2nc([H])c([s]2)[N+](=O)[O-]	7,943.28	Inactive
CHEMBL1983025	O=C(N([H])c3c([H])c([H])c(c2c1c(nc([H])c(C#CC(N([H])[H])(C([H])([H])C([H])([H])[H])C([H])([H])C([H])([H])[H])c1[s]c2[H])N([H])[H])c([H])c3[H])N([H])c4c([H])c([H])c([H])c(c4[H])C([H])([H])[H]	7,943.28	Inactive
CHEMBL491758	[F]c1c([H])nc(nc1N([H])c2c([H])c([H])c([H])c([H])c2[H])N([H])c3c([H])c([H])c([H])c([H])c3[H]	7,943.28	Inactive
CHEMBL1968926	[H]n1nc(nc1[S]C([H])([H])c2c([H])c([H])c([H])c([H])c2[H])c3c([H])c([H])nc([H])c3[H]	7,943.28	Inactive
CHEMBL2002450	[H]C([H])([H])Oc3c([H])c([H])c(c1c([H])c([H])c(N([H])[H])c([H])c1[H])c2c([H])noc23	7,943.28	Inactive
CHEMBL1984162	[H]C([H])([H])OC([H])([H])C([H])([H])O[C@]6([H])C([H])([H])C([H])([H])[C@]([H])(n4nc(c3c([H])c([H])c(N([H])c2nc1c([H])c(c([H])c(c1o2)C([H])([H])[H])C([H])([H])[H])c([H])c3[H])c5c(nc([H])nc45)N([H])[H])C([H])([H])C6([H])[H]	7,943.28	Inactive
CHEMBL1971245	O=C(c2nc1c([H])c([H])c([H])c([H])n1c2c3nc(nc([H])c3[H])N([H])c5c([H])c([H])c([H])c(N4C([H])([H])C([H])([H])N(C([H])([H])[H])C([H])([H])C4([H])[H])c5[H])N([H])[H]	7,943.28	Inactive
CHEMBL1983884	[H]N([H])c3nc([H])nc2nc1c([H])c([H])c([H])c([H])c1n23	7,943.28	Inactive
CHEMBL1970189	[H]n1nc(c2c1O/C(=C(/C#N)[C@]2([H])c3c([H])c([H])c([H])c([H])c3[Cl])N([H])[H])C([H])([H])[H]	7,943.28	Inactive
CHEMBL1968590	O=C(N([H])C([H])([H])[H])C([H])=C([H])c4c([H])nc(N([H])[H])c3c(c2c([H])c([H])c1[s]c(nc1c2[H])C([H])([H])[H])c([H])[s]c34	7,943.28	Inactive

CHEMBL1973211	O=C(N([H])c1c([H])c([H])c([H])c(c1[H])C([H])([H])[H])N([H])c5c([H])c([H])c(c4c([H])[s]c3c(C#CC([H])([H])N2C([H])([H])C([H])([H])C([H])([H])C2([H])[H])c([H])nc(N([H])[H])c34)c([H])c5[H]	7,943.28	Inactive
CHEMBL117217	O=C4c2c(nc1c([H])c([H])c([Cl])c([H])c1c2O[H])C([H])([H])[C@@]([H])(c3c([H])c([H])c([Cl])c([H])c3[Cl])C4([H])[H]	7,943.28	Inactive
CHEMBL354676	O=C(N([H])c1c([H])c([H])c([H])c([H])c1[H])N([H])c2c([H])c([H])c([H])c([H])c2[H]	7,943.28	Inactive
CHEMBL1980704	[H]C([H])([H])c5c([H])c3nnc([S]C([H])([H])c1nc2n(c1[H])c([H])c([H])c([H])c2[H])n3c4c([H])c([H])c([H])c([H])c45	7,943.28	Inactive
CHEMBL1393571	N#Cc3c([H])nn2c(c1c([H])c([H])c([H])c([H])c1[H])c([H])c([H])nc23	10,000	Inactive
CHEMBL1985042	O=c1c([H])c(c3c(n1[H])n([H])n(c2c([H])c([H])c([H])c([H])c2[H])c3=O)C([H])([H])[H]	10,000	Inactive
CHEMBL1986781	O=C(N5C([H])([H])C([H])([H])C([H])(N([H])c1nc([H])c([H])c(n1)c3c(nc2oc([H])c([H])n23)c4c([H])c([H])c([F])c([H])c4[H])C([H])([H])C5([H])[H])C([H])([H])[H]	10,000	Inactive
CHEMBL2002182	O=c3n([H])nc2c(c1c([H])c([H])c([H])c([H])c1[H])c([H])c([H])c([H])n23	10,000	Inactive
CHEMBL272453	O=C(N([H])c1c([H])c([H])c(OC([H])([H])[H])c([Cl])c1[H])N([H])c4c([H])c([H])c(c3c([H])c([H])c([H])c2onc(N([H])[H])c23)c([H])c4[H]	10,000	Inactive
CHEMBL1682558	[F]c5c([H])c([H])c(c1nc([H])n(c1c3c([H])c([H])c2n([H])nc([H])c2c3[H])C([H])([H])C([H])([H])N4C([H])([H])C([H])([H])OC([H])([H])C4([H])[H])c([H])c5[H]	10,000	Inactive
CHEMBL208637	O=C(N([H])c1nc(OC([H])([H])C([H])([H])[H])c(C#N)c(c1[H])N([H])[H])C([H])([H])[H]	10,000	Inactive
CHEMBL1972820	O=C(O[H])c3c([H])c([H])c(N2C(=O)c1c([H])c([H])c([H])c([H])c1C2=O)c([H])c3O[H]	10,000	Inactive
CHEMBL1970806	O=c3n([H])c1[s]c5c(c1c2nc(nn23)c4c([H])c([H])c([H])nc4[H])C([H])([H])C([H])([H])C([H])([H])C5([H])[H]	10,000	Inactive
CHEMBL1972849	[F]c5c([H])c([H])c(c2nc1oc([H])c([H])n1c2c4nn3c(nnc3c([H])c4[H])C(C([H])([H])[H])(C([H])([H])[H])C([H])([H])O[H])c([F])c5[H]	10,000	Inactive
CHEMBL1970142	O=C(N([H])C([H])([H])[H])[C@@]([H])(n1nc([H])c(c1[H])c5c([H])nc(N([H])[H])c4c(c3c([H])c([H])c(N([H])C(=O)N([H])c2c([H])c([H])c(c([H])c2[H])C([H])([H])[H])c([H])c3[H])c([H])[s]c45)C([H])([H])[H]	10,000	Inactive
CHEMBL1992306	O=C(N([H])C([H])([H])[H])c3nc([H])c([H])c(Oc2c([H])c([H])c(N([H])C(=O)N([H])c1c([H])c([H])c([H])c(c1[H])C([F])([F])[F])c([H])c2[H])c3[H]	10,000	Inactive
CHEMBL184847	O=C(N([H])c1c([H])c([H])c(c(c1[H])C([H])([H])[H])C([H])([H])[H])N([H])c4c([H])c([H])c(c2c([H])c([H])c([H])c3c2C([H])([H])N([H])C3=O)c([H])c4[H]	10,000	Inactive
CHEMBL1989474	[F]c3c([H])c([H])c(c1nn(c([H])c1c2nc([H])nc([H])c2[H])C([H])([H])[H])c([H])c3[H]	10,000	Inactive
CHEMBL2004447	[H]n1nc(c2c1O/C(=C(/C#N)[C@]2([H])c3c([H])c([H])c([H])c([H])c3[F])N([H])[H])C(C([H])([H])[H])(C([H])([H])[H])C([H])([H])[H]	10,000	Inactive
CHEMBL95692	[F]c4c([H])c([H])c(c1nc([H])n(c1c2nc(OC([H])([H])[H])nc([H])c2[H])C3([H])C([H])([H])C([H])([H])N([H])C([H])([H])C3([H])[H])c([H])c4[H]	10,000	Inactive
CHEMBL210928	O=C(N([H])c1nc(OC([H])([H])C([H])([H])[H])c([Cl])c(c1[H])N([H])[H])C([H])([H])c2c([H])c(OC([H])([H])[H])c(c([H])c2OC([H])([H])[H])[S](=O)(=O)C([H])([H])[H]	10,000	Inactive

CHEMBL1989708	O=C(N([H])c1c([H])c([H])c([H])c(c1[H])C([H])([H])[H])N([H])c5c([H])c([H])c(c4c([H])[s]c3c(c2c([H])nn(c2[H])C([H])([H])[C@@]([H])(O[H])C([H])([H])[H])c([H])nc(N([H])[H])c34)c([H])c5[H]	10,000	Inactive
CHEMBL1975121	O=C(N([H])[C@]([H])(c1c([H])c([H])c([H])c(OC([H])([H])[H])c1[H])C([H])([H])[H])c3c([H])c([H])c(c2c([H])c([H])nc([H])c2[H])c([H])c3[H]	10,000	Inactive
CHEMBL1976732	[F]c5c([H])c([H])c(c2nc1oc([H])c([H])n1c2c4nn3c(nnc3c([H])c4[H])C([H])([H])C(O[H])(C([H])([H])[H])C([H])([H])[H])c([F])c5[H]	10,000	Inactive
CHEMBL1967560	O=C2N(N(c1nc(nc([H])c1N2C([H])([H])[H])N([H])c4c([H])c([H])c(C(=O)N([H])[C@]3([H])C([H])([H])C([H])([H])N(C([H])([H])[H])C([H])([H])C3([H])[H])c([H])c4OC([H])([H])[H])[C@@]5([H])C([H])([H])C([H])([H])C([H])([H])C5([H])[H])C([H])([H])C(	10,000	Inactive
CHEMBL2000801	O=C(N([H])c3c([H])c([H])c(c2c([H])c([H])c([H])c1[s]nc([H])c12)c([H])c3[H])N([H])c4c([H])c([H])c([F])c(c4[H])C([F])([F])[F]	10,000	Inactive
CHEMBL1972142	O=C(N([H])C([H])([H])c1c([H])c([H])c([H])c([H])c1[H])c4c([H])c([H])c([H])c(c3c([H])nc2n([H])c([H])c([H])c2c3[H])c4[H]	10,000	Inactive
CHEMBL1825138	[Cl]c1c(c([Cl])c([F])c([H])c1[H])[C@]([H])(Oc4c([H])c(c2c([H])nn(c2[H])[C@@]3([H])C([H])([H])C([H])([H])N([H])C([H])([H])C3([H])[H])c([H])nc4N([H])[H])C([H])([H])[H]	10,000	Inactive
CHEMBL1982361	O=c3c(N([H])C([H])([H])c1c([H])c([H])c([H])c([H])c1[F])c(N([H])c2c([H])c([H])nc([H])c2[H])c3=O	10,000	Inactive
CHEMBL539474	O=C1N(C([H])([H])C([H])([H])C1([H])[H])C([H])([H])c2c([H])c([H])c3c(c2[H])n([H])c(=O)c4c([H])c([H])c([H])n34	10,000	Inactive
CHEMBL1624529	[F]c1c(nc2c(c1[H])c(=O)c(C(=O)O[H])c([H])n2c3c([H])c([H])c([F])c([H])c3[F])N5C([H])([H])[C@@]4([H])[C@]([H])(N([H])[H])[C@@]4([H])C5([H])[H]	10,000	Inactive
CHEMBL1969879	O=C(N3C([H])([H])C([H])([H])[C@@]([H])(C([H])([H])[H])[C@@]([H])(N(c2nc([H])nc1n([H])c([H])c([H])c12)C([H])([H])[H])C3([H])[H])C([H])([H])O[H]	10,000	Inactive
CHEMBL1971021	O=C(N([H])c1c([H])c(c([H])c(c1[H])C([H])([H])[H])C([H])([H])[H])N([H])c4c([H])c([H])c(c2c([H])c([H])c([H])c3c2C([H])([H])N([H])C3=O)c(c4[H])C([F])([F])[F]	10,000	Inactive
CHEMBL1984274	O=C3/C(=N/N([H])c2c([H])c([H])c([H])c1c(=O)n([H])n([H])c(=O)c12)C(=NN3c4c([H])c([H])c([H])c([H])c4[H])c5c([H])c([H])c([H])c([H])c5[H]	10,000	Inactive
CHEMBL1975233	[Cl]c1c(c([H])c(OC([H])([H])[H])c(c1[H])N([H])C(=O)N([H])c2nc([H])c(nc2[H])C#N)C([H])([H])[H]	10,000	Inactive
CHEMBL1990821	O=C(O[H])c1c(c3c(n1[H])c(c2c([H])c([H])c([H])c([H])c2C([H])([H])[H])c([H])c([H])c3[H])C([H])([H])C([H])([H])C([H])([H])Oc4c(c(c([H])c(c4[H])C([H])([H])[H])C([H])([H])[H])C([H])([H])[H]	10,000	Inactive
CHEMBL377383	O=C(c1nc(OC([H])([H])C([H])([H])[H])c([Cl])c(c1[H])N([H])[H])N([H])C([H])([H])c2c([H])c([H])c(c([H])c2[H])[S](=O)(=O)C([H])([H])[H]	10,000	Inactive
CHEMBL1977604	O=C(O[H])c2c1[S]C([H])([H])[C@]([H])(n1c4c(c2=O)c([H])c([F])c(N3C([H])([H])C([H])([H])[C@]([H])(N([H])[H])C3([H])[H])c4[F])C([H])([H])[H]	10,000	Inactive
CHEMBL1419458	O=C(c2nnn(c1nonc1N([H])[H])c2c3c([H])c([H])c([H])c([H])c3[H])N([H])N=C([H])c4c([H])c([H])nc([H])c4[H]	10,000	Inactive
CHEMBL256835	[F]c4c([H])c([H])c(c1nc([H])n(c1c2nc(OC([H])([H])[H])nc([H])c2[H])[C@]3([H])C([H])([H])C([H])([H])[C@]([H])(O[H])C([H])([H])C3([H])[H])c([H])c4[H]	10,000	Inactive
CHEMBL1984039	O=C(O[H])c3c(c2c([H])c([H])c([H])c(c1c(nn(c1C([H])([H])[H])C([H])([H])[H])C([H])([H])[H])c2n3[H])C([H])([H])C([H])([H])C([H])([H])Oc5c([H])c([H])c([H])c4c([H])c([H])c([H])c([H])c45	10,000	Inactive
CHEMBL1979318	[H]n1nc(c2c1O/C(=C(/C#N)[C@]2([H])c3c([H])c([H])c([H])c([H])c3[I])N([H])[H])C([H])([H])[H]	10,000	Inactive

CHEMBL127898	[F]C([F])([F])Oc4c([H])c([H])c(c1c(n(c(=O)c(C#N)c1[H])C([H])([H])[H])C([H])([H])O[C@]([H])(c2n(c([H])nc2[H])C([H])([H])[H])c3c([H])c([H])c(C#N)c([H])c3[H])c([H])c4[H]	10,000	Inactive
CHEMBL1984700	[F]c4c([H])c([H])c(c2nc1c(OC([H])(C([H])([H])[H])C([H])([H])[H])nc([H])c([H])n1c2c3nc(nc([H])c3[H])N([H])C([H])([H])C(O[H])(C([H])([H])[H])C([H])([H])[H])c([F])c4[H]	10,000	Inactive
CHEMBL1969126	[F]c5c([H])c([H])c(c2nc1oc([H])c([H])n1c2c4c([H])c([H])c3nnn(c3c4[H])C([H])(C([H])([H])[H])C([H])([H])[H])c([F])c5[H]	10,000	Inactive
CHEMBL2006493	O=C3c2c(oc1c([H])c(c([H])c([H])c1c2=O)C([F])([F])[F])C([H])([H])C([H])([H])C3([H])[H]	10,000	Inactive
CHEMBL1987793	O=C(O[H])c2c(c1c(c([H])c([H])c([H])c1[H])C([H])([H])[H])c5c(n2C([H])([H])C([H])([H])C([H])([H])Oc4c([H])c([H])c([H])c3c([H])c([H])c([H])c([H])c34)c([H])c([H])c([H])c5[H]	10,000	Inactive
CHEMBL1988717	O=C(N([H])c4c([H])c([H])c(c3c(n2nc([H])c(c1c([H])n(nc1[H])C([H])([H])[H])c2nc3[H])N([H])[H])c([H])c4[H])N([H])c5c([H])c([H])c([H])c(c5[H])C([F])([F])[F]	10,000	Inactive
CHEMBL223367	N#Cc1nc([H])c3nc1OC([H])([H])C([H])([H])C([H])([H])C([H])([H])C([H])([H])Oc2c([H])c(c([Cl])c([H])c2N([H])C(=O)N3[H])C([H])([H])C([H])([H])C([H])([H])O[H]	10,000	Inactive
CHEMBL2006631	O=[S](=O)(N(C([H])([H])[H])C([H])([H])[H])c1c([H])c([H])c([H])c([H])c1c2c([H])c([H])c4c(c2[H])N([H])c3c([H])c([H])c([H])c([H])c3N([H])C4=O	10,000	Inactive
CHEMBL1982466	O=C(N([H])c1c([H])c([H])c(c([H])c1[H])C([H])([H])[H])N([H])c5c([H])c([H])c(c4c([H])[s]c3c(c2c([H])nn(c2[H])C([H])([H])[H])c([H])nc(N([H])[H])c34)c([H])c5[H]	10,000	Inactive
CHEMBL1988075	O=C(N([H])c4c([H])c([H])c(c3c2c(nc([H])c(c1c([H])c([H])c(nc1[H])C([H])([H])[H])c2[s]c3[H])N([H])[H])c([H])c4[H])N([H])c5c([H])c([H])c([H])c([F])c5[H]	10,000	Inactive
CHEMBL1970203	O=C(N([H])c1c([H])c(c([H])c(c1[H])C([H])([H])[H])C([H])([H])[H])N([H])c4c([H])c([H])c(N([H])C(=O)c3c([H])[s]c2nc([H])nc(N([H])[H])c23)c([H])c4[H]	10,000	Inactive
CHEMBL1987910	O=C(N([H])c3c([H])c([H])c(c2c([H])[s]c1c([H])c([H])nc(N([H])[H])c12)c([H])c3[H])N([H])c4c([H])c([H])c([H])c([H])c4C([F])([F])[F]	10,000	Inactive
CHEMBL1992555	O=C(N([H])N=C([H])c3oc([S]c2nc1c([H])c([H])c([H])c([H])c1n2[H])c([H])c3[H])c4c([H])c([H])c([H])c([N+](=O)[O-])c4[H]	10,000	Inactive
CHEMBL2001224	O=C(N([H])c2c(OC1([H])C([H])([H])C([H])([H])C([H])([H])C1([H])[H])c([H])c(c([Cl])c2[H])C([H])([H])[H])N([H])c3nc([H])c(nc3[H])C#N	10,000	Inactive
CHEMBL1998551	[F]c5c([H])c([H])c(c1nc4n(c1c3nn2c(nnc2c([H])c3[H])C(O[H])(C([H])([H])[H])C([H])([H])[H])C([H])([H])C([H])([H])C4([H])[H])c([F])c5[H]	10,000	Inactive
CHEMBL249282	N#Cc1nc([H])c(c([H])c1[H])c2nn([H])c4c2C([H])([H])c5c([H])c([H])c(OC([H])([H])C([H])([H])N3C([H])([H])C([H])([H])OC([H])([H])C3([H])[H])c([H])c45	10,000	Inactive
CHEMBL1975357	O=C(OC(C([H])([H])[H])(C([H])([H])[H])C([H])([H])[H])n3c([H])c(c1c([H])nc([H])n1C2([H])C([H])([H])C([H])([H])C([H])([H])C([H])([H])C2([H])[H])c4c([H])c([H])c([H])c([H])c34	10,000	Inactive
CHEMBL2000408	[F]c4c([H])c([H])c(c2nc1n(c([H])c(nc1C([H])([H])[H])C([H])([H])[H])c2c3nc(nc([H])c3[H])N([H])C([H])([H])C(C([H])([H])[H])(C([H])([H])[H])C([H])([H])O[H])c([F])c4[H]	10,000	Inactive
CHEMBL2001257	O=C(N([H])c3c([H])c([H])c(c2c1c(nc([H])c(C#CC([H])([H])N(C([H])([H])C([H])([H])[H])C([H])([H])C([H])([H])[H])c1[s]c2[H])N([H])[H])c([H])c3[H])N([H])c4c([H])c([H])c([H])c(c4[H])C([H])([H])[H]	10,000	Inactive
CHEMBL2006715	O=C(N([H])c6c([H])c([H])c([H])c(c2nc1[s]c([H])c([H])n1c2c3nc(nc([H])c3[H])N([H])c5c([H])c([H])c([H])c(N4C(=O)C([H])([H])C([H])([H])C4([H])[H])c5[H])c6[H])c7c([F])c([H])c([H])c([H])c7[F]	10,000	Inactive
CHEMBL1972183	[H]C([H])([H])N1C([H])([H])C([H])([H])C([H])([H])[C@@]1([H])C([H])([H])Oc2c([H])nc([H])c(c2[H])C([H])([H])C([H])([H])c3c([H])c([H])c([H])c([H])c3[H]	10,000	Inactive

CHEMBL1974254	O=C(N([H])c1c([H])c([H])c(OC([H])([H])C([H])([H])[H])c([H])c1[H])N([H])c5c([H])c([H])c(c4c([H])[s]c3c(c2c([H])nn(c2[H])C([H])([H])[H])c([H])nc(N([H])[H])c34)c([H])c5[H]	10,000	Inactive
CHEMBL378627	O=[S](=O)(c1c([H])c(OC([H])([H])[H])c(c([H])c1OC([H])([H])[H])C([H])([H])C(=O)N([H])c2nc(OC([H])(C([H])([H])[H])C([H])([H])[H])c(C#N)c(c2[H])N([H])[H])C([H])([H])[H]	10,000	Inactive
CHEMBL2001547	[F]c4c([H])c([H])c(c2nc1c(nc([H])c([H])n1c2c3nc(nc([H])c3[H])N([H])C([H])([H])C(O[H])(C([H])([H])[H])C([H])([H])[H])C([H])([H])C([H])([H])[H])c([F])c4[H]	10,000	Inactive
CHEMBL1989265	O=C(O[H])c2c1[S]C([H])([H])[C@]([H])(n1c4c(c2=O)c([H])c([F])c(N3C([H])([H])C([H])([H])[C@]([H])(N([H])[H])C3([H])[H])c4[H])C([H])([H])[H]	10,000	Inactive
CHEMBL2004647	[H]n3c2nc(N1C([H])([H])C([H])([H])[C@@]([H])(N([H])C([H])([H])[H])C1([H])[H])c([Br])c(c2c(=O)c(c3[H])C(=O)O[H])C([H])([H])[H]	10,000	Inactive
CHEMBL1991434	O=C1ON=C(/C1=C(\[H])c2c([H])c([H])c(OC([H])([H])[H])c(OC([H])([H])[H])c2[H])c3c([H])c([H])c([Br])c([H])c3[H]	10,000	Inactive
CHEMBL1982980	[Cl]c3c([H])c([H])c([H])c(OC([H])([H])C([H])([H])C([H])([H])c2c1c([H])c([H])c([H])c(/C(=C(\C([H])([H])[H])C([H])([H])[H])C([H])([H])[H])c1n([H])c2C(=O)O[H])c3[Cl]	10,000	Inactive
CHEMBL1980142	O=C(N([H])c1nc(c([H])c([H])c1[H])C([F])([F])[F])N([H])c3c([H])c([H])nc2c(c([H])c([H])c([H])c23)C([F])([F])[F]	10,000	Inactive
CHEMBL1977148	O=C(N([H])c1c([H])c([H])c(OC([H])([H])[H])c([H])c1[H])N([H])c5c([H])c([H])c(c4c([H])[s]c3c(c2c([H])nn(c2[H])C([H])([H])C([H])([H])O[H])c([H])nc(N([H])[H])c34)c([H])c5[H]	10,000	Inactive
CHEMBL2000429	[F]c4c([H])c([H])c(c2nc1c(OC([H])(C([H])([H])[H])C([H])([H])[H])nc([H])c([H])n1c2c3nc(nc([H])c3[H])N([H])C([H])([H])C(C([H])([H])[H])(C([H])([H])[H])C([H])([H])O[H])c([F])c4[H]	10,000	Inactive
CHEMBL1987982	[F]C([F])([F])Oc4c([H])c([H])c(c3c([H])c([H])c2n([H])c([H])c(/C1=C(\[H])C([H])([H])N([H])C([H])([H])C1([H])[H])c2c3[H])c([H])c4[H]	10,000	Inactive
CHEMBL2006276	O=C2c4c(N([H])c1c(OC([H])([H])[H])c([H])c([H])c([H])c1N2[H])c([H])c(N([H])c3c([H])c([H])nc([H])c3[H])c([H])c4[H]	10,000	Inactive
CHEMBL1995740	O=[N+]([O-])c4c([H])c([H])c3N([H])c2c(c([H])c([H])c(c1c([H])c(OC([H])([H])[H])c(O[H])c([H])c1[H])c2[H])C(=O)N([H])c3c4[H]	10,000	Inactive
CHEMBL1979516	O=C(N([H])C([H])([H])C([H])([H])[H])c4c([H])nc(N([H])[H])c3c(c2c([H])c([H])c(N([H])C(=O)N([H])c1c([H])c([H])c([H])c([F])c1[H])c([H])c2[H])c([H])[s]c34	10,000	Inactive
CHEMBL1971141	O=C(N([H])c1c([H])c([H])c([H])c(c1[H])C([H])([H])[H])N([H])c3c([H])c([H])c(c2c([H])c([H])c([H])c(C(=O)N([H])[H])c2[H])c([H])c3[H]	10,000	Inactive
CHEMBL1998112	O=C(c4c(N([H])c2nc(c1c([H])c([H])c(OC([H])([H])[H])c(OC([H])([H])[H])c1[H])c([H])c3nc([H])c([H])n23)c([H])c([H])c([H])c4[H])N([H])[H]	10,000	Inactive
CHEMBL424872	O=C(c1nc(OC([H])([H])C([H])([H])[H])c(C#N)c(c1[H])N([H])[H])N([H])C([H])([H])c2c([H])c([H])c(c([H])c2[H])[S](=O)(=O)C([H])([H])[H]	10,000	Inactive
CHEMBL2006010	O=C(N([H])c1nc(c([H])c([H])c1[H])C([F])([F])[F])N([H])c3c([H])c([H])nc2c([Cl])c([Cl])c([H])c([H])c23	10,000	Inactive
CHEMBL1971430	O=c3c(c1c([H])c([H])c([H])c(c1[H])C([H])([H])[H])c(nc(N([H])C([H])([H])[C@@]([H])(N([H])[H])C([H])([H])c2c([H])c([H])c([H])c([H])c2[H])n3C([H])([H])[H])c4c([H])c([H])nc([H])c4[H]	10,000	Inactive
CHEMBL377408	O=C(c1nc(OC([H])([H])C([H])([H])[H])c(C#N)c(c1[H])N([H])[H])N([H])C([H])([H])c2c([H])c([H])c([H])c([H])c2[S](=O)(=O)N([H])[H]	10,000	Inactive
CHEMBL1988995	[F]c5c([H])c([H])c(c2nc1oc([H])c([H])n1c2c4nn3c(nnc3c([H])c4[H])C([H])(C([H])([H])[H])C([H])([H])[H])c([F])c5[H]	10,000	Inactive

CHEMBL1982383	[F]c4c([H])c([H])c(c2nc1c(nc([H])c([H])n1c2c3nc(nc([H])c3[H])N([H])C([H])([H])C(C([H])([H])[H])(C([H])([H])[H])C([H])([H])O[H])C([H])([H])C([H])([H])N([H])[H])c([H])c4[H]	10,000	Inactive
CHEMBL1973348	[H]n1c([H])c(c2c1c([H])c([H])c([H])c2[H])C([H])([H])[C@@]([H])(N([H])[H])C([H])([H])Oc5c([H])nc([H])c(c3c([H])c([H])c(N([H])[H])c(c3[H])C(=O)c4c([H])c([H])c([H])c([Cl])c4[H])c5[H]	10,000	Inactive
CHEMBL2004513	O=C(OC([H])([H])[H])c1c([H])c([H])c([H])c([H])c1c2c([H])c([H])c4c(c2[H])N([H])c3c([H])c([H])c([H])c([H])c3N([H])C4=O	10,000	Inactive
CHEMBL2002373	[H]OC([H])([H])C([H])([H])C([H])([H])n2c([H])nc(c1c([H])c([H])c([H])c([H])c1[H])c2c3c([H])c([H])nc([H])c3[H]	10,000	Inactive
CHEMBL1993941	O=C(N([H])c4c([H])c([H])c(c3c2c(nc([H])c(c1c([H])n(nc1[H])C([H])([H])C([H])([H])C([H])([H])[H])c2[s]c3[H])N([H])[H])c([H])c4[H])N([H])c5c([H])c([H])c([H])c([F])c5[H]	10,000	Inactive
CHEMBL1969735	[F]c4c([H])c([H])c(c2nc1c([Cl])nc([H])c([H])n1c2c3nc(nc([H])c3[H])N([H])C([H])([H])C(C([H])([H])[H])(C([H])([H])[H])C([H])([H])O[H])c([H])c4[H]	10,000	Inactive
CHEMBL2002682	[Cl]c1c([H])c([H])c([H])c(c1c3c([H])c([H])c([H])c2c(c(C(=O)N([H])C([H])([H])C([H])([H])OC([H])([H])C([H])([H])OC([H])([H])C([H])([H])N([H])[H])n([H])c23)C([H])([H])C([H])([H])C([H])([H])Oc5c([H])c([H])c([H])c4c([H])c([H])c([H])c([H])c45)C([H])(	10,000	Inactive
CHEMBL1982563	[F]c4c([H])c([H])c(c2nc1c(nc([H])c([H])n1c2c3nc(nc([H])c3[H])N([H])C([H])([H])C(C([H])([H])OC([H])([H])[H])(C([H])([H])[H])C([H])([H])[H])C([H])([H])[H])c([H])c4[H]	10,000	Inactive
CHEMBL1999714	O=C2N([H])c1c(c([H])c([H])c(c1[H])C([H])([H])[H])N([H])c3c2c([H])c([H])c([H])c3[H]	10,000	Inactive
CHEMBL2001957	[F]c4c([H])c([H])c(c2nc1c(OC([H])([H])C([F])([F])[F])nc([H])c([H])n1c2c3nc(nc([H])c3[H])N([H])C([H])([H])C(C([H])([H])[H])(C([H])([H])[H])C([H])([H])O[H])c([H])c4[H]	10,000	Inactive
CHEMBL1979855	O=C(N([H])c1c([H])c(nn1c2c([H])c([H])c([H])c(C(=O)N([H])C([H])([H])C#N)c2[H])C(C([H])([H])[H])(C([H])([H])[H])C([H])([H])[H])N([H])c4c([H])c([H])c([H])c3c([H])c([H])c([H])c([H])c34	10,000	Inactive
CHEMBL1995927	[Cl]c2c([H])c1nc(N([H])C([H])([H])C([H])([H])[H])c(nc1c([H])c2OC([H])([H])[H])N([H])C([H])([H])C([H])([H])[H]	10,000	Inactive
CHEMBL2003482	O=C(N([H])c1n([H])nc2c1C([H])([H])C([H])([H])C2([H])[H])C([H])([H])c3c([H])c([H])c([Cl])c([H])c3[H]	10,000	Inactive
CHEMBL1090360	O=C(N([H])c6c([H])c([H])c([H])c(c2nc1[s]c([H])c([H])n1c2c3nc(nc([H])c3[H])N([H])c5c([H])c([H])c([H])c(N4C([H])([H])C([H])([H])OC([H])([H])C4([H])[H])c5[H])c6[H])C([H])([H])c7c([H])c([H])c([H])c([H])c7[H]	10,000	Inactive
CHEMBL2001485	O=C(N([H])c1c([H])c([H])c(c([H])c1[H])C([H])([H])[H])N([H])c5c([H])c([H])c(c4c([H])[s]c3c(c2c([H])nn(c2[H])[C@@]([H])(C(=O)N(C([H])([H])[H])C([H])([H])[H])C([H])([H])[H])c([H])nc(N([H])[H])c34)c([H])c5[H]	10,000	Inactive
CHEMBL1977374	[F]c5c([H])c([H])c(c1nc4n(c1c3nn2c(nnc2c([H])c3[H])C([H])(C([H])([H])[H])C([H])([H])[H])C([H])([H])C([H])([H])C4([H])[H])c([F])c5[H]	10,000	Inactive
CHEMBL1981725	O=C(c6c([H])c([H])c([H])c(c2nc1c([H])c([H])c([H])c([H])n1c2c3nc(nc([H])c3[H])N([H])c5c([H])c([H])c(N4C([H])([H])C([H])([H])N(C([H])([H])C([H])([H])[H])C([H])([H])C4([H])[H])c([H])c5[H])c6[H])N([H])c7c([H])c([H])c([H])c([H])c7[F]	10,000	Inactive
CHEMBL210963	O=C(N([H])c1nc(OC([H])([H])C([H])([H])[H])c(C#N)c(c1[H])N([H])[H])C([H])([H])c2c([H])c(OC([H])([H])[H])c(c([H])c2OC([H])([H])[H])[S](=O)(=O)C([H])([H])[H]	10,000	Inactive
CHEMBL1614705	O=C(N4C([H])([H])C([H])([H])C([H])(c2n([H])nc(c1c([H])c([H])c([Cl])c([H])c1[F])c2c3nc([H])nc([H])c3[H])C([H])([H])C4([H])[H])C([H])([H])O[H]	10,000	Inactive
CHEMBL2006263	O=[S](=O)(N([H])c2c([H])c([H])c1nc(c(nc1c2[H])C([H])([H])[H])C([H])([H])[H])c3c([H])c([H])c(N([H])[H])c([H])c3[H]	10,000	Inactive
CHEMBL1966703	[H]n1nc(c3c1N([H])C2=C(C(=O)C([H])([H])C(C2([H])[H])(C([H])([H])[H])C([H])([H])[H])[C@@]3([H])c4c([H])c([H])c([H])c([H])c4[F])C([H])([H])[H]	10,000	Inactive

CHEMBL1975208	O=C(N([H])[C@]3([H])C([H])([H])C([H])([H])N(c2nc1n([H])c([H])c(C(=O)O[H])c(=O)c1c(c2[F])C([H])([H])[H])C3([H])[H])C([H])([H])[H]	10,000	Inactive
CHEMBL210618	O=C(N([H])c1nc(OC([H])([H])C([H])([H])[H])c(C#N)c(c1[H])N([H])[H])C([H])([H])c2c([H])c(OC([H])([H])[H])c([H])c([H])c2OC([H])([H])[H]	10,000	Inactive
CHEMBL183844	O=C(N([H])c1c([H])c([H])c([H])c(c1[H])C([H])([H])[H])N([H])c4c([H])c([H])c(c2c([H])c([H])c([H])c3c2C([H])([H])N([H])C3=O)c([H])c4C([H])([H])[H]	10,000	Inactive
CHEMBL1992242	O=C(N([H])c1c([H])c(c([H])c(c1[H])C([H])([H])[H])C([H])([H])[H])N([H])c4c([H])c([H])c(c3c([H])c([H])c([H])c2[s]nc([H])c23)c([H])c4[H]	10,000	Inactive
CHEMBL1090356	O=C(N([H])c6c([H])c([H])c([H])c(c2nc1[s]c([H])c([H])n1c2c3nc(nc([H])c3[H])N([H])c5c([H])c([H])c(N4C([H])([H])C([H])([H])OC([H])([H])C4([H])[H])c([H])c5[H])c6[H])C([H])([H])c7c([H])c([H])c([H])c([H])c7[H]	10,000	Inactive
CHEMBL1991138	[H]N([H])c4nc([H])c([H])n3c(nc(c2c([H])c([H])c([H])c(OC([H])([H])c1c([H])c([H])c([H])c([H])c1[H])c2[H])c34)[C@@]6([H])C([H])([H])[C@]([H])(C([H])([H])N5C([H])([H])C([H])([H])C([H])([H])C5([H])[H])C6([H])[H]	10,000	Inactive
CHEMBL1990288	[F]c5c([H])c([H])c(c2nc1oc([H])c([H])n1c2c3nc(nc([H])c3[H])N([H])[C@]([H])(c4c([H])c([H])c([H])c([H])c4[H])C([H])([H])[H])c([H])c5[H]	10,000	Inactive
CHEMBL1987359	O=C3c2c1c(c([H])c([H])c(N([H])C([H])([H])C([H])([H])C([H])([H])C([H])([H])[H])c1c([H])c([H])c2[H])C(=O)N3C([H])([H])C([H])([H])N([H])C([H])([H])C([H])([H])O[H]	10,000	Inactive
CHEMBL2003768	O=c2c(c(N([H])C([H])([H])c1c([H])c([H])c(OC([H])([H])[H])c([H])c1OC([H])([H])[H])c2=O)N([H])c3c([H])c([H])nc([H])c3[H]	10,000	Inactive
CHEMBL1988387	O=C(N([H])c4c([H])c([H])c(c3c2c(nc([H])c(c1c([H])n(nc1[H])C([H])([H])[H])c2[s]c3[H])N([H])[H])c([H])c4[H])N([H])c5c([H])c([H])c(OC([F])([F])[H])c([H])c5[H]	10,000	Inactive
CHEMBL1994040	[F]c5c([H])c([H])c(c2nc1c(nc([H])c([H])n1c2c3nc(nc([H])c3[H])N([H])C([H])([H])C(O[H])(C([H])([H])[H])C([H])([H])[H])C([H])([H])C4([H])C([H])([H])C4([H])[H])c([F])c5[H]	10,000	Inactive
CHEMBL1997503	O=C(O[H])[C@@]4([H])C([H])([H])C([H])([H])[C@@]([H])(N([H])c3c([H])c([H])c(c1onc(n1)c2c([H])c([H])c(OC([H])(C([H])([H])[H])C([H])([H])[H])c([Cl])c2[H])c([H])c3[H])C4([H])[H]	10,000	Inactive
CHEMBL1970314	O=C(N([H])[H])c3c([H])c(c1c([H])c([H])c([H])c(N([H])[H])c1[H])c([H])c2c([H])c([H])c([H])nc23	10,000	Inactive
CHEMBL1969537	[S]=c1n([H])nc3n1c2nn([H])c(=[S])n2c4[s]c5c(c34)C([H])([H])C([H])([H])C5([H])[H]	10,000	Inactive
CHEMBL2007044	[H]n2nc(c1c([H])c([H])c(C#N)nc1[H])c4c2c3c(c([H])c([H])c(C#CC([H])([H])N(C([H])([H])C([H])([H])[H])C([H])([H])C([H])([H])[H])c3[H])C4([H])[H]	10,000	Inactive
CHEMBL223460	O=[S](=O)(N([H])c2c([H])c1OC([H])([H])C([H])([H])C([H])([H])C([H])([H])C([H])([H])Oc3nc(N([H])C(=O)N([H])c1c([H])c2[Cl])c([H])nc3C#N)C([H])([H])[H]	10,000	Inactive
CHEMBL1995832	[F]c4c([H])c([H])c(c2nc1c(OC([H])([H])[H])nc([H])c([H])n1c2c3nc(nc([H])c3[H])N([H])C([H])([H])C(C([H])([H])[H])(C([H])([H])[H])C([H])([H])O[H])c([F])c4[H]	10,000	Inactive
CHEMBL1375418	[H]C([H])([H])c1c([H])c([H])c(c([H])c1[H])C([H])([H])N([H])c4nn2c(nnc2c3c([H])c([H])c([H])c([H])c3[H])c([H])c4[H]	10,000	Inactive
CHEMBL259922	O=C(c1c([H])c([H])c(c(c1[H])N([H])c2nc([H])nc3c2c([H])nn3c4c([H])c([H])c([H])c([H])c4[H])C([H])([H])[H])N([H])c5noc([H])c5[H]	10,000	Inactive
CHEMBL1983449	O=c2c(c(N([H])C([H])([H])c1c([H])c([H])c(c(c1[H])C([H])([H])[H])C([H])([H])[H])c2=O)N([H])c3c([H])c([H])nc([H])c3[H]	10,000	Inactive
CHEMBL1994321	O=C(N([H])c1c([H])c([H])c([H])c(c1[H])C([H])([H])[H])N([H])c4c([H])c([H])c(c3c([H])[s]c2c(C#CC([H])([H])N([H])[S](=O)(=O)C([H])([H])[H])c([H])nc(N([H])[H])c23)c([H])c4[H]	10,000	Inactive

CHEMBL2000508	[F]c4c([H])c([H])c(c2nc1[s]c([H])c([H])n1c2c3c([H])c([H])nc([H])c3[H])c([H])c4[H]	10,000	Inactive
CHEMBL2004419	[F]c2c1nc(n(c1c([H])c(c2[H])c4c(nc3[s]c([H])c([H])n34)c5c([H])c([H])c([F])c([H])c5[H])C([H])([H])C([H])(C([H])([H])[H])C([H])([H])[H])N([H])[H]	10,000	Inactive
CHEMBL2007372	[F]c4c([H])c([H])c(c2nc1c(nc([H])c([H])n1c2c3nc(nc([H])c3[H])N([H])C([H])([H])C(C([H])([H])[H])(C([H])([H])[H])C([H])([H])O[H])N([H])C([H])([H])[H])c([H])c4[H]	10,000	Inactive
CHEMBL1230122	O=c2n(c1nc(nc([H])c1c([H])c2Oc3c([H])c([H])c([F])c([H])c3[F])N([H])C4([H])C([H])([H])C([H])([H])OC([H])([H])C4([H])[H])C([H])([H])[H]	10,000	Inactive
CHEMBL1996234	[H]C([H])([H])[C@]6(O[H])C([H])([H])[C@]([H])(c5nc(c2c([H])c([H])c1c([H])c([H])c(nc1c2[H])c3c([H])c([H])c([H])c([H])c3[H])c4c(nc([H])c([H])n45)N([H])[H])C6([H])[H]	10,000	Inactive
CHEMBL1968127	[F]c4c([H])c([H])c(c2nc1oc([H])c([H])n1c2c3nc(nc([H])c3[H])N([H])C([H])([H])C(C([H])([H])N(C([H])([H])[H])C([H])([H])[H])(C([H])([H])[H])C([H])([H])[H])c([H])c4[H]	10,000	Inactive
CHEMBL1970735	O=C(N([H])c1nc(OC([H])([H])C([H])([H])C([H])([H])[H])c(C#N)c(c1[H])N([H])[H])C([H])([H])[H]	10,000	Inactive
CHEMBL246970	[Cl]c1c(c([H])c([H])c([H])c1[H])C([H])([H])N([H])c3nc(N([H])C([H])([H])[C@@]2([H])C([H])([H])C([H])([H])[C@@]([H])(C([H])([H])N([H])[H])C([H])([H])C2([H])[H])c(c([H])n3)[N+](=O)[O-]	10,000	Inactive
CHEMBL1968791	[H]C([H])([H])c4c(c2c([H])c([H])c([H])c(OC([H])([H])c1c([H])c([H])c([H])c([H])c1[H])c2[H])c3c(nc([H])nc3n4[C@]6([H])C([H])([H])[C@]([H])(C([H])([H])N5C([H])([H])C([H])([H])C([H])([H])C5([H])[H])C6([H])[H])N([H])[H]	10,000	Inactive
CHEMBL1967116	O=C(N([H])c1c([H])c([H])c([H])c(c1[H])C([H])([H])[H])N([H])c5c([H])c([H])c(c4c([H])[s]c3c(c2c([H])nn(c2[H])C([H])([H])[H])c([H])nc(N([H])[H])c34)c([H])c5[H]	10,000	Inactive
CHEMBL2004716	O=C(N([H])C([H])([H])C([H])([H])N(C([H])([H])C([H])([H])[H])C([H])([H])C([H])([H])[H])C([H])=C([H])c4c([H])nc(N([H])[H])c3c(c2c([H])c([H])c(N([H])C(=O)N([H])c1c([H])c([H])c([H])c(c1[H])C([H])([H])[H])c([H])c2[H])c([H])[s]c34	10,000	Inactive
CHEMBL1999496	O=C(N([H])c1c([H])c([H])nc([H])c1[H])[C@@]4([H])C(c2c([H])c([H])c([H])c(c2[H])C([H])([H])[H])(c3c([H])c([H])c([H])c(c3[H])C([H])([H])[H])C4([H])[H]	10,000	Inactive
CHEMBL2004871	[F]c3c([H])c([H])c(c1nc([H])n(c1c2c([H])c([H])nc([H])c2[H])C([H])([H])C([H])([H])C([H])([H])O[H])c([H])c3[H]	10,000	Inactive
CHEMBL1985153	O=C(O[H])c3c(N([H])c1nc(nc([H])c1[H])N([H])[C@@]2([H])[C@@]([H])(N([H])[H])C([H])([H])C([H])([H])C([H])([H])C2([H])[H])c([H])c([H])c([H])c3[H]	10,000	Inactive
CHEMBL1999321	O=c4c(N([H])C([H])([H])C([H])([H])c2c([H])c([H])c(Oc1c([H])c([H])c([H])c([H])c1[H])c([H])c2[H])c(N([H])c3c([H])c([H])nc([H])c3[H])c4=O	10,000	Inactive
CHEMBL2005375	O=C2N(c1c([H])nc(nc1N([C@@]2([H])C([H])([H])C([H])([H])[H])[C@]3([H])C([H])([H])C([H])([H])C([H])([H])C3([H])[H])N([H])c5c([H])c([H])c(C(=O)N([H])[C@@]4([H])C([H])([H])C([H])([H])C([H])([H])C([H])([H])C4([H])[H])c([H])c5OC([H])([H])[H	10,000	Inactive
CHEMBL1998193	O=C(N([H])C([H])([H])C([H])([H])C([H])([H])C([H])([H])C([H])([H])C([H])([H])N([H])C(=O)N([H])c1c([H])c([H])c([H])nc1[H])N([H])c2c([H])c([H])c([H])nc2[H]	10,000	Inactive
CHEMBL1982866	[Cl]c3c([H])c([H])c(c1nn(c([H])c1c2nc([H])nc([H])c2[H])C([H])([H])C([H])([H])OC([H])([H])[H])c([H])c3[H]	10,000	Inactive
CHEMBL1972659	O=C(N([H])N=C2c1c([H])c([H])c(O[H])c(c1C([H])([H])C([H])([H])C2([H])[H])C([H])([H])[H])C([H])([H])c4c([H])c([H])c([H])c3c([H])c([H])c([H])c([H])c34	10,000	Inactive
CHEMBL248757	N#Cc1nc([H])c4nc1OC([H])([H])C([H])([H])C([H])([H])C([H])([H])Oc3c([H])c(N([H])C([H])([H])c2[s]c([H])nc2[H])c([Cl])c([H])c3N([H])C(=O)N4[H]	10,000	Inactive
CHEMBL363648	O=C(N([H])c3nc([H])c([H])c(c1[s]c(nc1c2c([H])c([H])c([H])c(c2[H])C([H])([H])[H])C([H])([H])C([H])([H])[H])c3[H])c4c([H])c([H])c([H])c([H])c4[H]	10,000	Inactive

CHEMBL1971149	O=C(O[H])C(C([H])([H])N([H])c1nc([H])c([H])c([H])c(n1)c3n2c([H])c([H])nc(c2nc3c4c([H])c([H])c([F])c([H])c4[F])C([H])([H])[H])(C([H])([H])[H])C([H])([H])[H]	10,000	Inactive
CHEMBL1991143	O=c3c2c(nn(c1c([H])c([H])c(c([H])c1[H])C([H])([H])[H])c2n(O[H])c4c3c([H])c([Cl])c([H])c4[H])C([H])([H])[H]	10,000	Inactive
CHEMBL1999590	[H]C([H])([H])c1nn(c(c1[H])c2c([H])c([H])c([H])c([H])c2[H])c3nc([H])nc(c3[H])N([H])N=C([H])c4oc([H])c([H])c4[H]	10,000	Inactive
CHEMBL1971186	[F]c5c([H])c([H])c(c2nc1oc([H])c([H])n1c2c3nc(nc([H])c3[H])N([H])C4([H])C([H])([H])C([H])([H])N([H])C([H])([H])C4([H])[H])c([H])c5[H]	10,000	Inactive
CHEMBL1991429	O=C(N([H])c6c([H])c([H])c([H])c(c2nc1[s]c([H])c([H])n1c2c3nc(nc([H])c3[H])N([H])c5c([H])c([H])c([H])c(N4C(=O)C([H])([H])C([H])([H])C4([H])[H])c5[H])c6[H])C([H])([H])c7c([H])c([H])c([H])c([H])c7[H]	10,000	Inactive
CHEMBL1995916	O=C(N([H])C([H])([H])[H])c1nn([H])c(c1[H])c2c([H])c([H])c([H])c([H])c2[H]	12,589.20	Inactive
CHEMBL1969156	[H]N([H])c3nc([H])c([H])c2c([H])nc(c1c([H])c([H])c([H])c([H])c1[H])n23	12,589.20	Inactive
CHEMBL2003545	O=C(OC([H])([H])[H])c2c([H])[s]c1c([H])c([H])c(=O)n([H])c12	12,589.20	Inactive
CHEMBL1965683	[H]n1nc([H])c2c1C([H])([H])C([H])([H])[C@@]2([H])c3c([H])c([H])c([H])c([H])c3[H]	12,589.20	Inactive
CHEMBL2002480	[H]N([H])c3nc2nc([H])c(c1c([H])c([H])c([H])c([H])c1[H])c([H])n2n3	15,848.90	Inactive
CHEMBL1997119	[H]N([H])c3nc([H])c2c([H])c([H])c([H])c(c1c([H])c([H])c([H])c([H])c1[H])n23	15,848.90	Inactive
CHEMBL1178727	[H]N([H])c1nc2c([s]1)C([H])([H])c3c([H])c([H])c([H])c([H])c23	15,848.90	Inactive
CHEMBL1989423	[H]n3nc2c1c([H])c([H])c([H])c([H])c1C([H])([H])c2c3[H]	19,952.60	Inactive
CHEMBL1968460	[H]C([H])([H])n1c(nc2[s]c([H])c([H])c12)N([H])[H]	19,952.60	Inactive
CHEMBL1992673	O=c3n([H])c1c([H])c([H])c([Cl])c([H])c1c2nc([H])nn23	19,952.60	Inactive
CHEMBL1971785	[H]n1c(c(c(=O)oc1=O)c2c([H])c([H])c([H])c([H])c2[H])C([H])([H])[H]	19,952.60	Inactive
CHEMBL2000879	[H]n1nc([H])c3c1C([H])([H])C([H])([H])[C@]([H])(c2c([H])c([H])c([H])c([H])c2[H])C3([H])[H]	25,118.90	Inactive
CHEMBL1992607	O=C2c1c(nc([H])nc1N([H])C([H])([H])C([H])([H])N2[H])N([H])[H]	25,118.90	Inactive
CHEMBL2002802	O=C(c2[s]c1nc(n(c1c2[H])C([H])([H])[H])N([H])[H])N([H])[H]	25,118.90	Inactive
CHEMBL1727312	O=C2N([H])c1c([H])c([H])c([H])c([H])c1N([H])c3c([H])c([H])c([H])c([H])c23	25,118.90	Inactive
CHEMBL2001704	[H]N([H])c3nnc2[s]c1c([H])c([H])c([H])c([H])c1n23	31,622.80	Inactive

CHEMBL1965838	[H]C([H])([H])c2c([H])[s]c1nc(nn12)N([H])[H]	31,622.80	Inactive
CHEMBL2003952	[H]n1nc(c(c(c1=O)N([H])[H])C(O[H])(C([H])([H])[H])C([H])([H])[H])c2c([H])c([H])c([H])c([H])c2[H]	31,622.80	Inactive
CHEMBL2001378	[H]N([H])c2nnc(c1c([H])c([H])c([H])c([H])c1[H])c(n2)N([H])[H]	39,810.70	Inactive
CHEMBL1975911	O=c3n([H])c([H])c([H])c2c([H])nc(c1c([H])c([H])c([H])c([H])c1[H])n23	39,810.70	Inactive
CHEMBL1971679	O=C(O[H])c2c([H])nc1[s]c([H])c([H])n1c2=O	39,810.70	Inactive
CHEMBL1980853	N#Cc1c([H])nc([Cl])nc1N([H])[H]	50,118.70	Inactive
CHEMBL1970873	O=C2c1c(c([H])c([H])c(c1[H])C([H])([H])[H])N([H])c3c(N2[H])c([H])c([H])c([H])c3[H]	50,118.70	Inactive
CHEMBL1998228	[Cl]c3nc([H])n1c(nnc1c2c([H])c([H])c([H])c([H])c2[H])c3[H]	63,095.70	Inactive
CHEMBL1967612	O=C1N([H])[S](=O)(=O)c2c([H])c([H])c([Cl])c([H])c12	63,095.70	Inactive
CHEMBL1991251	[H]N([H])c1nonc1n2nnc(c2[H])C([H])([H])O[H]	63,095.70	Inactive
CHEMBL1966799	[Cl]c2c([H])c([H])c([H])c1nnc(N([H])[H])n12	63,095.70	Inactive
CHEMBL1991008	[H]n1nc(N([H])[H])c3c1c(c2c([H])c([H])c([H])c([H])c2[H])c([H])c([H])c3[H]	79,432.80	Inactive
CHEMBL1994808	N#Cc1nc(nnc1c2c([H])c([H])c([H])c([H])c2[H])C([H])([H])[H]	79,432.80	Inactive
CHEMBL2004887	[H]N([H])c3nc([H])c([H])c2nc(c1c([H])c([H])c([H])c([H])c1[H])c([H])n23	79,432.80	Inactive
CHEMBL1980003	[H]C([H])([H])c1c([H])c([H])c([H])c([H])c1c3c([H])c([H])c([H])c2nc([H])c([H])n23	79,432.80	Inactive

Table S6: Ordered NCI compounds, their fit values against the GFA-SVR ML-QSAR selected pharmacophores, their ML activity prediction and the percentage of inhibition at 10 μM

					LRCFs			Fit values against				
**Hit**	**NCI Code**	**Structure**	**CHI_2**	**Num Rings 6**	**LEU$_{244}$ HN^{LD}**	**ASP$_{325}$ HA^{LD}**	**VAL$_{324}$ HB^{LD}**	**Hypo (5-R2-08)**	**Hypo (6-R2-07)**	**Hypo (8-R3-08)**	**Prediction IC$_{50}$ (nM)**	**% Inhibition at 10 μM (IC$_{50}$ nM)**
**92**	9334	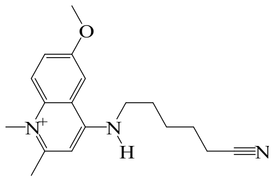	8.8	2	0	0	0	7.3	0.0	6.9	191.0	18
**93**	9336	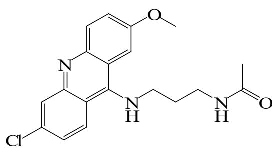	10.8	3	1	0	0	6.1	3.5	4.7	178.0	65
**94**	13633	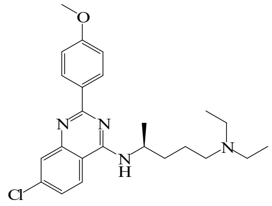	12.5	3	0	0	0	7.7	6.6	0.0	145.0	1
**95**	55230	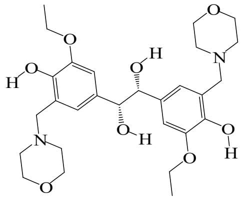	16.0	4	0	0	0	6.5	2.4	6.9	276.0	2
**96**	57129	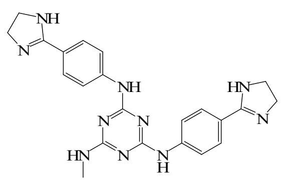	13.8	3	0	0	0	5.7	4.9	3.7	171.0	99 (57.5)
**97**	127626	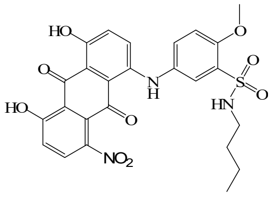	16.9	4	0	0	0	5.9	2.7	7.4	271.0	64
**98**	127681	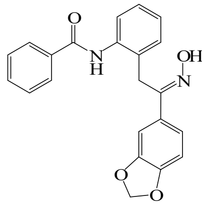	11.8	3	0	0	0	5.7	3.3	7.3	216.0	24
**99**	134169	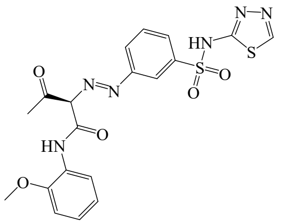	14.0	2	0	0	0	5.6	2.9	7.24	250.0	43

Table S6: Ordered NCI compounds, their fit values against the GFA-SVR ML-QSAR selected pharmacophores, their ML activity prediction and the percentage of inhibition at 10 μM

Hit	NCI Code	Structure	CHI_2	Num Rings 6	LRCFs			Fit values against			Prediction IC_{50} (nM)	% Inhibition at 10 µM (IC_{50} nM)
					LEU_{244} HN^{LD}	ASP_{325} HA^{LD}	VAL_{324} HB^{LD}	Hypo (5-R2-08)	Hypo (6-R2-07)	Hypo (8-R3-08)		
**100**	164472	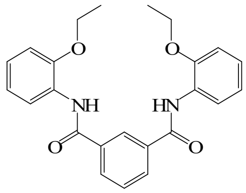	12.3	3	0	0	0	5.69	1.82	7.24	240.0	3
**101**	166726	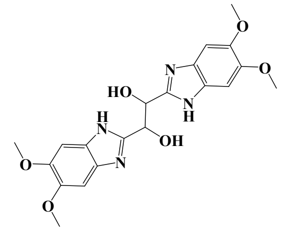	12.8	2	1	0	1	7.74	3.14	3.35	188.0	11
**102**	324572	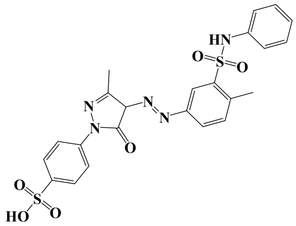	16.9	3	0	0	0	6.45	2.78	7.61	269.0	23
**103**	342048	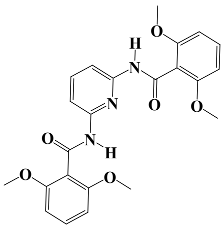	13	3	0	0	1	5.23	1.95	7.25	320.0	3
**104**	367067	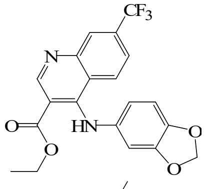	13.3	3	0	0	0	7.40	1.82	4.96	195.0	52
**105**	371713	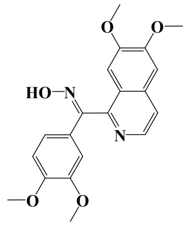	10.8	3	0	0	0	7.05	5.68	1.52	95.0	2
**106**	409969	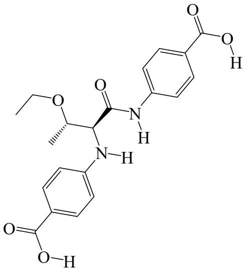	11.9	2	1	0	0	5.71	5.60	4.72	60.0	4
**<u>107</u>**	645812	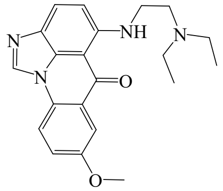	11.4	3	0	0	0	5.90	2.36	6.23	252.0	76 (1717)

Table S6: Ordered NCI compounds, their fit values against the GFA-SVR ML-QSAR selected pharmacophores, their ML activity prediction and the percentage of inhibition at 10 μM.

Hit	NCI Code	Structure	CHI_2	Num Rings 6	LRCFs			Fit values against			Prediction IC_{50} (nM)	% Inhibition at 10 μM (IC_{50} nM)
					LEU_{244} HN^{LD}	ASP_{325} HA^{LD}	VAL_{324} HB^{LD}	Hypo (5-R2-08)	Hypo (6-R2-07)	Hypo (8-R3-08)		
**108**	653145	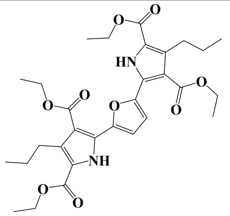	16.6	0	1	0	0	3.14	5.10	7.23	264.0	0
**<u>109</u>**	660824	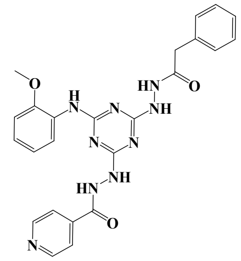	15.1	4	1	1	0	6.70	3.89	7.72	221.0	94 (272.9)
**110**	669887	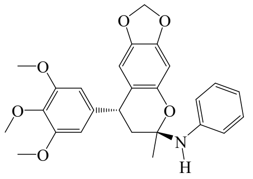	14.6	4	0	0	0	5.29	3.58	5.15	232.0	3
**111**	670676	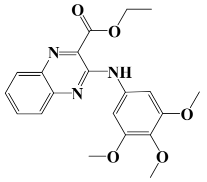	11.4	3	0	0	0	6.48	5.37	3.21	88.0	2
**112**	671988	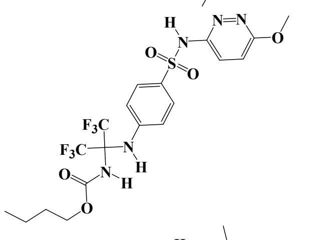	16.8	2	0	0	0	6.58	3.16	7.60	269.0	2
**113**	672085	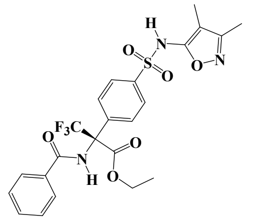	16.5	2	1	0	0	4.15	3.75	7.57	261.0	1
**114**	675782	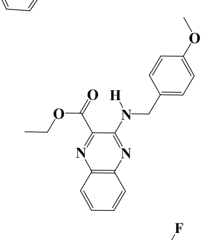	10.3	3	0	0	0	6.57	6.87	3.25	65.0	33
**115**	734059	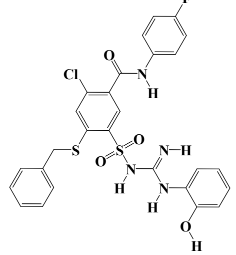	17.5	4	0	0	0	6.49	4.34	7.87	264.0	20
